# Supplementary material for: Obtaining and Documenting Informed Consent: An Advanced UME Cross-Specialty, Role-Playing Skill Builder
Source: MedEdPORTAL. 2026 Mar 3;22:11580. doi: 10.15766/mep_2374-8265.11580 (PMC12956033; doi:10.15766/mep_2374-8265.11580)

**Informed Consent Syllabus**

Students are provided this syllabus prior to the synchronous course and instructed to read the following and complete the necessary pre-work as described below.

**Overview:**

Informed consent (IC) is an essential standard of care practice for physicians. The AAMC strongly suggests medical students learn and become proficient in obtaining IC, core entrustable professional activity (EPA) 11. This course builds upon clerkship-level experiences with obtaining informed consent by utilizing pre-class preparation, a large group orientation, and small-group role-play as a physician obtaining and documenting informed consent for an assigned new medication or procedure. All students will discuss their experience with peers and small group faculty preceptor and receive immediate feedback on their performance from their group. The session will end with a large-group review and question and answer session that includes all students and a panel of faculty-physician experts. This active practice session is intended to improve comfort and performance in obtaining informed consent, as well as increase student fund of knowledge about the process and the medications/procedures discussed. Throughout the next several years, as you gain more experience, you will get better at learning and providing medical expertise. During B3 and your 4^th^ year, we encourage you to focus on the essential COMPONENTS as well as HOW to obtain informed consent in a more collaborative and easy style.

**Goals and Objectives:**

1. Learn essential elements of obtaining Informed Consent

2. Utilize an essential steps rubric for obtaining and documenting Informed Consent for medications and procedures

3. Practice shared decision-making techniques about a proposed treatment plan via supervised role-play

4. Propose evidence-based therapeutic options for assigned vignette patients

5. Assess and discuss self and peer performance in obtaining and documenting informed consent

**Background:**

This activity is designed to help you develop and to improve your ability to obtain informed consent for procedures/interventions and medications and introduce you to documenting that discussion in the medical record. Being able to explain procedures and medications, as well as their potential benefits and risks to patients is at the core of what we do in medicine.

**Overview of Prep and Session Participation:**

PRIOR to COURSE (see “PREP WORK” below):

Students will be assigned either a medication or procedure vignette and are expected to use the assigned readings and the “How To Obtain and Document Informed Consent Guide” to complete the Informed Consent Template sheet on their own BEFORE the session.

COURSE DAY:

After the course directors full-class Orientation, students will work with faculty preceptors in small groups of 9-12 students. In the small group sessions, students will divide up into several sets of 3 students. Each peer group of 3 students will spend time rotating through obtaining informed consent role play as the physician, the patient, and an observer-scribe. It is important that each set of 3 students is NOT composed of ALL medication or ALL procedure vignettes for increased exposure to the differences in these processes.

Each student will be the “MD/Intern” for whichever medication or procedure assigned, and the others in your "mini-group" will either role-play the part of the patient in your assigned vignette or observe and scribe.

ALL students in the small group will role-play the physician Obtaining Informed Consent at least once. Students not actively engaged in the role play as MD or patient will be peer observers and should fill out the peer feedback form.

After each role play, that student’s IC documentation note will be reviewed and edited by the mini-group. Your faculty will set the "tempo" for the session with you. You may all role-play in succession before the whole small group, or your mini-groups may role-play simultaneously while your faculty rotates to observe each group throughout the session.

The entire small group will spend time at the end of the role-playing to self-reflect, discuss observed/new strategies, review a few of the written notes as a group, developing “Informed Consent Pearls” for the large group report session.

The course will finish in the large group room, reviewing “Pearls” and attending to a Q&A session

Things to consider as you hone your skills:

- What words should you use? “Taper” versus “decrease”?
- How to alter your vocabulary for different patients?
- What should the patient do for the side effects or adverse reactions or complications that you describe? Call you? Call 911? Watch and wait?
- When will the patient see you again? What is routine? Urgent?
- What side effects / labs / etc. will YOU, the doctor, be watching out for, and how do you include the patient in the watching?
- Practice shared decision-making…Ask the patient what they think/know and engage them in the process
- Practice “teach back” by asking the patient to repeat or teach you what you have described to them
- Develop a clear side-effect/emergency/follow up plan that is understood by the patient
- How do you condense all of this for a note in the medical record?

**PREP-WORK: (about 90min) reading, online learning, and prepping assigned vignette**

1. Review “StatPearls” Informed Consent
2. Complete/review the ADMSEP eModule on Informed Consent
3. Review “How to Obtain and Document Informed Consent Guide”
4. Prepare YOUR ASSIGNED IC Template following the rubric
   - 1. PRE-fill the background information for obtaining and documenting informed consent and use ANY sources to complete the IC-Mes or IC-Procedure Template, to include making an outline/draft your DOCUMENTATION NOTE for your assigned medication or procedure for the roleplay exercises. Each student is assigned *either* a med or procedure vignette to prepare.

5. Print out 3-4 observer-scribe informed consent feedback forms (a limited # will be available that day)

**Informed Consent Course Schedule:**

**0730-0755: 25 minutes- Introduction and Overview**

Course directors, all faculty, and all students

**0800-1030: Role Play, Practice Notes with Peer/Faculty Review**

Faculty preceptors and 9-10 students

*Track what worked/what you want to work on for 1140 report-out

**1030-1115: Small Group Review of Notes and Group List of Important Pearls**

Faculty preceptors and 9-10 students

**1115-1130: Break and room change**

**1130-1215: Large Group Report, Recap, Question and Answers**

Course directors, all faculty, and all students

**Informed Consent Mnemonics/Rubric:**


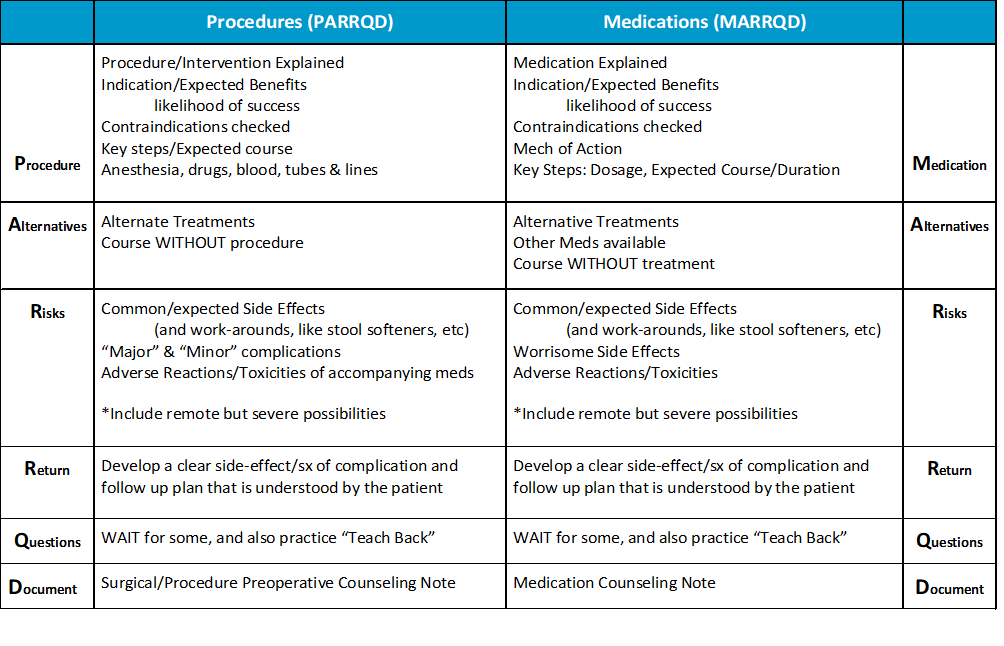

Supplement: Supplementary file 1 — Course Syllabus.docxPrereadings.pdfStatPearls Article.pdfADMSEP eModule folderClinical Vignettes.pdfRubric.pdfMARRQD, PARRQD Templates.docxOrientation.pptxObserver-Scribe Template.docxVignette Answers.pdf [file mep_2374-8265.11580-s001.zip › A. Course Syllabus.docx]
