# Supplementary material for: Obtaining and Documenting Informed Consent: An Advanced UME Cross-Specialty, Role-Playing Skill Builder
Source: MedEdPORTAL. 2026 Mar 3;22:11580. doi: 10.15766/mep_2374-8265.11580 (PMC12956033; doi:10.15766/mep_2374-8265.11580)
Supplement: Supplementary file 1 — Course Syllabus.docxPrereadings.pdfStatPearls Article.pdfADMSEP eModule folderClinical Vignettes.pdfRubric.pdfMARRQD, PARRQD Templates.docxOrientation.pptxObserver-Scribe Template.docxVignette Answers.pdf [file mep_2374-8265.11580-s001.zip › B. Prereadings.pdf]

Informed Consent Pre-Readings

Students are to be provided the below pre-readings prior to the synchronous informed consent course.

1. StatPearls "Informed Consent" article (Appendix D)
2. ADMSEP eModule on Informed Consent (Appendix E)
3. EEC (see next page)

Essential Elements of Communication

One of the most important parts of working with patients is effective commuication. This quick reference tool is based on extensive research and will help you find the "must do" and "should do" behaviors that will help make you an excellent communicator.

OPENING THE DISCUSSION		
	MUST DO	*SHOULD DO*
Introduction	o Use a polite greeting<br>o Determine and use patients preferred name<br>o Accurately introduce self with full name and title	o Show genuine interest<br>o Display welcoming nonverbal behavior
Patient opening	o Begin with open-ended question<br>o Do not interrupt initial response	o Ask if there is anything else patient wants to add after they finish initial statement<br>o Summarize patients opening concerns and verify facts
Agenda setting	o Offer a brief outline of what to expect	o Offer detailed outline of what to expect<br>o Verify agenda with patient

BUILDING A RELATONSHIP		
	MUST DO	*SHOULD DO*
Listening	o Actively listen<br>o Accept correction<br>o Use previous information as basis for subsequent questions<br>o Interrupt rarely<br>o Summarize at least once	o Acquire and accurately assimilate the facts and subtleties of patient's situation<br>o Do not interrupt important silences<br>o Use restatements, summaries, or explicit checks to verify information
Empathy & attitude	o Demonstrate or express appropriate concern for patient	o Respond appropriately to patient's concerns or issues<br>o Provide nonjudgmental support<br>o Help patient clarify their own feelings and thoughts<br>o Express genuine concern throughout the encounter
Nonverbal behavior	o Have awareness of what non-verbals you are communicating to patient<br>o Make appropriate eye contact	o Consistently indicate interest and concern through tone of voice and facial expressions<br>o Use receptive posture<br>o Make mutually agreeable adjustments in distance or touch for patient's comfort

GATHERING INFORMATION		
	MUST DO	***SHOULD DO***
**Context**	o Acquire information about the patient as a person<br>o Briefly explore patient's life context	o Encourage patient to share their reasons for seeking medical attention
**Questions**	o Balance open- and close-ended questions appropriately<br>o Use close-ended questions to check details	o Tailor questions to patient as an individual<br>o Prompt patient to talk freely in response to open-ended questions<br>o Clarify specific information or details through close-ended questions
**Transitions**	o Explain transitions<br>o Occasionally backtrack to omitted or forgotten questions	o Use smooth transitions<br>o Explicit and implicit transitions are clear and logical
**Physical exam**	o Explain examination maneuvers<br>o Alert patient before performing private or sensitive maneuvers	o Establish dialogue about sensations resulting from the examination
**Personal privacy**	o Use adequate draping<br>o Assure privacy<br>o Check on patient comfort	o Drape respectfully

UNDERSTANDING THE PATIENT'S PERSPECTIVE		
	MUST DO	***SHOULD DO***
**Patient concerns**	o Ask patient about their concerns at some point in the interview<br>o Follow up on patient concerns explicitly	o Ask patient about their concerns early in the encounter<br>o Consistently follow up on clues or information from the patient
**Patient beliefs & preference**	o Ask about patient beliefs, preferences, and requests<br>o Acknowledge patient perspective	o Acknowledge patient beliefs, preferences, and requests<br>o Responds to patient perspective as understandable and valid
**Expression of feelings**	o Recognize and acknowledge explicit expression of emotions<br>o Ask about patient emotions	o Facilitate the expression of patient's feelings<br>o Anticipate emotional reactions of patients<br>o Elicit means of patient emotional support

SHARING INFORMATION		
	MUST DO	***SHOULD DO***
Vocabulary	o Use language appropriate to patient's educational or intellectual level<br>o Clarify vocabulary upon request	o Check patient's understanding of technical words and explain as necessary<br>o Skillful use of technical vocabulary
Patient understanding	o Acknowledge when patient volunteers their understanding of their illness or situation	o Ask about patient's understanding of their illness or situation<br>o Highlight areas of similarity between patient's understanding and medical science
Clinician information & explanation	o Give information that is specific and clear	o Give full, clear, and thorough explanation of what patient's symptoms might mean or how they could be investigated<br>o Verify patient's understanding of information<br>o Offer to provide additional information

REACHING AGREEMENT ON THE PLAN		
	MUST DO	***SHOULD DO***
**Negotiation**	o Present a plan and request feedback	o Solicit input, negotiate, and confirm final plan with patient
**Implementation**	o Address patient hesitations, suggestions, or questions about implementing plan	o Elicit patient suggestions or questions about implementing the plan<br>o Explore barriers to implementing the plan and facilitate possible solutions

PROVIDING CLOSURE		
	MUST DO	***SHOULD DO***
**Next steps**	o Provide clear explanation of next steps	o Verify next steps with patient (e.g. get dressed, wait in room, make another appointment, talk to attending, etc.)
**Physician conclusion**	o Polite, generic conclusion	o Polite, personalized, thoughtful conclusion

This tool is based on the Essential Elements of Communication, Univeristy of New Mexico School of Medicine. (2005)
