# Supplementary material for: Obtaining and Documenting Informed Consent: An Advanced UME Cross-Specialty, Role-Playing Skill Builder
Source: MedEdPORTAL. 2026 Mar 3;22:11580. doi: 10.15766/mep_2374-8265.11580 (PMC12956033; doi:10.15766/mep_2374-8265.11580)
Supplement: Supplementary file 1 — Course Syllabus.docxPrereadings.pdfStatPearls Article.pdfADMSEP eModule folderClinical Vignettes.pdfRubric.pdfMARRQD, PARRQD Templates.docxOrientation.pptxObserver-Scribe Template.docxVignette Answers.pdf [file mep_2374-8265.11580-s001.zip › D. ADMSEP eModule folder/story.html]

Informed Consent\_MedEdPortal no javascript 20250625


Informed Consent\_MedEdPortal no javascript 20250625

You are offline. Trying to reconnect...
