# Supplementary material for: Obtaining and Documenting Informed Consent: An Advanced UME Cross-Specialty, Role-Playing Skill Builder
Source: MedEdPORTAL. 2026 Mar 3;22:11580. doi: 10.15766/mep_2374-8265.11580 (PMC12956033; doi:10.15766/mep_2374-8265.11580)
Supplement: Supplementary file 1 — Course Syllabus.docxPrereadings.pdfStatPearls Article.pdfADMSEP eModule folderClinical Vignettes.pdfRubric.pdfMARRQD, PARRQD Templates.docxOrientation.pptxObserver-Scribe Template.docxVignette Answers.pdf [file mep_2374-8265.11580-s001.zip › E. Clinical Vignettes.pdf]

Informed Consent MEDICATION Vignettes

Students are provided the following medication and procedure vignettes as part of the informed consent pre-work and assigned one for which to complete an informed consent card to use for the synchronous roleplay session.

1. **Eszopiclone (Lunesta): Med-Psych**
   Patient is a 52-year-old woman with h/o hypothyroidism, recently diagnosed with obstructive sleep apnea, and placed on CPAP by the sleep medicine clinic. She has an appointment in the Sleep Clinic with a chief complaint of difficulty sleeping, specifically falling asleep and staying asleep with the CPAP on, feeling unrested and irritable in the morning upon awakening. She also has tried following the CBT for insomnia app suggestions but is unable to work through the wearing of her CPAP. She has no history of depression, anxiety, or substance misuse/abuse. She has no other medical problems and has medical decision-making capacity. You and your attending have determined that a trial of eszopiclone might be helpful in treating her sleep difficulty and assist in her use of CPAP.
   a. Complete your "Informed Consent" study/prep card
   b. Be prepared to role-play the role of the intern/physician in obtaining informed consent
   c. Write an Informed Consent Note for the electronic medical record (EMR) AFTER the role play (hint: develop an outline for this case for your use)

2. **Oral Contraceptives: OB-GYN**
   Patient is a 32 y/o female who presents to your office desiring contraception. You discuss her reproductive health plans and review her prior contraceptive use. Previously, she used Depo medroxyprogesterone but did not like some of the side effects and she now desires an alternative method. She consistently uses condoms to reduce the risk of sexually transmitted infections. She has no significant medical history and is in good health. Through shared decision-making and after review of her contraceptive options she desires to begin using oral combined hormonal contraceptive pills.
   a. Complete your "Informed Consent" study/prep card
   b. Be prepared to role-play the role of the intern/physician in obtaining informed consent
   c. Write an Informed Consent Note for the electronic medical record (EMR) AFTER the role play (hint: develop an outline for this case for your use)

3. **Alteplase: Neuro-ED**
   A 34 y/o active duty SSG is in the ED after developing left arm and leg weakness while eating breakfast this morning. He woke at 05:30, performed his usual PT and went to the DFAC around 07:45. He developed worsened weakness, and he arrived in the ED around 08:30. His neuro exam was notable for 4/5 strength of the left arm and leg, (+) pronator drift on the left and reflexes were 3 on the left and 2 on the right. Neurology saw the patient at 08:50 and feel this patient is a good thrombolysis candidate. You are asked to obtain informed consent on this patient for the administration of alteplase. He has no significant medical history and is in good health. He is able to engage in shared decision making.
   a. Complete your "Informed Consent" study/prep card
   b. Be prepared to role-play the role of the intern/physician in obtaining informed consent
   c. Write an Informed Consent Note for the electronic medical record (EMR) AFTER the role play (hint: develop an outline for this case for your use)

INTERVENTION/PROCEDURE Vignettes

1. **Insertion of a PICC-Line for IV Antibiotics: Peds-ID**
   This patient is a 14-year-old male with a hx of trauma to the left knee during soccer practice. His knee is warm, swollen, and moderately tender with weight bearing on walking. It was drained by orthopedics 2 days ago who thought it was primarily a traumatic hematoma, and the patient had a reduction but not complete relief of symptoms. Though the fluid evaluation looked like blood on gram stain and cell counts, it grew a methicillin-sensitive *Staphylococcus aureus* within 24 hours. Orthopedics does not think it is serious enough for another procedure but on consulting with pediatric infectious diseases agrees to letting them begin IV therapy with a first-generation cephalosporin. The peripherally inserted central catheter (PICC)-line nursing team is consulted and they plan to insert a peripherally inserted catheter for home IV antibiotics for at least the next several days. There is no significant medical history, and he is on no medications. He is alert and participating in care, with both biological parents present who also are engaged appropriately in his care. Consent must be obtained from the parents, but the child should also be engaged in the discussion and "assent" to the procedure.
   a. Complete your "Informed Consent" study/prep card
   b. Be prepared to role-play the role of the intern/physician in obtaining informed consent
   c. Write an Informed Consent Note for the electronic medical record (EMR) AFTER the role play (hint: develop an outline for this case for your use)

2. **Right Heel Laceration: ED-GMO**
   Patient is a 27-year-old female who presents to the ED after dropping a glass on the floor and stepping on the shards, resulting in a 4cm laceration on the bottom of her right heel. He has no other medical problems and has medical decision-making capacity. Probing examination and imaging reveal no residual glass, or neuro/vascular compromise. You and your attending determine that she will require sutures, and you have been tasked with obtaining informed consent for the procedure.
   a. Complete your "Informed Consent" study/prep card
   b. Be prepared to role-play the role of the intern/physician in obtaining informed consent
   c. Write an Informed Consent Note for the electronic medical record (EMR) AFTER the role play (hint: develop an outline for this case for your use)

3. **Blood Transfusion: Anesthesia-Gen Surg**
   A 62-year-old male with significant peripheral arterial disease presents on the morning of a planned, elective right femoral-popliteal bypass. The attending surgeon and anesthesia team agree that the risk for intra-operative blood loss is high and are planning to be prepared to transfuse blood products intraoperatively if necessary. Your attending asks you to send a type and crossmatch, coordinate blood product availability with the blood bank, and obtain informed consent from the patient for a blood transfusion if needed.
   a. Complete your "Informed Consent" study/prep card
   b. Be prepared to role-play the role of the intern/physician in obtaining informed consent
   c. Write an Informed Consent Note for the electronic medical record (EMR) AFTER the role play (hint: develop an outline for this case for your use)

4. **Subcutaneous Mass Removal: Gen Surgery**
   A healthy 23-year-old male presents to the General Surgery clinic for removal of a 2cm subcutaneous soft tissue mass on his upper back. His history, physical exam, and radiographic studies are consistent with benign lipoma. He has no significant medical history and is on no medications. He has no history of substance use. Your senior resident plans to excise the mass - with your assistance - in the minor procedure room in the surgery clinic with local anesthetic and asks you to obtain informed consent for the procedure while the room is being prepped.
   a. Complete your “Informed Consent” study/prep card
   b. Be prepared to role-play the role of the intern/physician in obtaining informed consent
   c. Write an Informed Consent Note for the electronic medical record (EMR) AFTER the role play (hint: develop an outline for this case for your use)

5. **Vasectomy: Fam Med**
   Patient is a 48-year-old male who presents to the family medicine clinic for pre-procedural counseling for a scheduled vasectomy. He has three children with his wife of twenty-two years, ages 17, 13, and 9, and they do not desire additional children. He has no significant past medical history and takes no medications. He had his wisdom teeth removed thirty years ago. He has medical decision-making capacity. You are tasked with obtaining informed consent for the procedure.
   a. Complete your “Informed Consent” study/prep card
   b. Be prepared to role-play the role of the intern/physician in obtaining informed consent
   c. Write an Informed Consent Note for the electronic medical record (EMR) AFTER the role play (hint: develop an outline for this case for your use)

6. **Platelet Rich Plasma (PRP) Injection: Fam Med**
   Patient is a 20-year-old female who presents to the family medicine clinic for a right knee PRP injection. She is a collegiate basketball player with a yearlong history of patellar tendonitis (“jumper’s knee”) that has been refractory to physical therapy and corticosteroid injections. She has no significant past medical history and takes no medications. She had an appendectomy twelve years ago. She has medical decision-making capacity. You are tasked with obtaining informed consent for the procedure.
   a. Complete your “Informed Consent” study/prep card
   b. Be prepared to role-play the role of the intern/physician in obtaining informed consent
   c. Write an Informed Consent Note for the electronic medical record (EMR) AFTER the role play (hint: develop an outline for this case for your use)

7. **Lumbar Puncture: Neuro-ED**
   A 27-year-old female developed the sudden onset of a headache that peaked to a 10/10 intensity over the course of a few seconds while doing cross training today. She developed nausea and vomiting shortly after. Her partner brought her to the ED where she continues to c/o a persistent severe holocranial headache. On exam, she has mild neck stiffness. Her right pupil is 6mm in the light and 6mm in the dark. Her left pupil is 3 mm in the light and 5mm in the dark. You have concern for a SAH and recommend she undergo a lumbar puncture. She has no other significant medical history. She has been engaged in the evaluation of her difficulties and is able to engage in shared decision making.
   a. Complete your “Informed Consent” study/prep card
   b. Be prepared to role-play the role of the intern/physician in obtaining informed consent
   c. Write an Informed Consent Note for the electronic medical record (EMR) AFTER the role play (hint: develop an outline for this case for your use)
