# Supplementary material for: Obtaining and Documenting Informed Consent: An Advanced UME Cross-Specialty, Role-Playing Skill Builder
Source: MedEdPORTAL. 2026 Mar 3;22:11580. doi: 10.15766/mep_2374-8265.11580 (PMC12956033; doi:10.15766/mep_2374-8265.11580)
Supplement: Supplementary file 1 — Course Syllabus.docxPrereadings.pdfStatPearls Article.pdfADMSEP eModule folderClinical Vignettes.pdfRubric.pdfMARRQD, PARRQD Templates.docxOrientation.pptxObserver-Scribe Template.docxVignette Answers.pdf [file mep_2374-8265.11580-s001.zip › F. Rubric.pdf]

A. OBTAINING Informed Consent Guide:

Students are to be provided this template as part of the course pre-work and instructed to review this to prepare their informed consent template.

Use the mnemonic: **PARRQD** or **MARRQD**

Procedure/**M**edication
Alternatives
Risks
Return
Questions
Document

	Procedures (PARRQD)	Medications (MARRQD)	
**Procedure**	Procedure/Intervention Explained<br>Indication/Expected Benefits<br>likelihood of success<br>Contraindications checked<br>Key steps/Expected course<br>Anesthesia, drugs, blood, tubes & lines	Medication Explained<br>Indication/Expected Benefits<br>likelihood of success<br>Contraindications checked<br>Mech of Action<br>Key Steps: Dosage, Expected Course/Duration	**Medication**
**Alternatives**	Alternate Treatments<br>Course WITHOUT procedure	Alternative Treatments<br>Other Meds available<br>Course WITHOUT treatment	**Alternatives**
**Risks**	Common/expected Side Effects<br>(and work-arounds, like stool softeners, etc)<br>"Major" & "Minor" complications<br>Adverse Reactions/Toxicities of accompanying meds<br><br>*Include remote but severe possibilities	Common/expected Side Effects<br>(and work-arounds, like stool softeners, etc)<br>Worrisome Side Effects<br>Adverse Reactions/Toxicities<br><br>*Include remote but severe possibilities	**Risks**
**Return**	Develop a clear side-effect/sx of complication and follow up plan that is understood by the patient	Develop a clear side-effect/sx of complication and follow up plan that is understood by the patient	**Return**
**Questions**	WAIT for some, and also practice "Teach Back"	WAIT for some, and also practice "Teach Back"	**Questions**
**Document**	Surgical/Procedure Preoperative Counseling Note	Medication Counseling Note	**Document**

Remember to ask the patient to "Teach Back": Following the informed consent discussion, confirm complete patient understanding. As a safety precaution, you should check that your patient has understood and agreed to the test, treatment, or procedure right before it is done/prescribed. The teach-back method can help you confirm that your patient understands. Some sample prompts:

What are you having done today?
What does this mediation do/what is it for?
How do you expect to feel afterwards?
What results do you expect? (discuss "good days" vs "more days" and the quality of improvement wanted..and, "How likely is it that you will get those results?
What are the risks? How likely are they to happen?

If a patient cannot answer these questions/participate, STOP THE PROCESS! Halt any activity that could cause harm. Even if all forms have already been signed, and/or prescriptions have been entered/IV access obtained, etc, do not proceed with the test, treatment or procedure, and consider getting more formal capacity assessment completed

B. DOCUMENTING Informed Consent GUIDE:

MEDICATIONS:

The **"Medication Counseling Note"** should capture the essentials …use the "MARRQ" Mnemonic
Be clear, concise, and follow a reliable format, and if family or others were present, include that in your opening.

Medication
Alternatives
Risks
Return
Questions
Documentation

Minute detail is **not** necessary in documentation (despite LOTS of detail when discussing).
Do include that all components of MARR were discussed, and that questions were answered.
Documenting WHAT the "Return/Follow-up" instructions given for side effects/adverse reactions/further questions is necessary too

Medication Informed Consent Rubric from your Psych Clerkship included here to wake up your IC muscles:

- Indication(s): *Why are you recommending this medication for this patient?*
- Mechanism of Action: *Learn mechanism at physician level, and prep words for patients to avoid jargon*
- Benefits: *"This will help reduce the XYZ symptoms you are experiencing."*
- Contraindications: Things to ask about <u>before</u> prescribing: *Do you have liver failure?*
- Duration of Use: *Am I going to be on this forever, doc?*
- Dosing Schedule: *Twice a day? Once a day? As needed?*
- Common/Expected Side Effects and Workarounds: *"Everybody gets an upset tummy but call if XYZ."*
- Black Box Warnings: *Best to hear these from a doctor first, not the Internet.*
- Possible Toxicities/Adverse Drug Reactions (ADRs): *Things worse than expected side effects (especially "special" stuff for this specific med)*
- Alternate treatments/other meds available: *Other meds, ECT, talk therapy?*
- Course of Illness with and without medication: *One month versus one year?*
- Follow-up: *"Let's see you back in 3 days/1 week/2 weeks" (or whenever is appropriate for that med)*
- Emergency Contact: *When/how a patient should contact you or clinic, and what problems to call you for*

PROCEDURES/INTERVENTIONS:

"Trap the plan"—what are you planning to do and why

The **"Surgical/Procedure Preoperative Counseling Note"** should capture the essentials…use the "PARRQD" Mnemonic

Be clear, concise, and follow a reliable format, and if family or others were present, include that in your opening. The order of the PARRQD mnemonic is logical and often how the discussion is presented. However, the order is not critical, that can be tailored to the individual needs of the patient. Making sure to thoroughly discuss all the components is more important than doing so in a particular order.

Procedure, to include Diagnosis & Indication
(or why not offering surgery or patient declined)
Alternatives
Risks
Return
Questions
Document

Minute detail is **not** necessary in documentation (despite LOTS of detail when discussing).

Do include that all components of PARR were discussed, and that all questions were answered.

Documenting WHAT the "Return/Follow-up" instructions that were given for side effects/adverse reactions/further questions is necessary too.

Additional Components for PROCEDURES/INTERVENTIONS (NOT required for 15 Feb…but we want you to know about them):

A. Obtaining a **signed Consent Form** is a SEPARATE, statutorily required item
(not required for 15 FEB exercise; but finding a template for the scenario you are role-playing may help you prepare!)

While documentation policies are often hospital-specific and relate to local and state laws, examples of appropriate documentation and verification practices include the use of reader-friendly consent forms, and chart notes which can be used either as a complement to, or, in some cases, as a substitute for the forms (an example will be included in our didactic introductory talk).

B. Clinic Note and/or admission H&P is ideally separate from the free-standing **"Surgical/Procedure Preoperative Counseling Note**"...and you need BOTH

Record the informed consent discussion in the **clinic note and/or H&P** for the encounter and ideally **reference it again** in a separate.

When you document informed consent, make sure your documentation is complete. If you have

used an interpreter during the informed consent discussion, make a note in the record and have the interpreter sign the informed consent form if one is being used.
Document the choices that you presented to the patient, any decision aids you shared, and your use of teach-back to verify that your patient understood the benefits, harms, and risks of each alternative.

Finally, document the patient's decision, even when patients choose not to have a treatment or procedure.

For both the patient's protection and your own, consider documenting every informed consent discussion in the patient's record, even if it is not required by hospital policy. If a lawsuit is brought against you or the hospital, such documentation could be helpful.

Documentation will also supply critical information to auditors who check to see whether a hospital's informed consent policies are being followed.

C. Procedure/Operative (aka "post-op") **note** written immediately following the procedure (SEPARATE topic for another day). But importantly, must include *did you do what you said you were going to do–and if not–what **DID** you do and why?*

Source: US Agency for Healthcare Research and Quality, the US Department for Health and Human Services, and the Joint Commission, *Making Informed Consent an Informed Choice: Training for Health Care Professionals*.

Example Informed Consent Discussions:

ADMSEP e Module, and below "Thoracentesis Procedure"

Scenario

Adrian Jenson is a 65 year old gentleman who was brought to the hospital eight days ago for lethargy and shortness of breath. He was admitted for hypoxia and soon diagnosed as having right lower lobar pneumonia with a small basilar effusion. Over the last several days the effusion has steadily increased in size and is now a large parapneumonic effusion filling up the lower half of his right pleural space and causing compressive atelectasis on the right lower lobe.

Adrian has been receiving a course of intravenous antibiotics for the past eight days, but his symptoms have continued and have indeed worsened somewhat. The concern is that source control has not been obtained because the fluid in his chest is potentially infected and has not been drained. Furthermore, the effusion could mature into a life-threatening empyema requiring major surgery to evacuate.

Adrian is very tired and stressed out. The doctor visits Adrian on the ward to discuss his choices.

Informed Consent Discussion

Doctor: Mr. Jenson, unfortunately the antibiotic treatment is not likely to be effective if we do not drain the fluid in your chest.

Adrian: I know, the nurse told me that.

Doctor: So we need to talk about what your choices are right now. Is there anyone you'd like to have with you while we discuss this?

[Strategy used: Engaging patients, families, and friends.]

Adrian: Yeah. My sister Ana. She just stepped out to find something to read.

Doctor: It's important that we make some decisions pretty quickly. Can you call her and ask her to come back?

Adrian: Sure.

Doctor: Let's all sit down here and shut the door so we can talk privately.

[Strategy used: prepare for the informed consent discussion and find a private space to talk.]

Doctor: I was just telling your brother that his pneumonia is not getting better because he has developed fluid in his chest that may be infected and is certainly contributing to his symptoms. We need to drain the fluid so that the antibiotics can work so he can get better.

Sister Ana: OK.

Doctor (speaking slowly, with pauses): First let me explain the problem of the chest fluid. The pleural space is a thin space between the outer edges of the lung and of the inside of the chest wall. Normally, there is only a small amount of fluid inside that space. The fluid prevents the lung and chest wall from rubbing together when you breathe.

[“PROCEDURE” from PARRQD mnemonic]

Doctor: Excess fluid in the pleural space is called pleural effusion. When this happens, it’s harder to breathe because the lungs can’t inflate fully. This can cause shortness of breath and pain. We believe your pneumonia is the cause of the effusion, and why you are not getting better.

Doctor: Therefore, we believe you need that fluid removed from your right chest. Because we cannot get the fluid out without a procedure, we could use what’s called an ultrasound-guided thoracentesis to remove the fluid.

Doctor: The ultrasound uses sound waves to show us where the fluid is inside your right chest. The ultrasound is very safe. You may be familiar with ultrasound from previous visits to the doctor. Once we have a good understanding of where the fluid is using the ultrasound, we remove it with the thoracentesis.

Doctor: This is the ultrasound machine [The doctor demonstrates an actual small bedside ultrasound machine if one is available, or alternatively shows a video or a picture of how it is safely used from the outside of the body to safely generate a real-time image of the inside.] Once we see where the fluid is on ultrasound, we then do the thoracentesis procedure to drain it.

Doctor: The thoracentesis is a procedure that involves a needle followed by a thin wire that we use to place a “catheter” which is a small tube we use to drain as much of the fluid from your right chest as possible. The needle and wire are used only at the beginning of the procedure to help get the catheter in and then they are removed. We then use the catheter to get the fluid out. Once the fluid is out, we remove that small tube too. None of these things are left in your body.

Doctor: Removing the fluid will likely help your breathing, and also allows us to run some tests on the fluid to see if it is infected. Let me tell you what the procedure involves in detail.

Doctor: We will do everything we can to make it as painless as possible. We numb up the area with some local anesthetic first. That part will be uncomfortable, but will make the actual procedure much less painful. The numbing medicine is injected with a very small needle, and placed right where we plan to do the thoracentesis. Everything is done in a clean fashion with sterile materials. We will clean your skin with antiseptic solution and place a sterile drape over the area.

Doctor: The thoracentesis itself involves a needle, a wire, and then a tube that we insert into the right chest in the back between your ribs. [The doctor shows one of the many widely available thoracentesis patient education graphics that demonstrate how chest fluid is removed with thoracentesis via seldinger technique.] We then remove the fluid by draining it through the tube and into a bag. We then remove the tube and cover the area with a band aid.

[Strategy used: health literacy universal precautions: use plain, nonmedical language. Speak slowly and use visual aids.]

Doctor: Here is what we would do specifically during the thoracentesis part. We would have you sit up on the bed, with your arms resting on a table. That position spreads out the space between your ribs. The area where the needle will be inserted will be cleaned and numbed up with a local anesthetic. We will insert the needle between the ribs in your right back, the wire will be placed through the needle, the needle removed. The tube will be placed over the wire and fluid will be withdrawn. We will be asking you to be still, exhale, and hold his breath at different times. Once enough fluid has been drained, the needle will be removed and the area will be covered with a band-aid. The spot where the needle went in will close without any stitches. This normally is a 15-minute procedure. If there is a lot of fluid to remove, it may take longer.

Doctor: We hope and believe this procedure will help you breathe better, allow your antibiotics to work, and potentially avoid further problems where the fluid becomes so infected that you would need surgery to have it removed. This is all pretty technical, and I want to make sure I explained that properly. Can you tell me what you understood about what the pleurocentesis procedure is and why we'd do one?

[Strategy used: Use "teach-back," giving the information in "chunks," and checking for comprehension.]

Adrian: You use an ultrasound to find the fluid in my right chest. Then you numb the area up and use a needle and a tube to remove the fluid you saw on the ultrasound. You test the fluid to see if it is infected. And you want to do this to help my breathing and allow my antibiotics to treat my pneumonia so I can get better.

Doctor: Right. And potentially avoid surgery. If we don't get the fluid out, the fluid could become infected and the situation will only get worse. Infected fluid in the chest can develop into what is called an empyema and it can be life threatening. It could become essentially a collection of "pus" or an "abscess" underneath your lung.

Adrian: I sure don't want that.

["RISKS" from PARRQD mnemonic]

Doctor: Now, I have to tell you that there are risks in doing a thoracentesis procedure. Every procedure has some potential problems. Though thoracentesis is generally considered safe, complications can happen. [Doctor explains in layman's terms the potential risks, their probabilities ideally at the doctor's own institution or better yet the doctor's own personal numbers if available, and what the contingencies would be; such as pneumothorax or "collapsed lung" potentially requiring a separate additional procedure called a "chest tube"; reexpansion pulmonary edema or "fluid inside the lungs"; bleeding potentially requiring the need for blood products or surgery; etc.]

["RETURN" from PARRQD mnemonic]

Doctor: If everything goes well, here is what happens after the procedure. While fluid samples are sent off for examination, a nurse will closely watch your blood pressure, pulse, and breathing. We will get a chest x-ray to see what your lungs look like with the fluid removed and to check for any complications.

We will check your band-aid to make sure there are no problems at the needle site. You will be here in the hospital with us so we will be able to watch you closely. Please let us know if you experience any new or concerning symptoms of worsening bleeding, pain, fever, chills, or light-headedness. Once you recover from your pneumonia and are well enough to go home, we will also check the site in the clinic two weeks after you are discharged from the hospital. The time and location of that appointment will be in your discharge paperwork and we will give you a number to call if you have any problems once you get home.

[Strategy used: Explain benefits, harms, and risks of all options, and offer information in more than one way]

Adrian (sounding scared): Can't we just hold off on this for now and see if it starts to get better in one or two more days?

["ALTERNATIVES" from PARRQD mnemonic]

Doctor: You absolutely could choose to do that.

[Strategy used: Offer choices; affirm that it's the patient's choice.]

Doctor: But I have to tell you, there are risks of not doing this procedure. As I said, without draining the fluid your shortness of breath will probably not improve soon and could get much worse. We will not know whether the fluid is infected or not. We believe your antibiotics will not work as well. The fluid could consolidate into an infected "empyema" that could be life-threatening and require surgery. That is the "abscess" in your chest I talked about. I wish I could be more precise about what the odds are, but there's just a lot of uncertainty.

[Strategy used: Explain benefits, harms, and risks of all options, including the option of no treatment.]

Adrian: Wow. That's really scary.

Doctor: I know. And I'm not sure I explained it that well. Can you tell me what you think will happen if we wait to do this procedure or do not do it at all?

Adrian: Sounds like there's a chance I will die.

Doctor: Well, there is a chance of that if it develops into a full-blown empyema. It could certainly become a life-threatening problem that might need major chest surgery. But we don't know exactly how likely that is to happen if we were to wait one or two days. But the chances are greater that you will worsen if we wait than if we proceed with the procedure today. So let's see if I explained that more clearly. Can you tell me what you think will happen if we wait too long to drain this fluid?

[Strategy used: Use teach-back (re-teach using different words, re-check after re-teaching)]

Adrian(a little uncertain): I am more likely to worsen if I wait or skip this procedure altogether rather than doing this procedure today. And if I do wait and end up getting an abscess in my chest, that could possibly involve a big surgery and could even put my life at risk. Is that right?

Doctor: That's right. And what about if we do the thoracentesis today? What might happen then?

Adrian: It may well make my breathing better today, help my antibiotics work, and hopefully prevent me from getting even more sick. Plus I could potentially avoid a big surgery.

Doctor: Yes. And what else could happen?

Adrian: If I don't do the procedure and get sicker this infection could even threaten my life.

Doctor: Yes…that could unfortunately happen. But letting pneumonia with a large effusion go undrained is a lot riskier than a thoracentesis. I've given you a lot of information and you've got a hard decision to make. Now I'm sure you have some questions you'd like to ask me. What more can I tell you?

["QUESTIONS" from PARRQD mnemonic]

Adrian: Do you think the fluid is already infected?

Doctor: It's hard to say. As you know, we are not able to find out if the fluid is infected until we remove it and do the tests to find out. The fact that your antibiotics are not working as well as we hoped suggests it may indeed be infected. And if not, it could become so. These are all good reasons to get the fluid out. Ultimately we won't know for sure until we get a sample of the fluid and test it.

[Strategy used: Explain the benefits, harms and risks of all options; acknowledge uncertainty.]

Doctor: What else are you concerned about?

[Strategy used: Encourage more questions.]

Sister Ana: What are the chances Adrian will die from a complication from the procedure?

Doctor: The chances of his dying from a thoracentesis is very small—well less than one person out of 1,000 who gets a thoracentesis dies from the procedure. What other questions do you have?

[Strategy used: Engage patients, friends and families; encourage questions.]

Adrian: I can't think of any more. My poor Adrian! He's been through so much already.

Doctor: Why don't you take a little time to think about it and talk with your sister?

Adrian: Yes…I need time to think.

Doctor: Of course. Then I'll leave you with these notes and let you two talk. I'll come back in an hour. And if you have any more questions before then, have the nurse page me. How does that sound?

Adrian: That sounds good.

[Strategy used: schedule multiple sessions for the informed consent discussion if needed.]

One hour later…

Doctor: Hi, Mr. Jenson. Have you had a chance to think about the thoracentesis?

Adrian: Yes, but I'm still having a hard time deciding.

Doctor: OK. It's a tough decision. When you think about making this decision, what are you thinking about? What are your main concerns?

[Strategy used: Elicit goals and values.]

Adrian: I just want to go home. I want to get out of this hospital and get back to a normal life. Being in the hospital is miserable. I want to get home and see my grandkids. There is a bunch of stuff piling up that I need to get done at the house.

Doctor: I hear how much you'd like to go home and get back to the rest of your family. It can be so hard being pulled in different directions.

[Strategy used: Engage patients, families and friends by showing respect, listening, and using a caring tone.]

Adrian: Yes, Doctor, I sure am.

Sister Ana: You know, I can check on the house, pick up the mail, and take care of whatever you need me to do there. And I can update the family. I know they can help too.

Doctor: That's great. So, Mr. Jenson, now that you've heard what your sister had to say, what are you most worried about?

[Strategy used: Help patients choose.]

Adrian: I am worried about this getting worse and I sure want to avoid surgery if at all possible. But I'm also worried that there might be a complication from this procedure today.

Doctor: That could happen. But if you're just comparing risks, having a potentially infected fluid in your chest is a lot riskier than having a thoracentesis.

Adrian: Well it sure sounds like that perhaps the fastest way to get better and get back home is to do the thoracentesis, and do it today. OK. Let's do the thoracentesis.

["DOCUMENT" from PARRQD mnemonic]

Essentials here include:

1. A clear, concise, statutorily correct consent form is reviewed, understood, and signed by all parties.
2. The informed consent discussion is documented in the patient's record in the daily progress note.
3. The informed consent discussion is captured in a separate pre-procedure counseling note documenting all the essential elements of the detailed informed consent discussion that actually took place.
4. After the thoracentesis is performed, a post-procedure note is placed in the record describing the conduct of the procedure, the findings, any complications, and its early outcome.

Adapted from: US Agency for Healthcare Research and Quality, the US Department for Health and Human Services, and the Joint Commission, *Making Informed Consent an Informed Choice: Training for Health Care Professionals*.
