# Supplementary material for: Obtaining and Documenting Informed Consent: An Advanced UME Cross-Specialty, Role-Playing Skill Builder
Source: MedEdPORTAL. 2026 Mar 3;22:11580. doi: 10.15766/mep_2374-8265.11580 (PMC12956033; doi:10.15766/mep_2374-8265.11580)
Supplement: Supplementary file 1 — Course Syllabus.docxPrereadings.pdfStatPearls Article.pdfADMSEP eModule folderClinical Vignettes.pdfRubric.pdfMARRQD, PARRQD Templates.docxOrientation.pptxObserver-Scribe Template.docxVignette Answers.pdf [file mep_2374-8265.11580-s001.zip › G. MARRQD, PARRQD Templates.docx]

Students are to be provided the following templates as part of the course pre-work and instructed to complete the appropriate template for their assigned medication or procedure vignette prior to the synchronous session.

|  | **Procedure (PARRQD)** |
| --- | --- |
| **Procedure** | **Procedure/Intervention: (general description appropriate for patients (think 8^th^ grade education)**  **Indication/Expected Benefits/likelihood of success**  **Contraindications**  **Key steps/Expected course**  Anesthesia, drugs, blood, tubes & lines, recovery, rehab, nursing care, etc |
| **Alternatives** | **Alternate Treatments**  **Course WITHOUT procedure** |
| **Risks** | **Common/expected Side Effects (and work-arounds, like stool softeners, etc)**  **“Major” & “Minor” complications**  **Adverse Reactions/Toxicities of accompanying meds**  *Include remote but severe possibilities |
| **Return** | **Things for patient to watch for:**  **Things for patient to return for:**  **Standard/expected follow up plan:** |
| **Document** | **Surgical/Procedure Preoperative Counseling Note**  (a short, effective, appropriate EHR/EMR counselling note in this box) |

** Remember to allow questions from the patient

| **Medications (MARRQD)** |  |
| --- | --- |
| **Medication: (general description appropriate for patients (think 8th grade education)**  **Indication/Expected Benefits/likelihood of success**  **Contraindications**  **Mech of Action**  **Key Steps: Dosage, Expected Course/Duration** | **Medication** |
| **Alternate Treatments**  **Course WITHOUT treatment** | **Alternatives** |
| **Common/expected Side Effects (and work-arounds, like stool softeners, etc)**  **“Major” and “Minor” Side Effects/complications**  **Adverse Reactions/Toxicities**  *Include remote but severe possibilities | **Risks** |
| **Things for patient to watch for:**  **Things for patient to return for:**  **Standard/expected follow-up plan:** | **Return** |
| **Medication Counseling Note** (a short, effective, appropriate EHR/EMR counselling note in this box) | **Document** |

** Remember to allow questions from the patient
