# Supplementary material for: Obtaining and Documenting Informed Consent: An Advanced UME Cross-Specialty, Role-Playing Skill Builder
Source: MedEdPORTAL. 2026 Mar 3;22:11580. doi: 10.15766/mep_2374-8265.11580 (PMC12956033; doi:10.15766/mep_2374-8265.11580)
Supplement: Supplementary file 1 — Course Syllabus.docxPrereadings.pdfStatPearls Article.pdfADMSEP eModule folderClinical Vignettes.pdfRubric.pdfMARRQD, PARRQD Templates.docxOrientation.pptxObserver-Scribe Template.docxVignette Answers.pdf [file mep_2374-8265.11580-s001.zip › H. Orientation.pptx]

## Slide 1
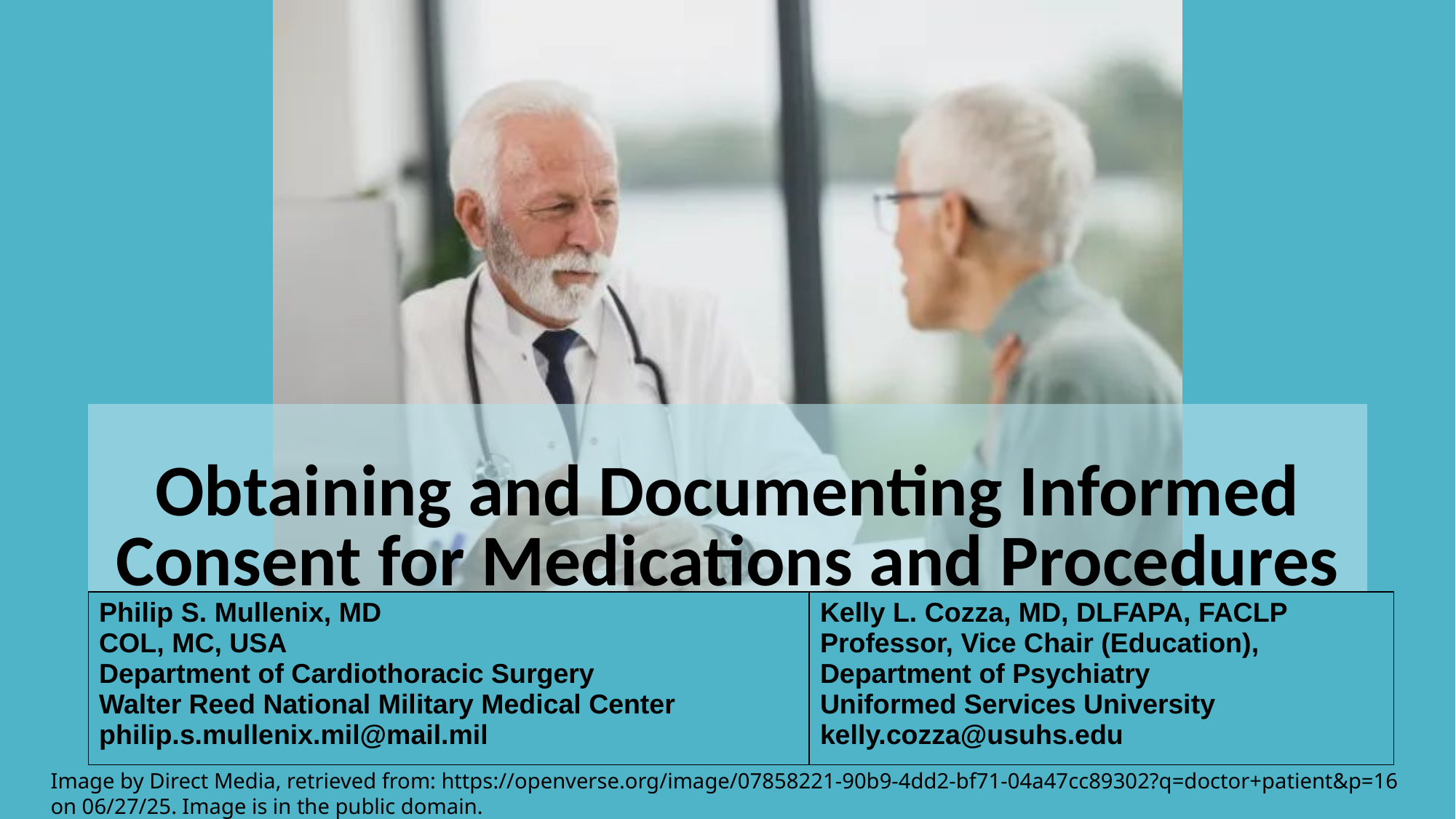

# Obtaining and Documenting Informed Consent for Medications and Procedures
| Philip S. Mullenix, MD COL, MC, USA Department of Cardiothoracic Surgery Walter Reed National Military Medical Center philip.s.mullenix.mil@mail.mil | Kelly L. Cozza, MD, DLFAPA, FACLP Professor, Vice Chair (Education), Department of Psychiatry Uniformed Services University kelly.cozza@usuhs.edu |
| --- | --- |
Image by Direct Media, retrieved from: https://openverse.org/image/07858221-90b9-4dd2-bf71-04a47cc89302?q=doctor+patient&p=16 on 06/27/25. Image is in the public domain.

## Slide 2
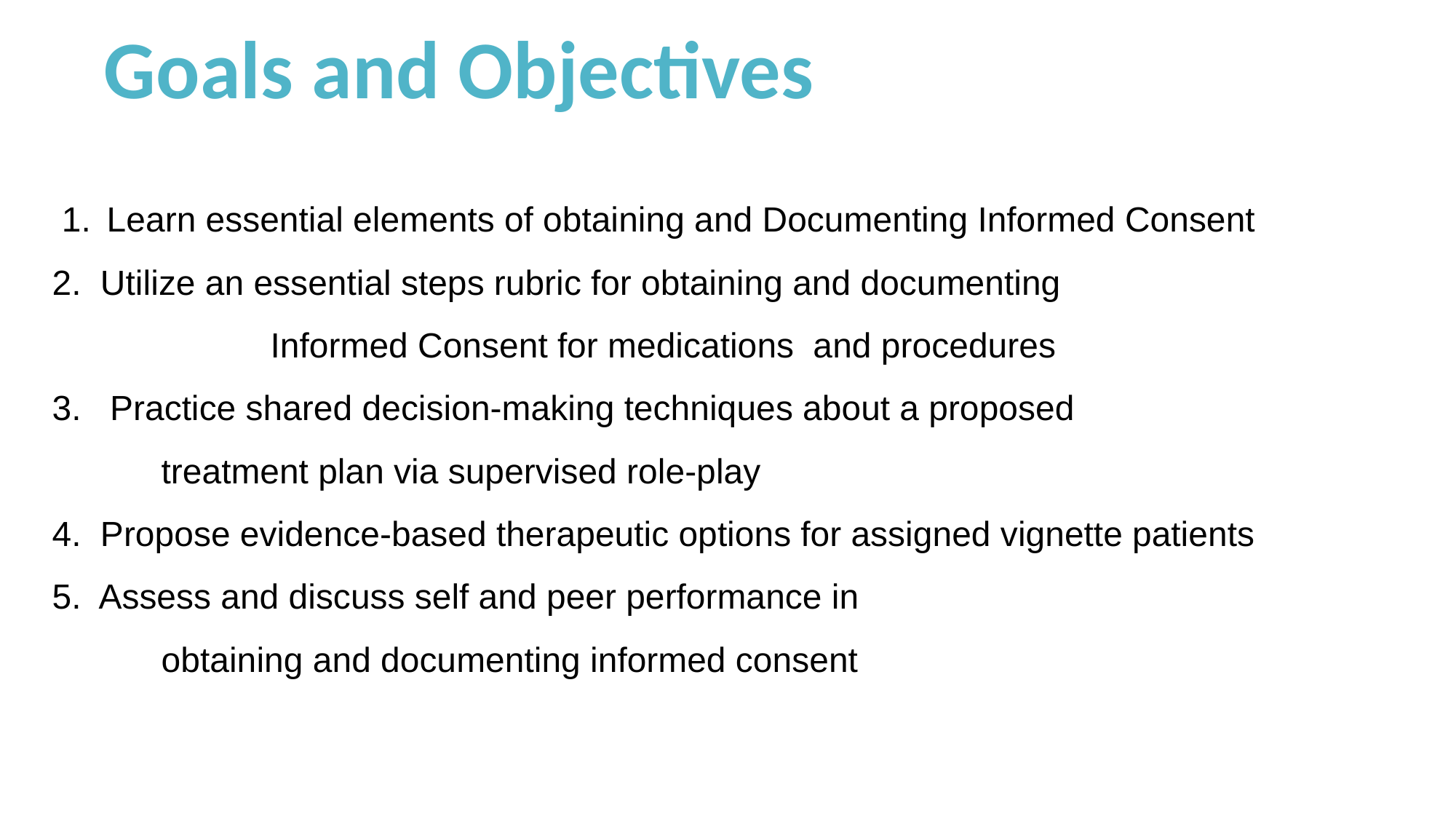

# Goals and Objectives
Learn essential elements of obtaining and Documenting Informed Consent
2. Utilize an essential steps rubric for obtaining and documenting
 		Informed Consent for medications and procedures
3. Practice shared decision-making techniques about a proposed
treatment plan via supervised role-play
4. Propose evidence-based therapeutic options for assigned vignette patients
5. Assess and discuss self and peer performance in
obtaining and documenting informed consent

## Slide 3
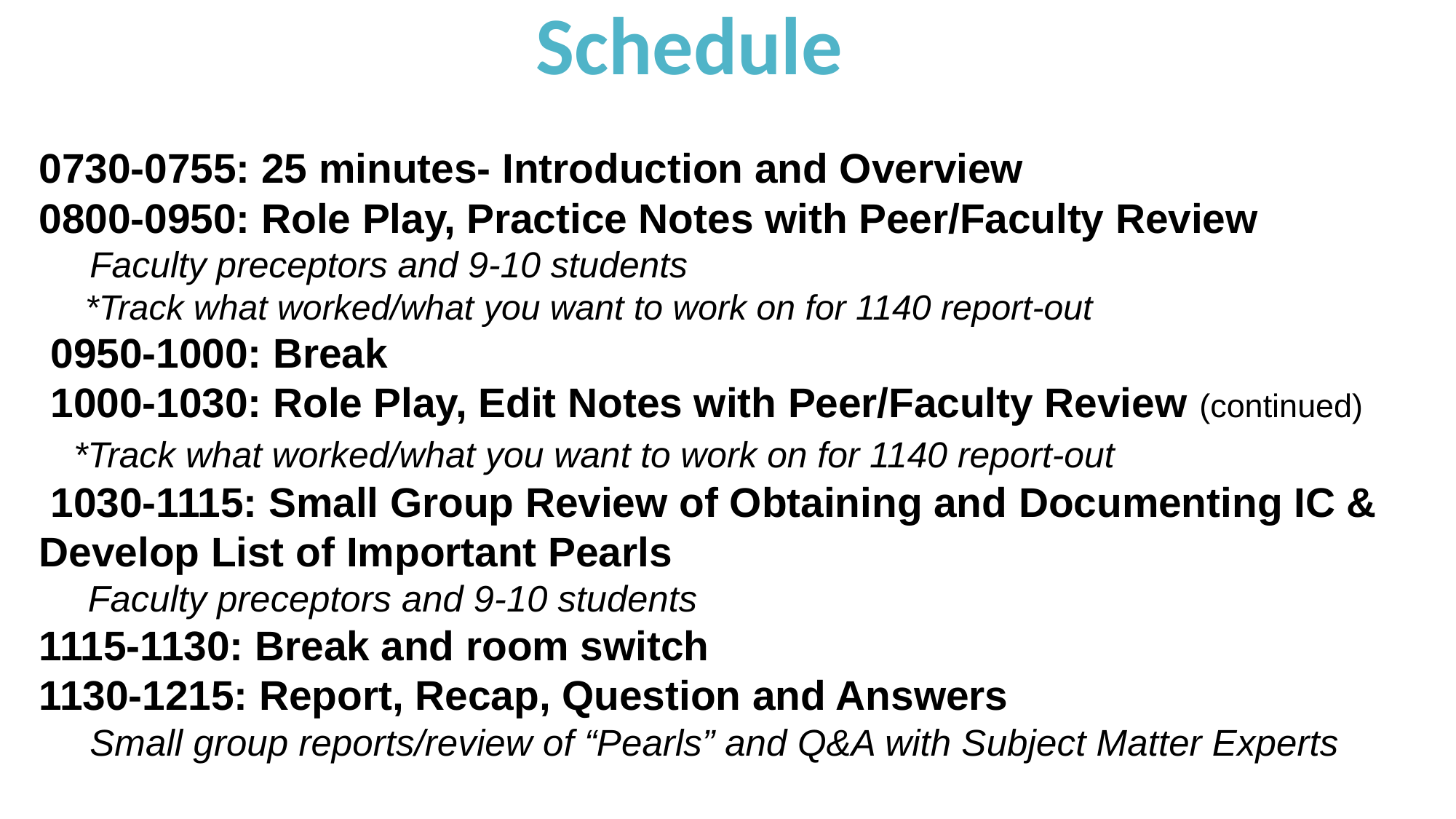

# Schedule
0730-0755: 25 minutes- Introduction and Overview
0800-0950: Role Play, Practice Notes with Peer/Faculty Review
Faculty preceptors and 9-10 students
 *Track what worked/what you want to work on for 1140 report-out
 0950-1000: Break
 1000-1030: Role Play, Edit Notes with Peer/Faculty Review (continued)
 *Track what worked/what you want to work on for 1140 report-out
 1030-1115: Small Group Review of Obtaining and Documenting IC & Develop List of Important Pearls
 Faculty preceptors and 9-10 students
1115-1130: Break and room switch
1130-1215: Report, Recap, Question and Answers
Small group reports/review of “Pearls” and Q&A with Subject Matter Experts

## Slide 4
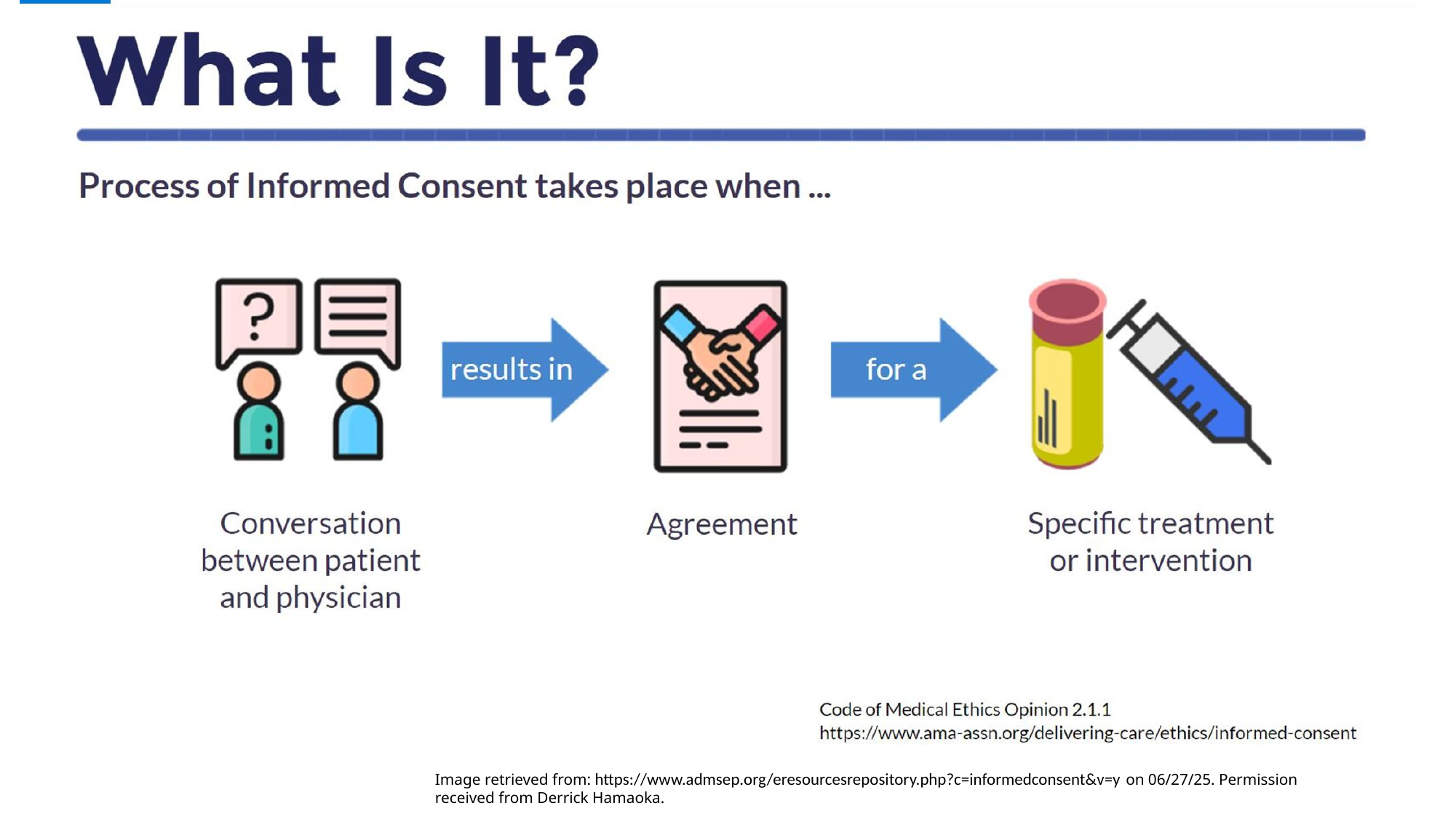

Image retrieved from: https://www.admsep.org/eresourcesrepository.php?c=informedconsent&v=y on 06/27/25. Permission received from Derrick Hamaoka.

## Slide 5
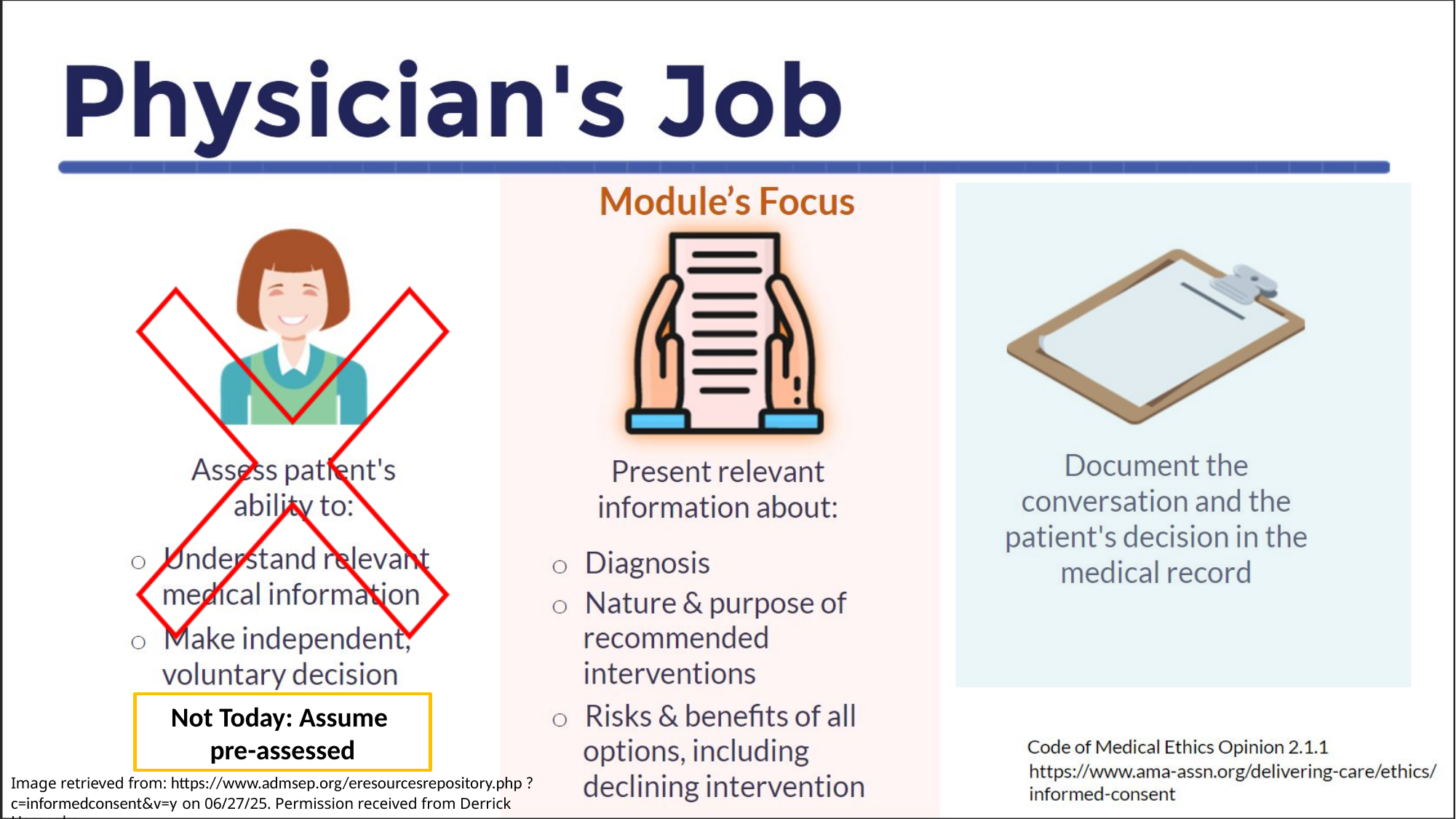

Not Today: Assume
pre-assessed
Image retrieved from: https://www.admsep.org/eresourcesrepository.php ?c=informedconsent&v=y on 06/27/25. Permission received from Derrick Hamaoka.

## Slide 6
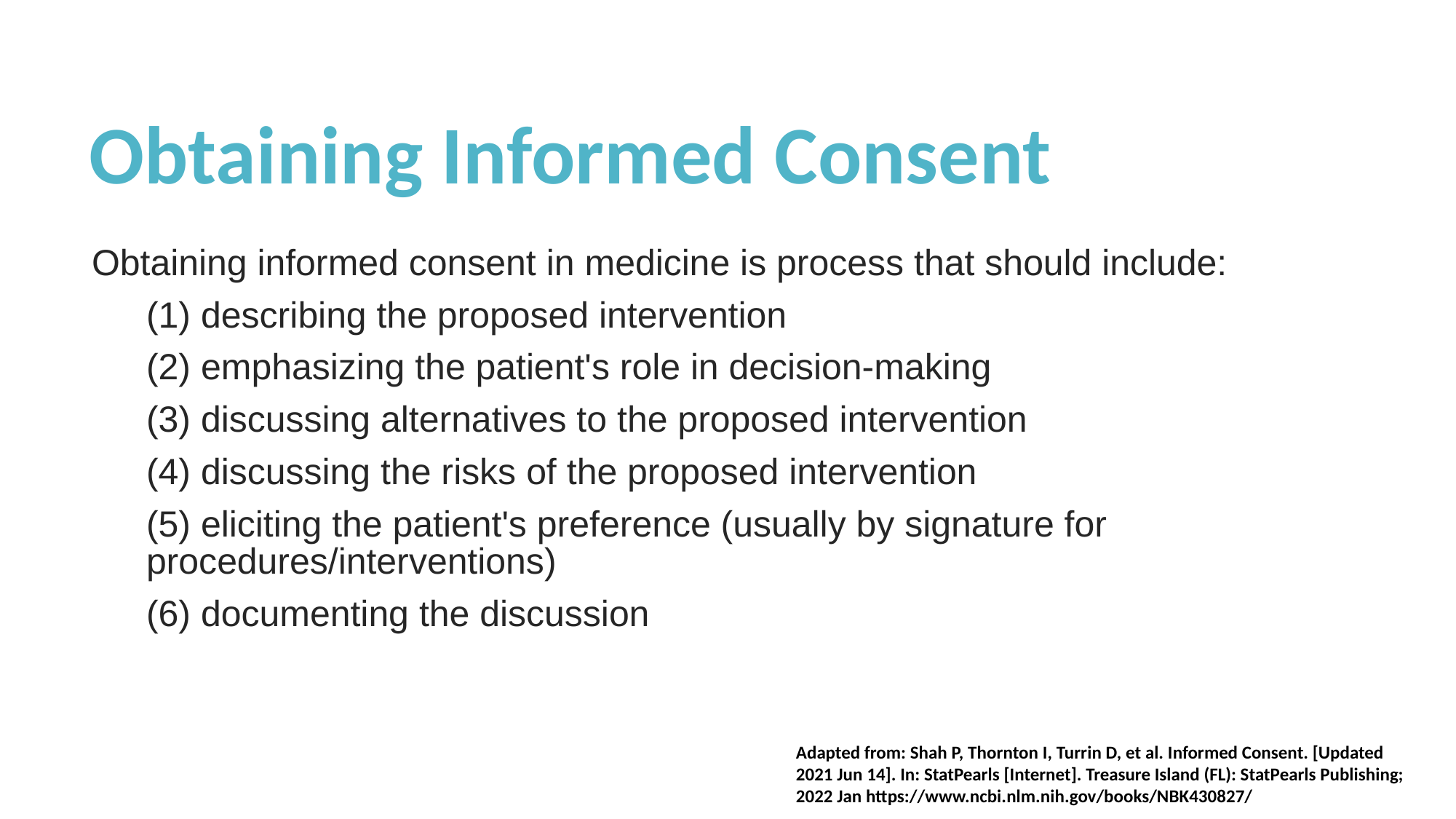

# Obtaining Informed Consent
Obtaining informed consent in medicine is process that should include:
(1) describing the proposed intervention
(2) emphasizing the patient's role in decision-making
(3) discussing alternatives to the proposed intervention
(4) discussing the risks of the proposed intervention
(5) eliciting the patient's preference (usually by signature for procedures/interventions)
(6) documenting the discussion
Adapted from: Shah P, Thornton I, Turrin D, et al. Informed Consent. [Updated 2021 Jun 14]. In: StatPearls [Internet]. Treasure Island (FL): StatPearls Publishing; 2022 Jan https://www.ncbi.nlm.nih.gov/books/NBK430827/

## Slide 7
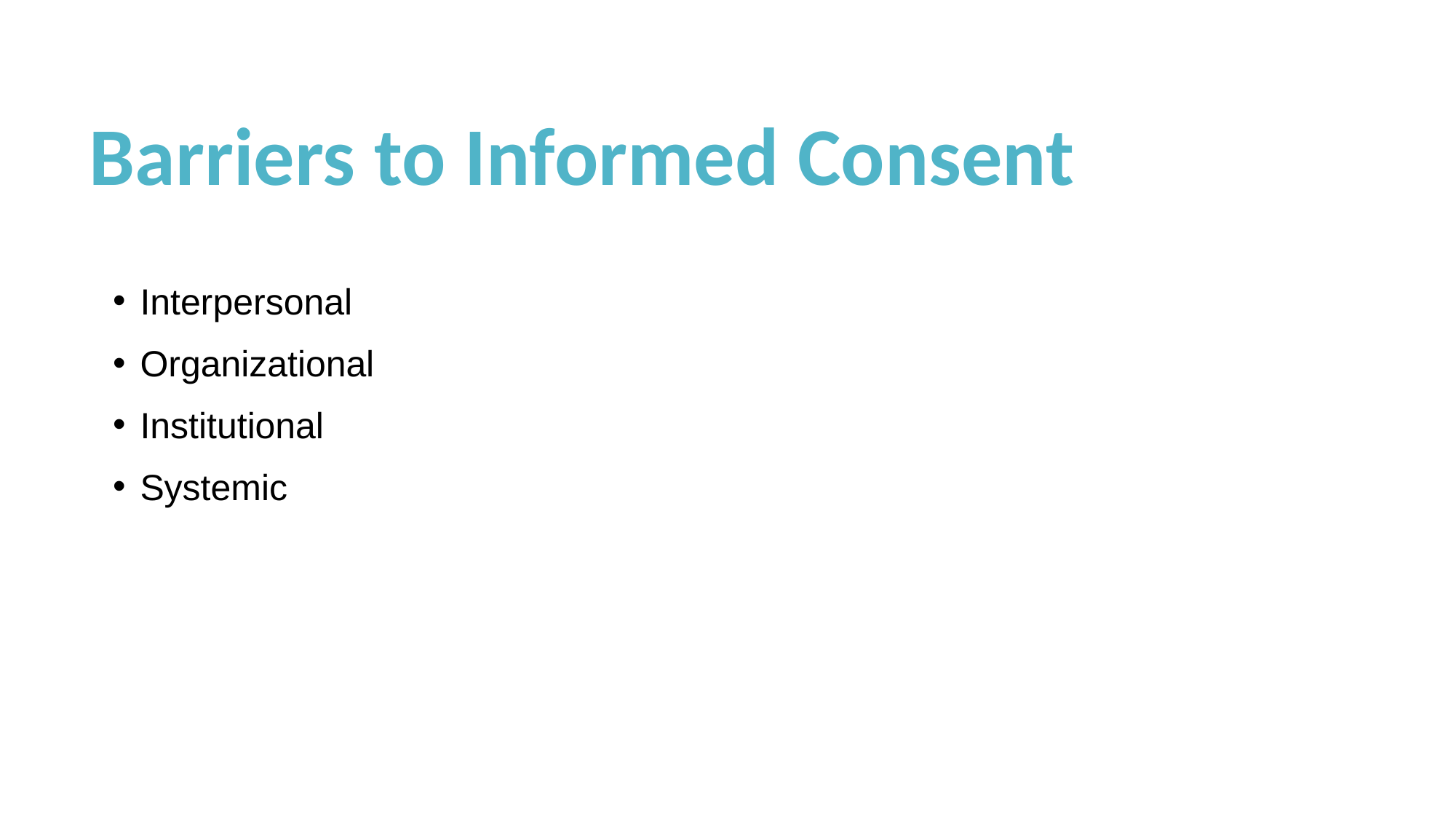

# Barriers to Informed Consent
Interpersonal
Organizational
Institutional
Systemic

## Slide 8
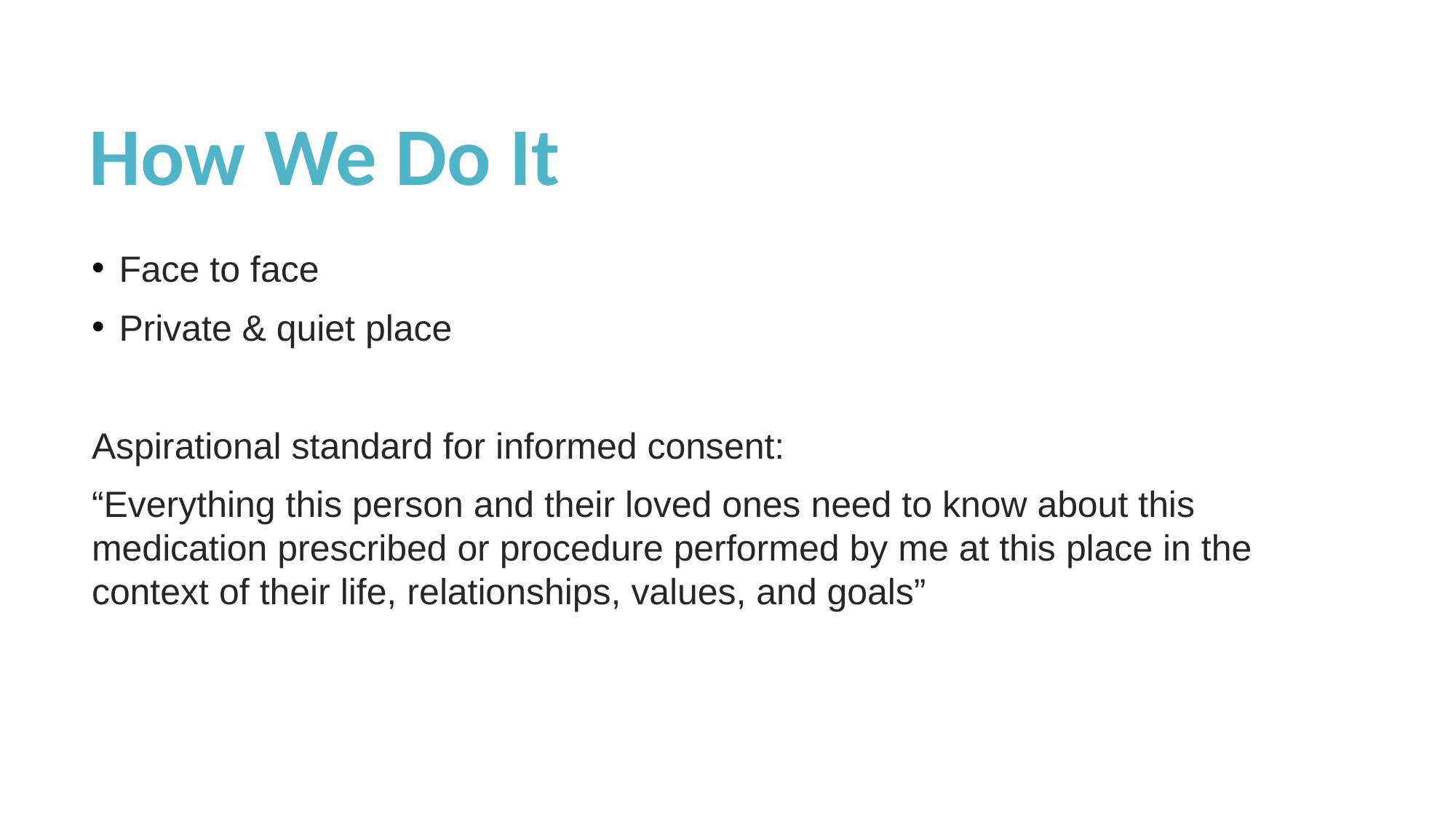

# How We Do It
Face to face
Private & quiet place
Aspirational standard for informed consent:
“Everything this person and their loved ones need to know about this medication prescribed or procedure performed by me at this place in the context of their life, relationships, values, and goals”

## Slide 9
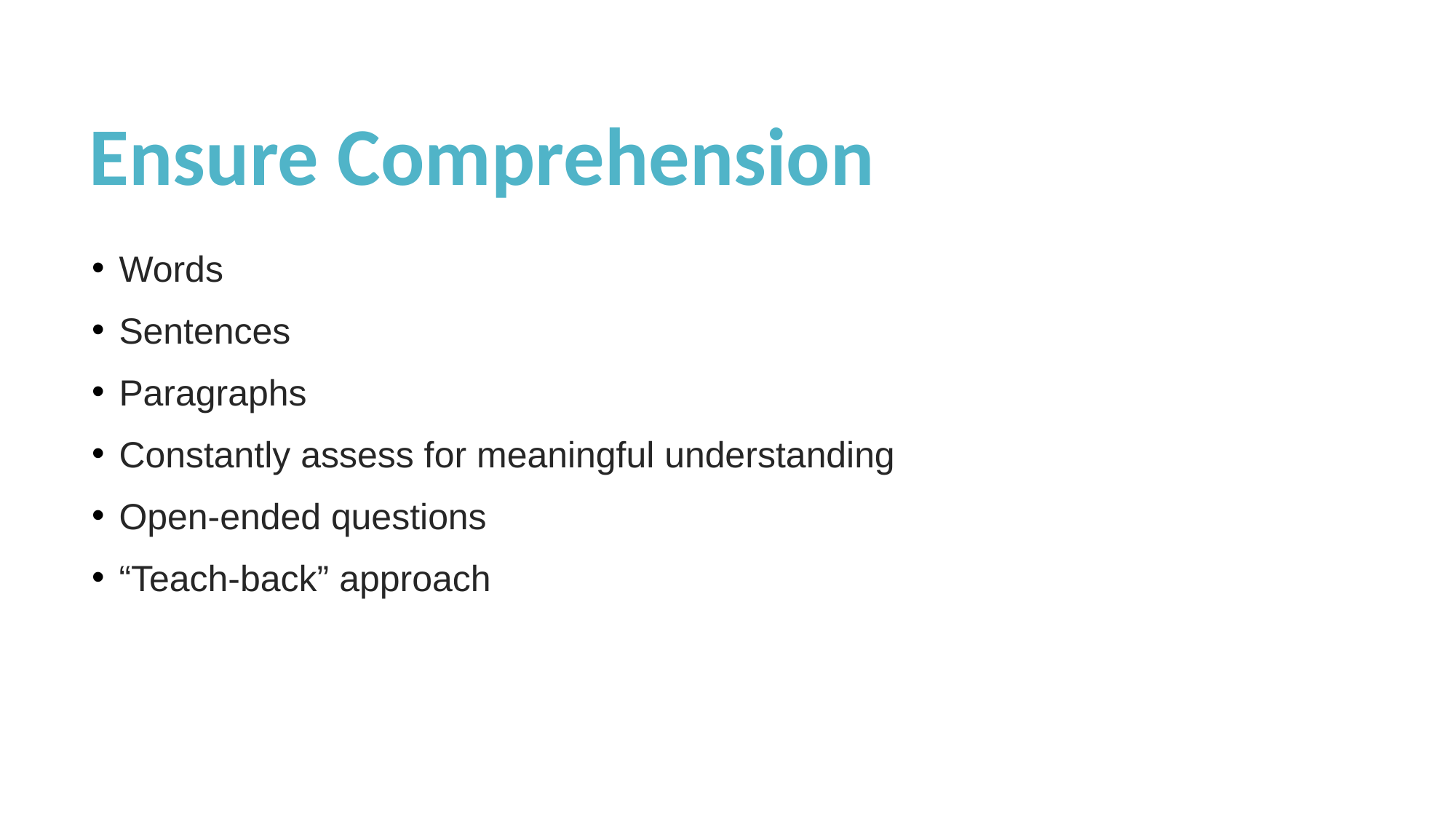

# Ensure Comprehension
Words
Sentences
Paragraphs
Constantly assess for meaningful understanding
Open-ended questions
“Teach-back” approach

## Slide 10
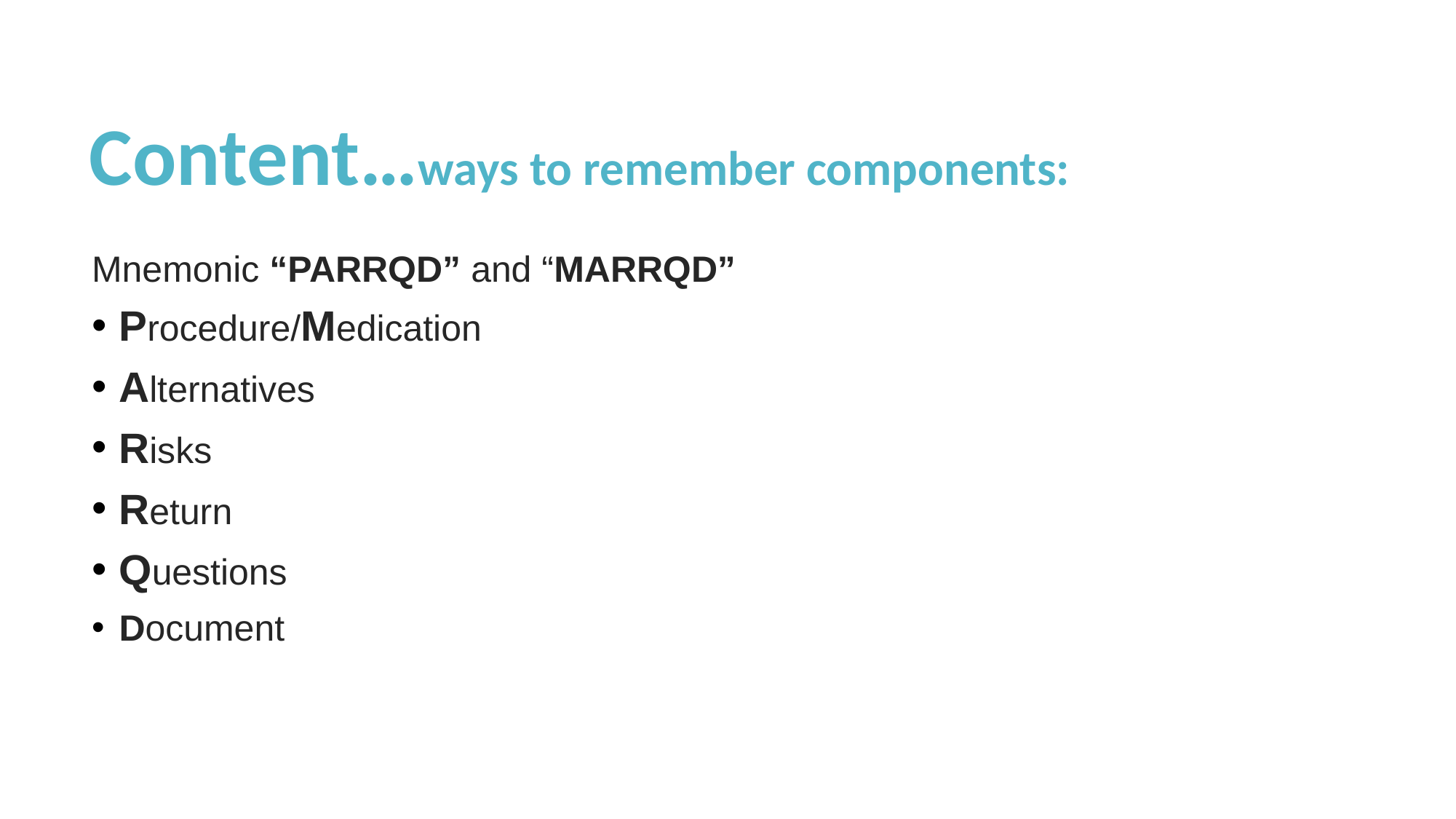

# Content…ways to remember components:
Mnemonic “PARRQD” and “MARRQD”
Procedure/Medication
Alternatives
Risks
Return
Questions
Document

## Slide 11
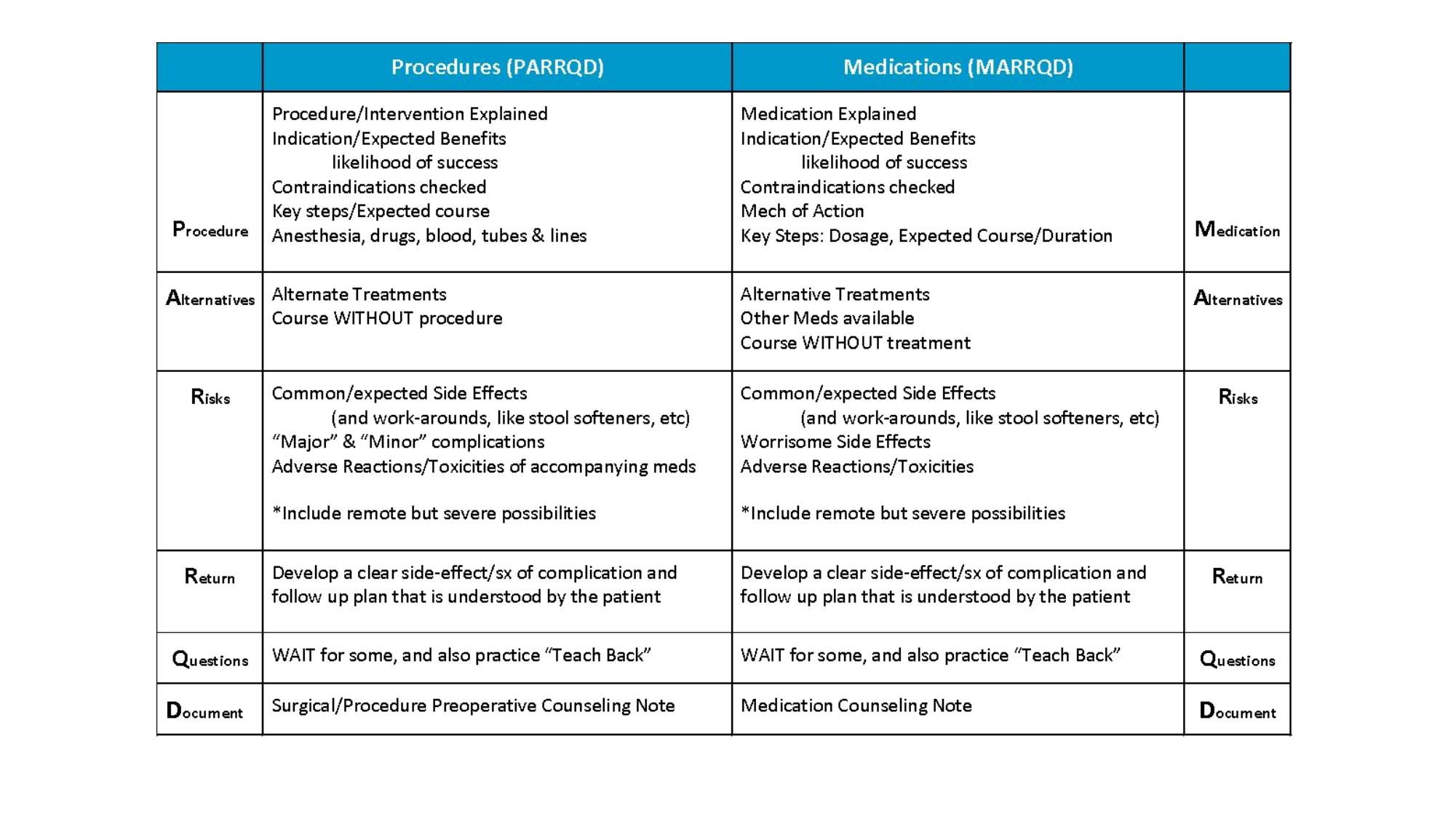

## Slide 12
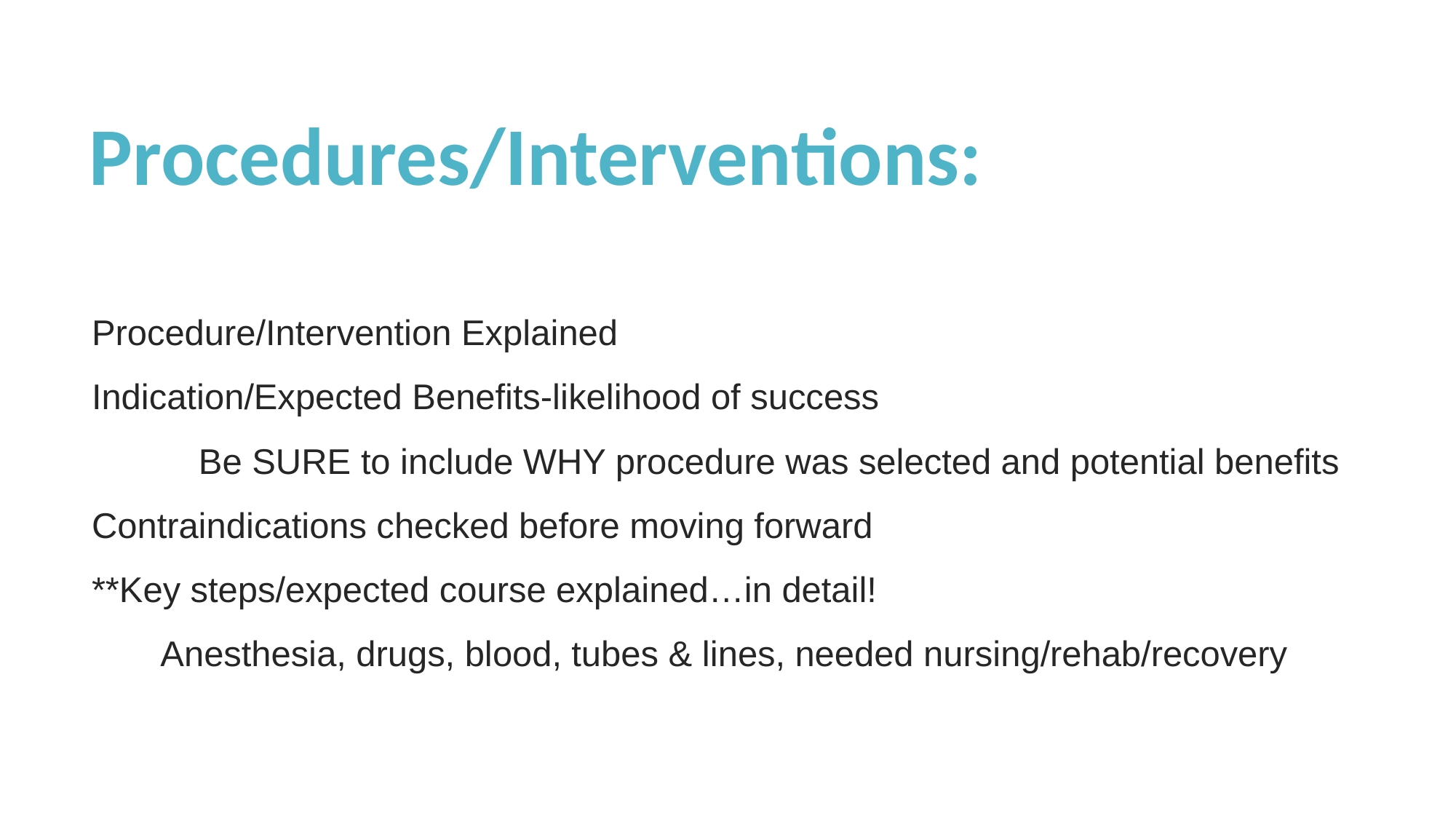

# Procedures/Interventions:
Procedure/Intervention Explained
Indication/Expected Benefits-likelihood of success
	Be SURE to include WHY procedure was selected and potential benefits
Contraindications checked before moving forward
**Key steps/expected course explained…in detail!
 Anesthesia, drugs, blood, tubes & lines, needed nursing/rehab/recovery

## Slide 13
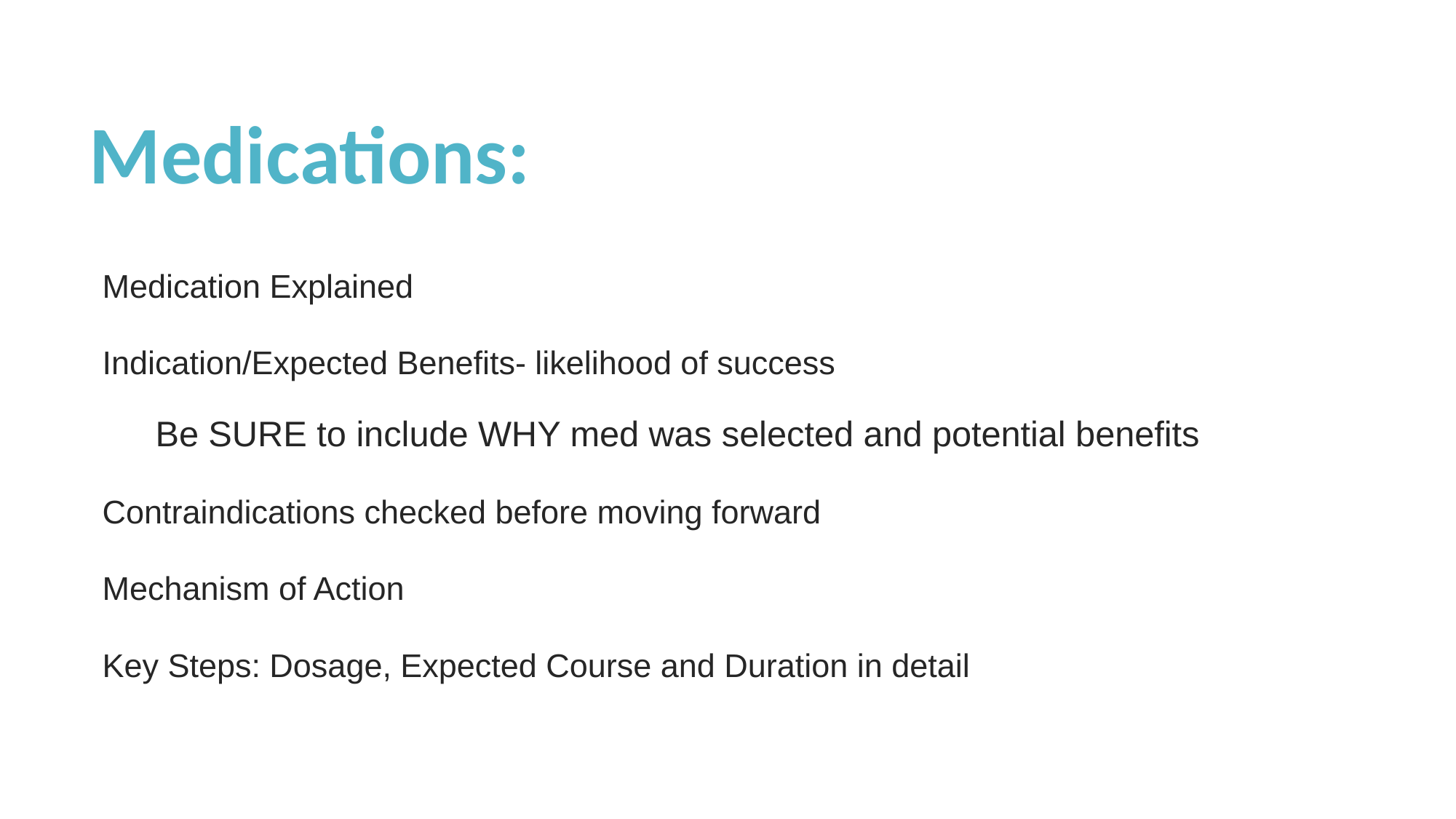

# Medications:
Medication Explained
Indication/Expected Benefits- likelihood of success
Be SURE to include WHY med was selected and potential benefits
Contraindications checked before moving forward
Mechanism of Action
Key Steps: Dosage, Expected Course and Duration in detail

## Slide 14
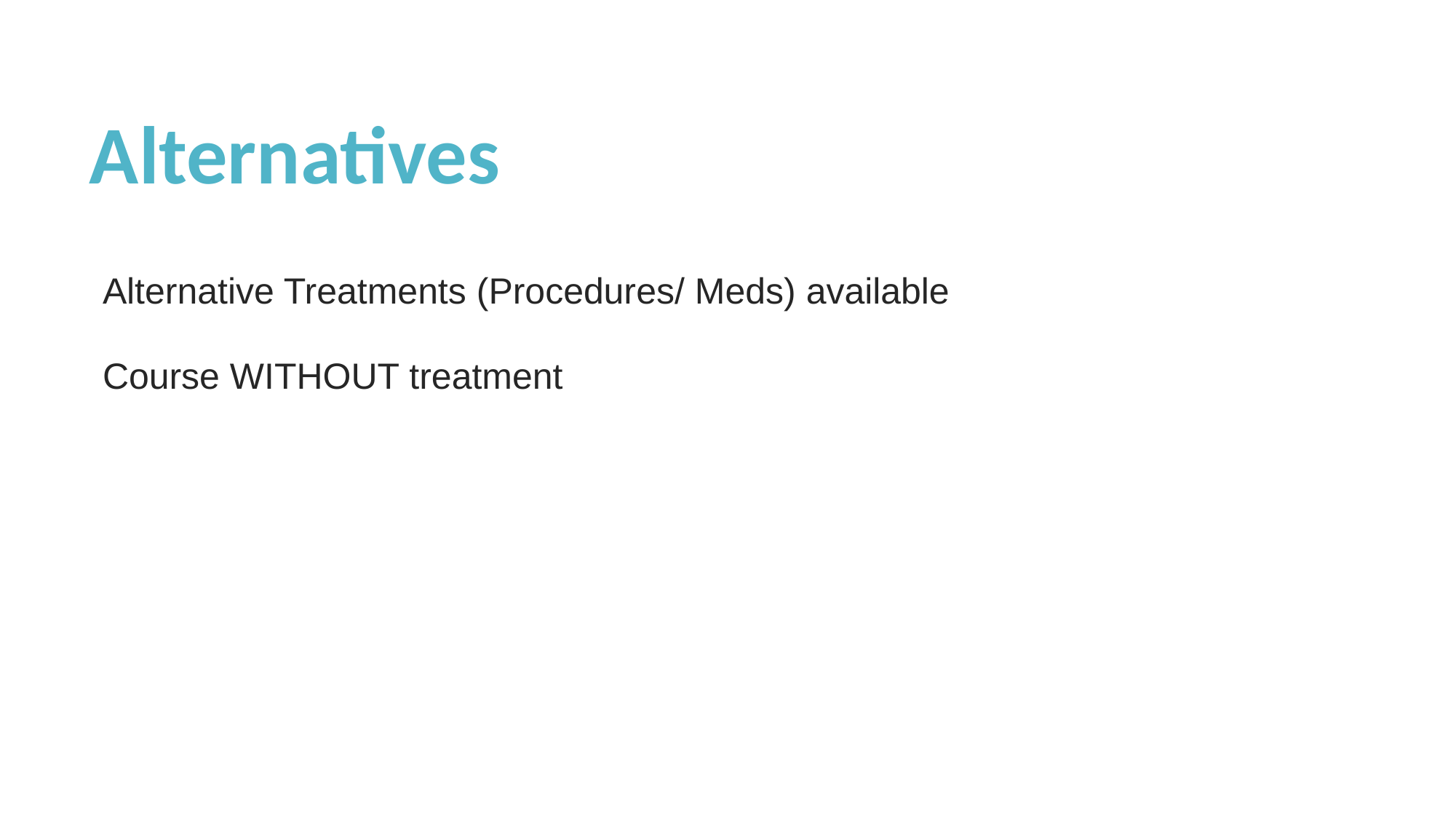

# Alternatives
Alternative Treatments (Procedures/ Meds) available
Course WITHOUT treatment

## Slide 15
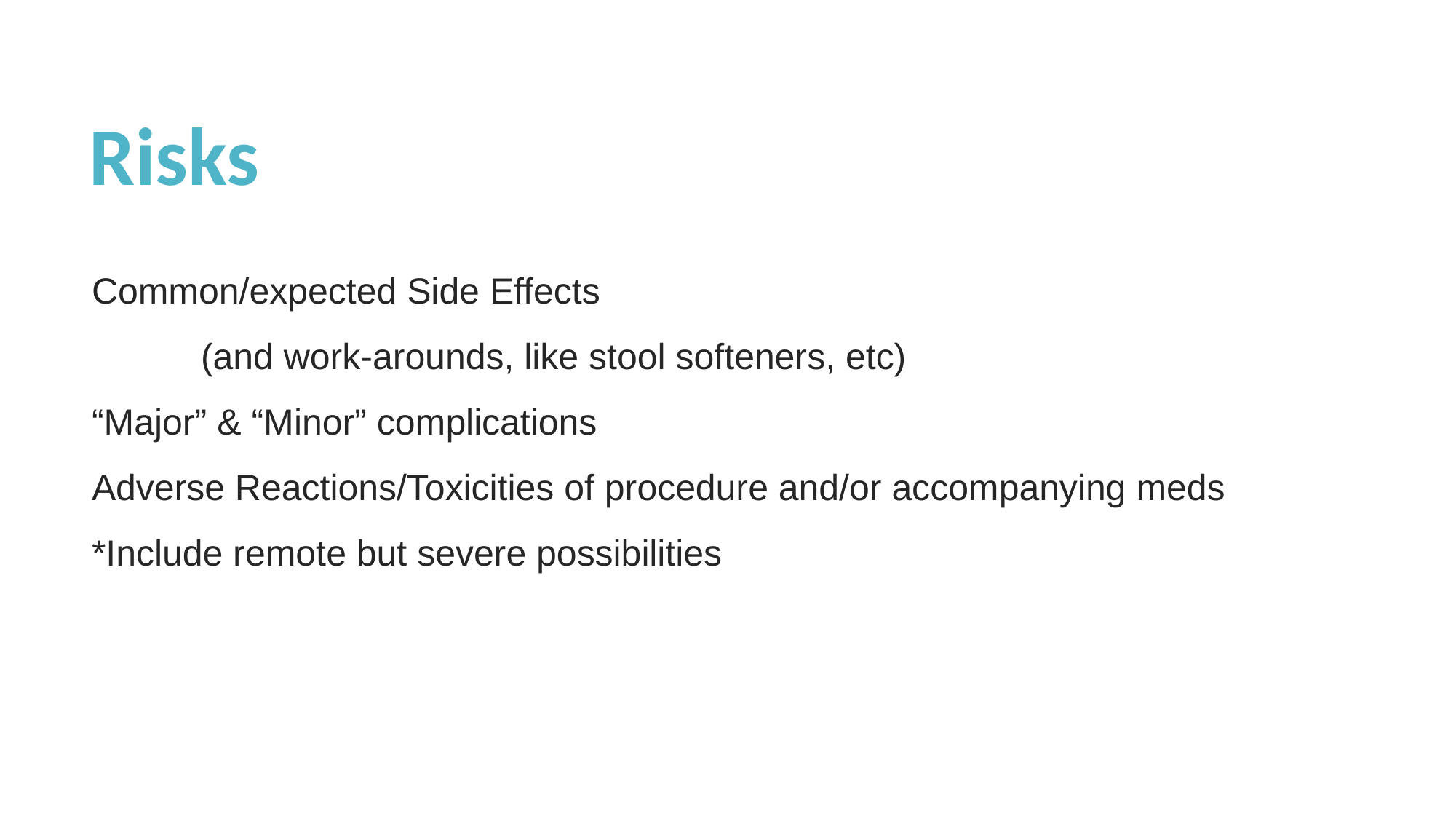

# Risks
Common/expected Side Effects
	(and work-arounds, like stool softeners, etc)
“Major” & “Minor” complications
Adverse Reactions/Toxicities of procedure and/or accompanying meds
*Include remote but severe possibilities

## Slide 16
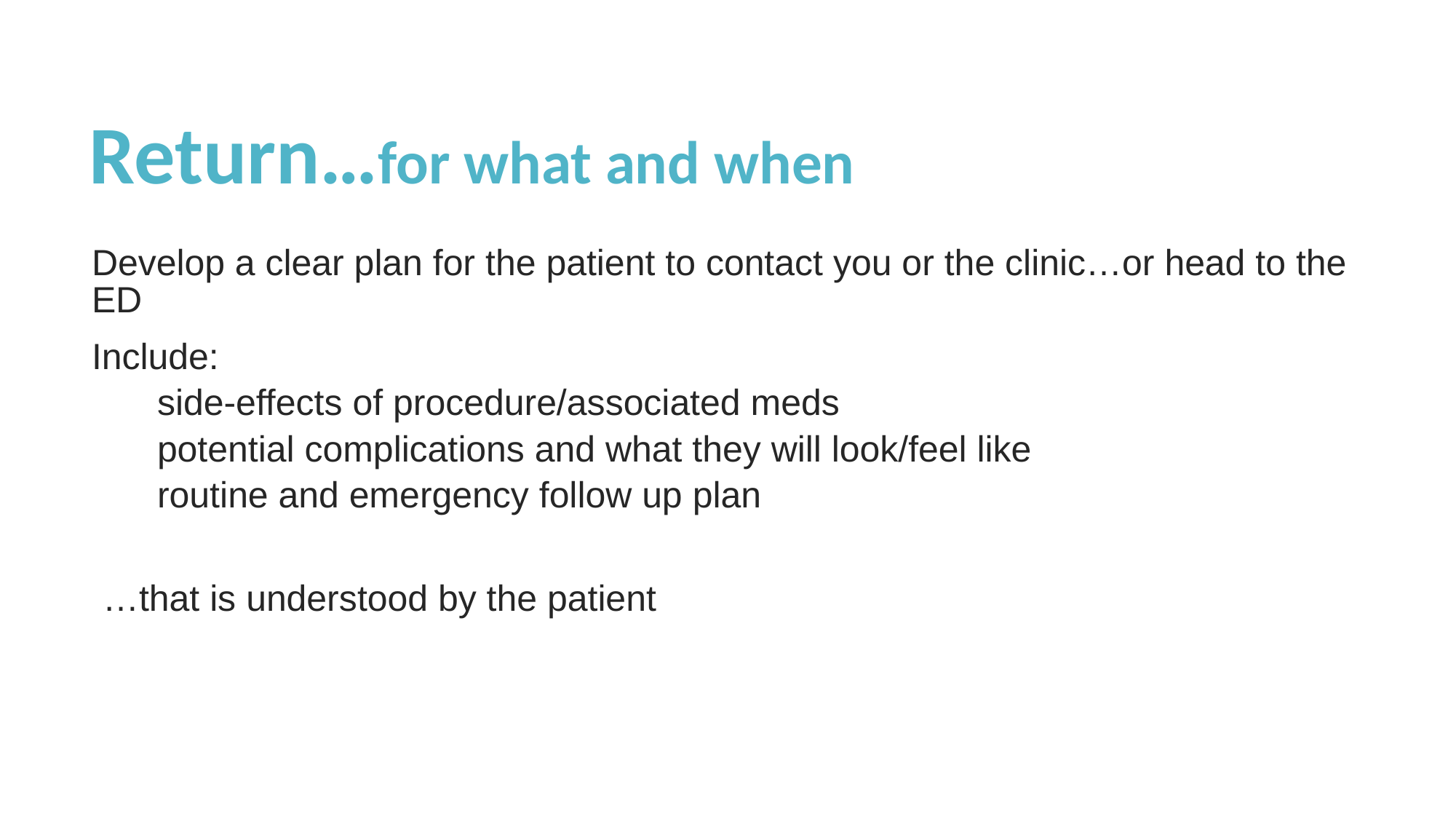

# Return…for what and when
Develop a clear plan for the patient to contact you or the clinic…or head to the ED
Include:
side-effects of procedure/associated meds
potential complications and what they will look/feel like
routine and emergency follow up plan
…that is understood by the patient

## Slide 17
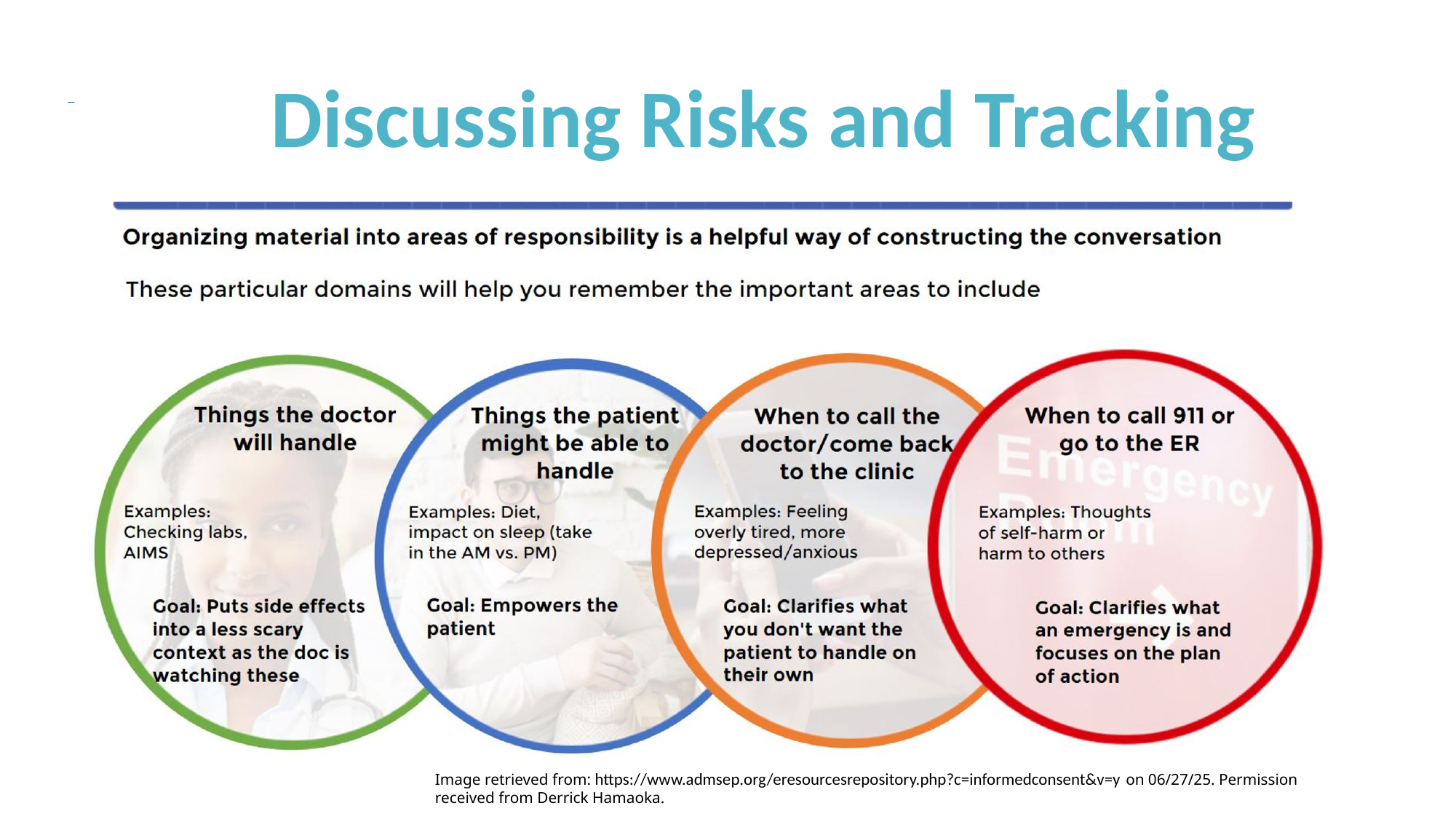

# Discussing Risks and Tracking
Image retrieved from: https://www.admsep.org/eresourcesrepository.php?c=informedconsent&v=y on 06/27/25. Permission received from Derrick Hamaoka.

## Slide 18
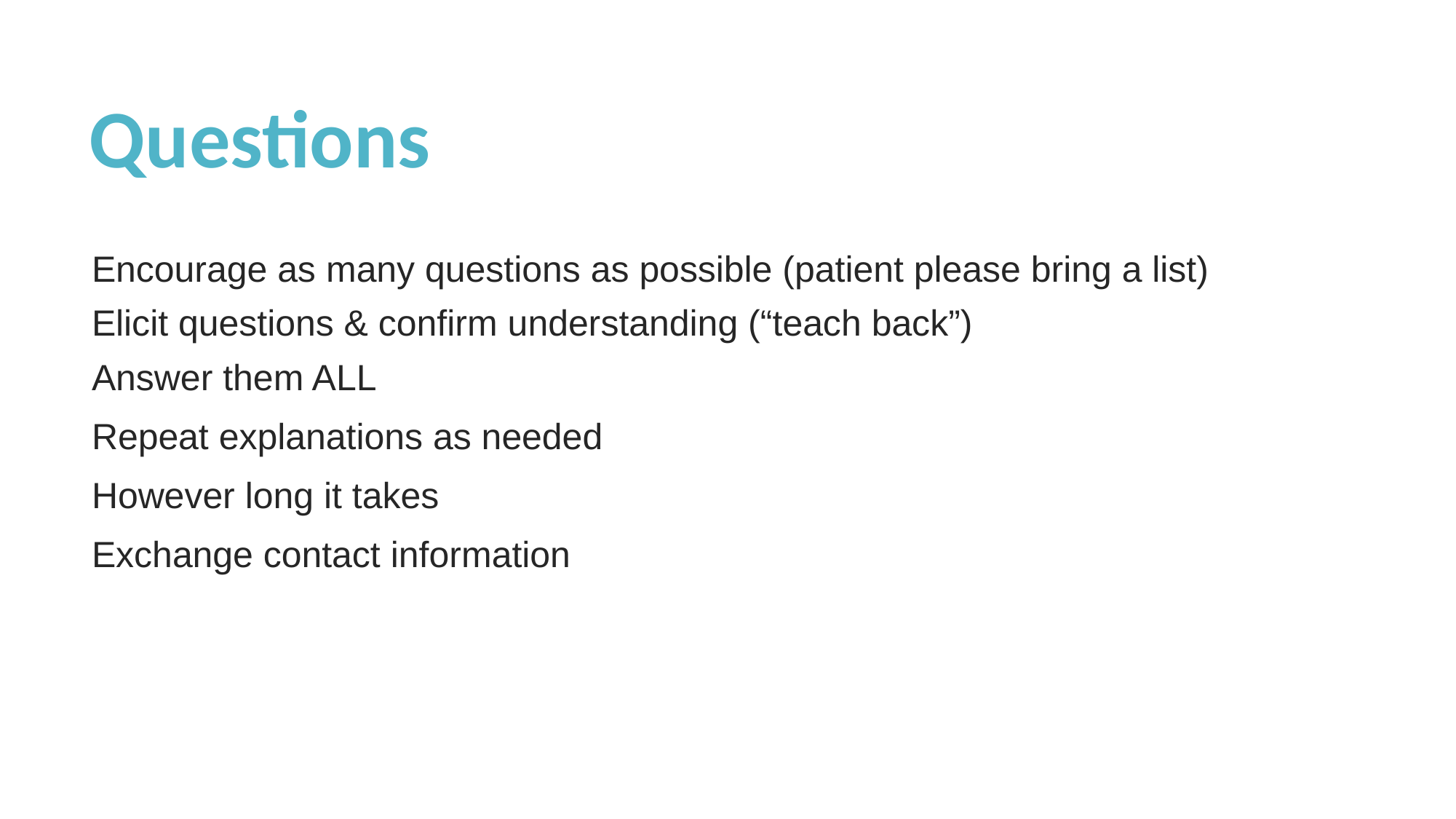

# Questions
Encourage as many questions as possible (patient please bring a list)
Elicit questions & confirm understanding (“teach back”)
Answer them ALL
Repeat explanations as needed
However long it takes
Exchange contact information

## Slide 19
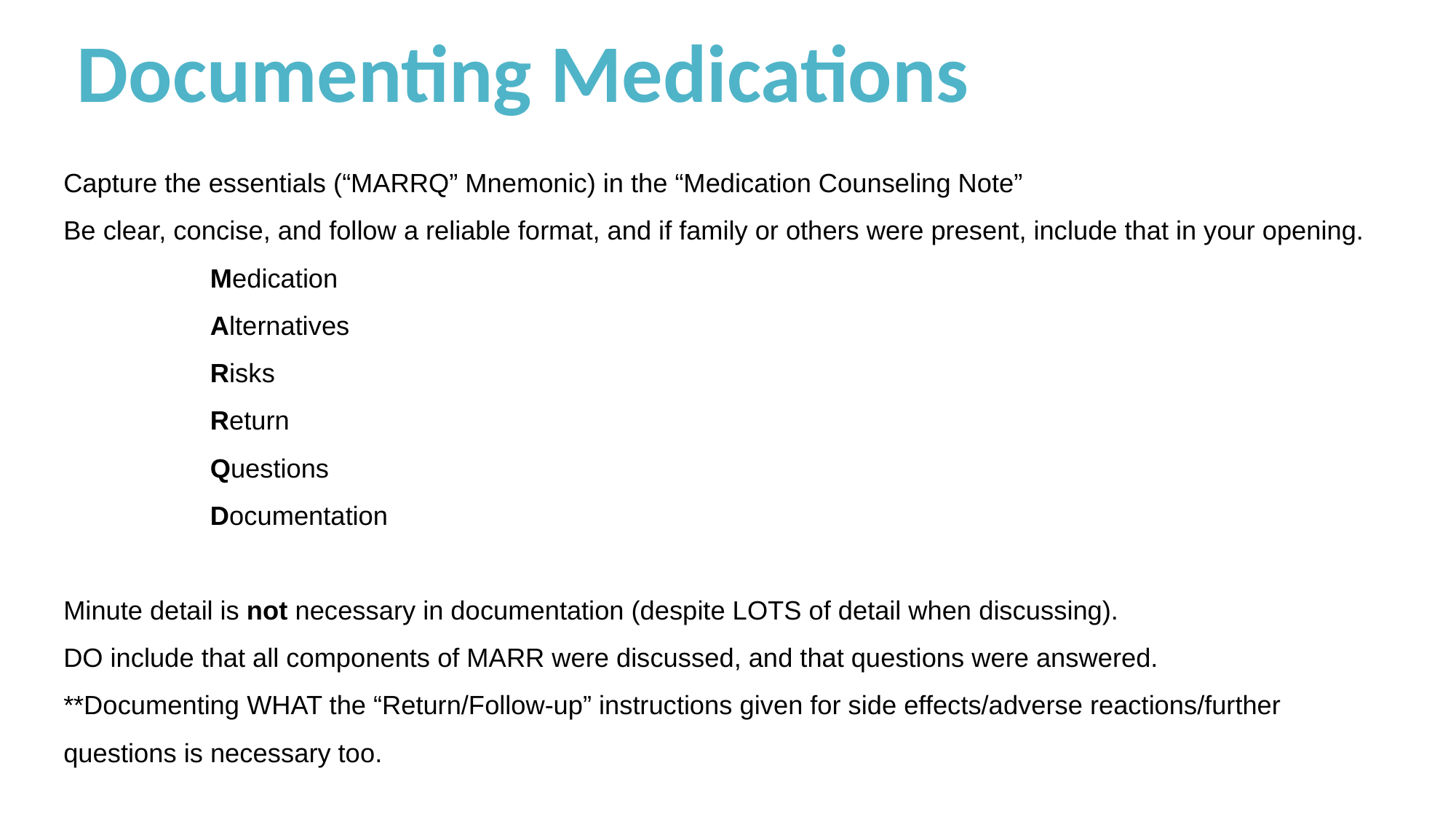

# Documenting Medications
Capture the essentials (“MARRQ” Mnemonic) in the “Medication Counseling Note”
Be clear, concise, and follow a reliable format, and if family or others were present, include that in your opening.
Medication
Alternatives
Risks
Return
Questions
Documentation
Minute detail is not necessary in documentation (despite LOTS of detail when discussing).
DO include that all components of MARR were discussed, and that questions were answered.
**Documenting WHAT the “Return/Follow-up” instructions given for side effects/adverse reactions/further questions is necessary too.

## Slide 20
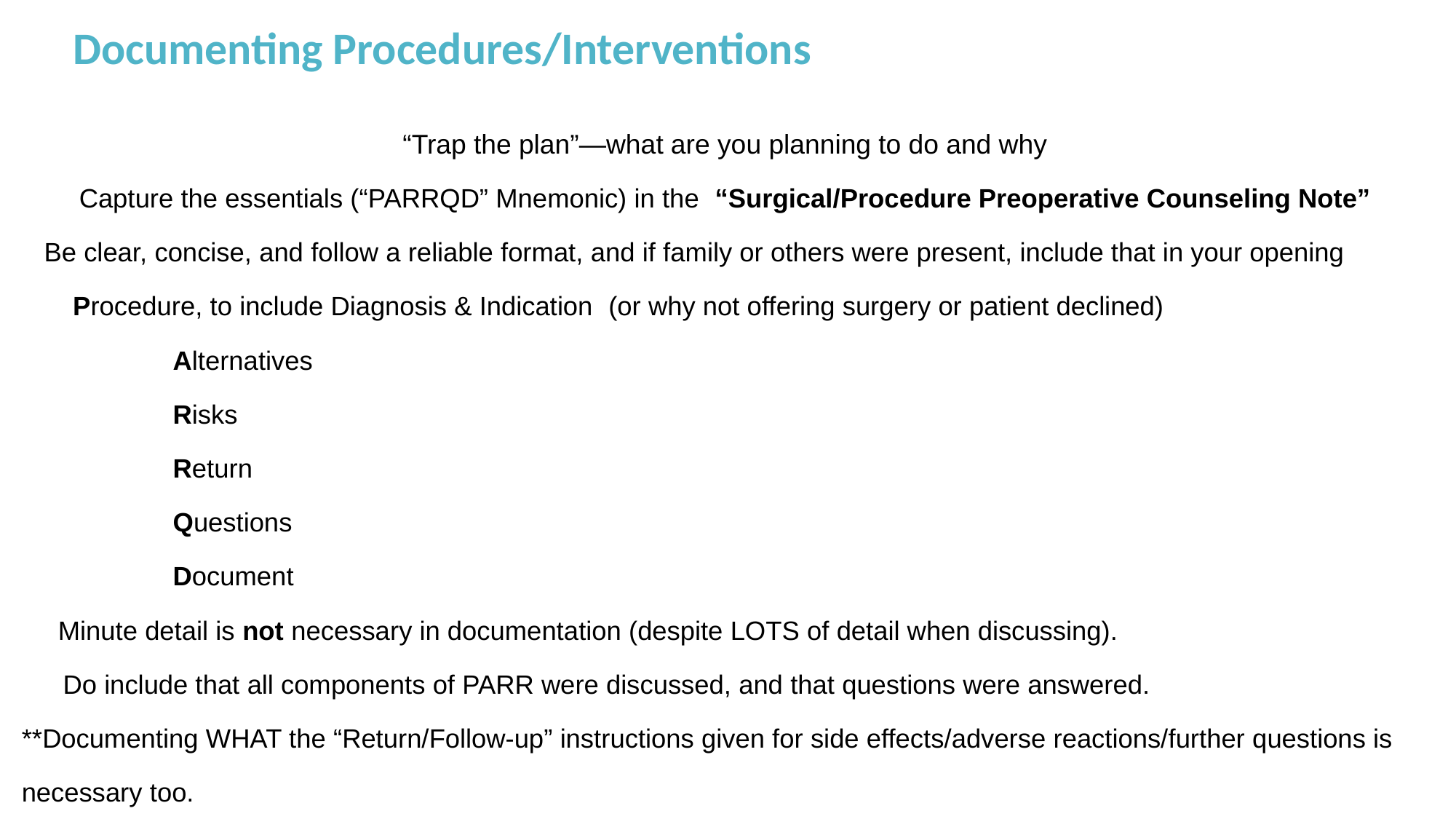

# Documenting Procedures/Interventions
“Trap the plan”—what are you planning to do and why
Capture the essentials (“PARRQD” Mnemonic) in the  “Surgical/Procedure Preoperative Counseling Note”
 Be clear, concise, and follow a reliable format, and if family or others were present, include that in your opening
 			Procedure, to include Diagnosis & Indication  (or why not offering surgery or patient declined)
Alternatives
Risks
Return
Questions
Document
Minute detail is not necessary in documentation (despite LOTS of detail when discussing).
 Do include that all components of PARR were discussed, and that questions were answered.
**Documenting WHAT the “Return/Follow-up” instructions given for side effects/adverse reactions/further questions is necessary too.

## Slide 21
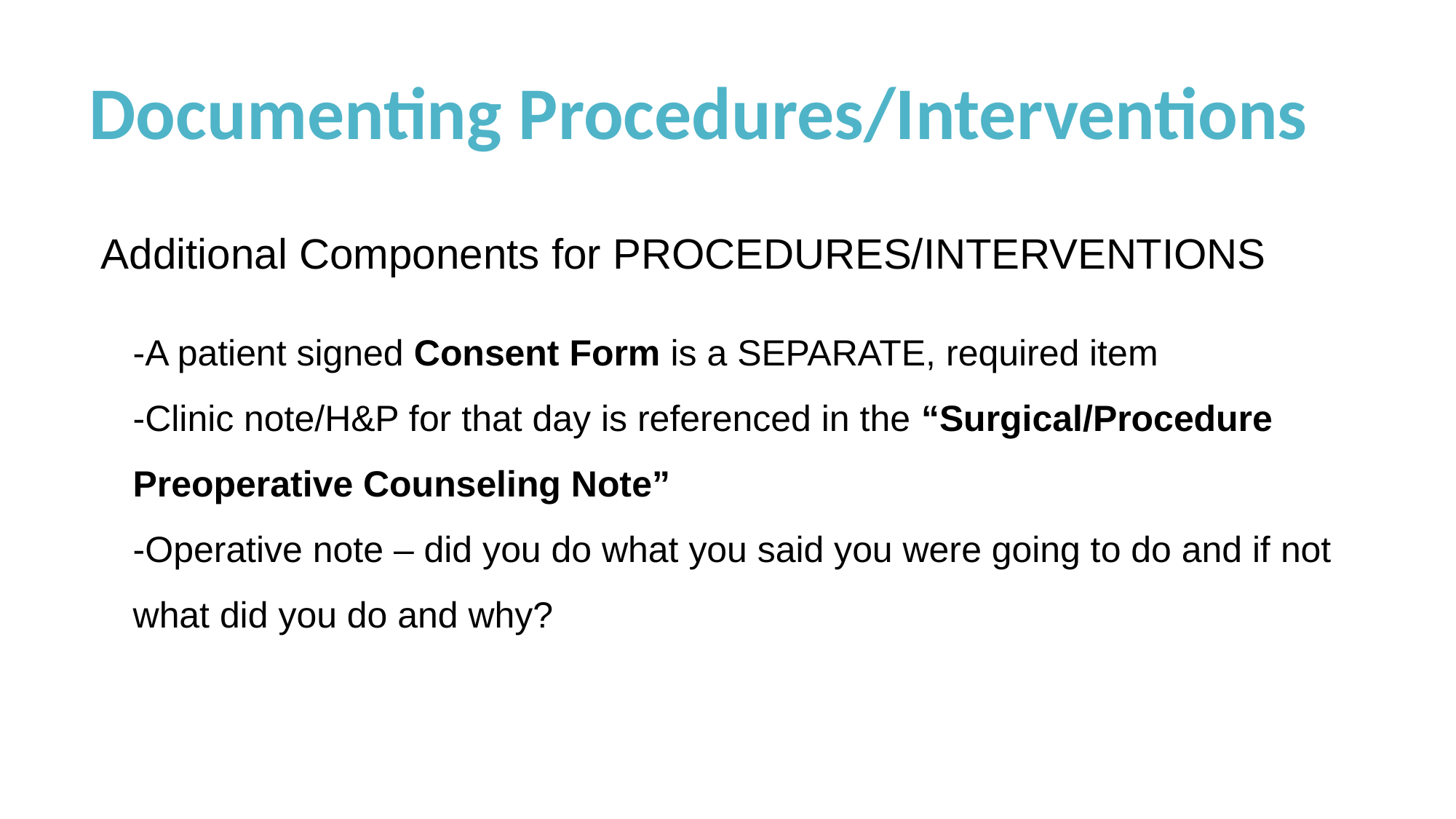

# Documenting Procedures/Interventions
Additional Components for PROCEDURES/INTERVENTIONS
-A patient signed Consent Form is a SEPARATE, required item
-Clinic note/H&P for that day is referenced in the “Surgical/Procedure Preoperative Counseling Note”
-Operative note – did you do what you said you were going to do and if not what did you do and why?

## Slide 22
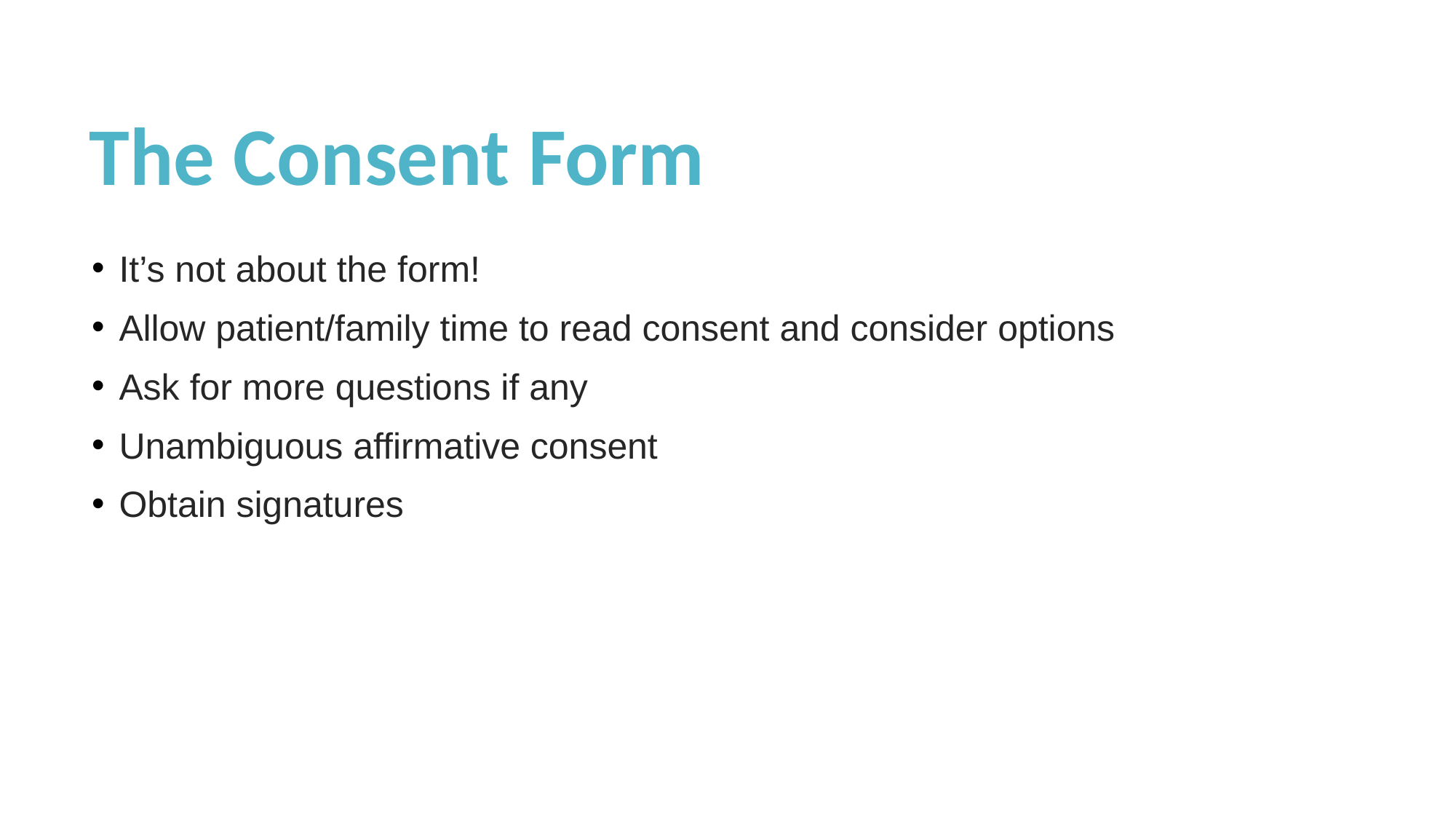

# The Consent Form
It’s not about the form!
Allow patient/family time to read consent and consider options
Ask for more questions if any
Unambiguous affirmative consent
Obtain signatures

## Slide 23
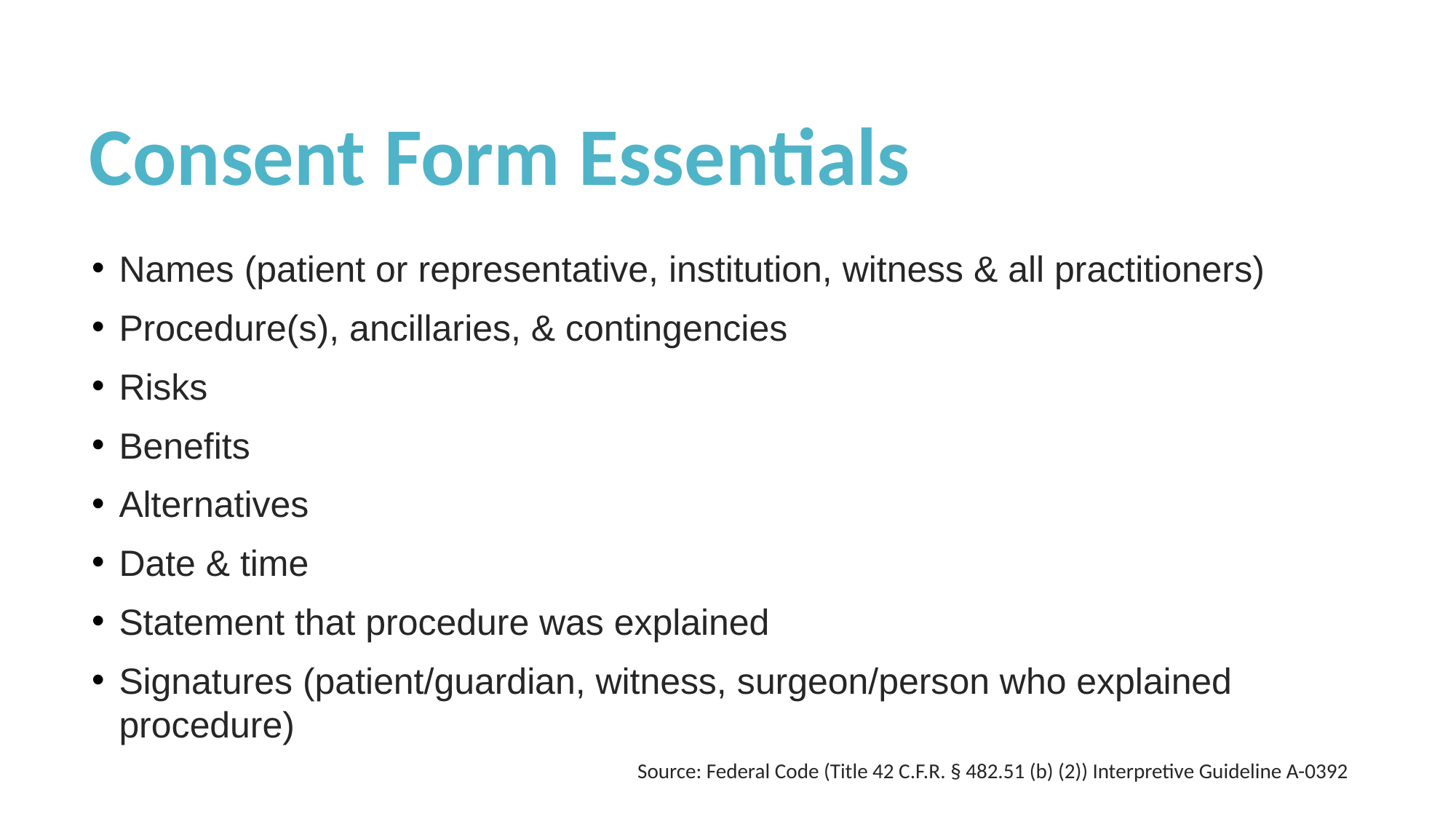

# Consent Form Essentials
Names (patient or representative, institution, witness & all practitioners)
Procedure(s), ancillaries, & contingencies
Risks
Benefits
Alternatives
Date & time
Statement that procedure was explained
Signatures (patient/guardian, witness, surgeon/person who explained procedure)
					Source: Federal Code (Title 42 C.F.R. § 482.51 (b) (2)) Interpretive Guideline A-0392

## Slide 24
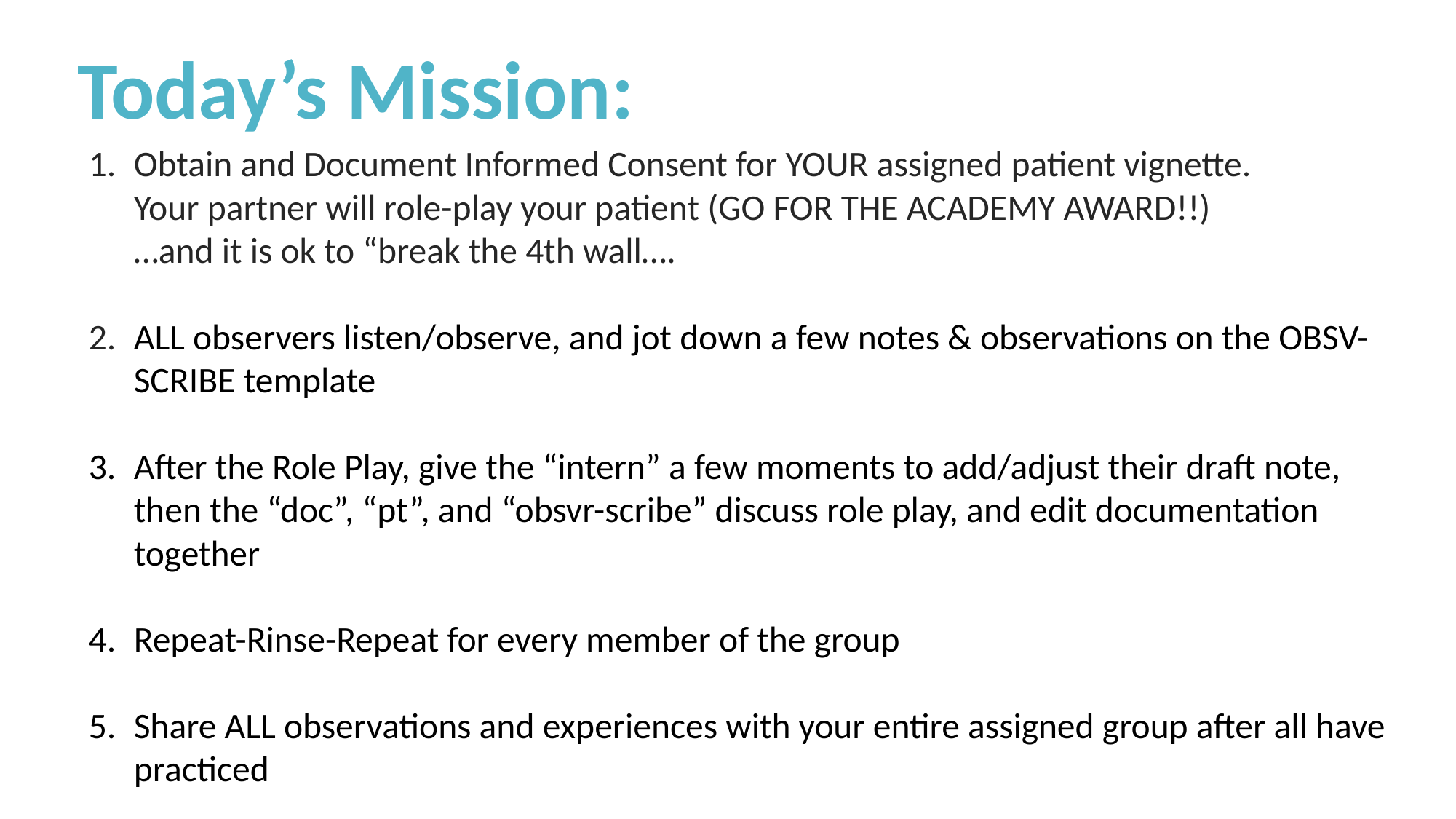

# Today’s Mission:
Obtain and Document Informed Consent for YOUR assigned patient vignette.
Your partner will role-play your patient (GO FOR THE ACADEMY AWARD!!)
…and it is ok to “break the 4th wall….
ALL observers listen/observe, and jot down a few notes & observations on the OBSV-SCRIBE template
After the Role Play, give the “intern” a few moments to add/adjust their draft note, then the “doc”, “pt”, and “obsvr-scribe” discuss role play, and edit documentation together
Repeat-Rinse-Repeat for every member of the group
Share ALL observations and experiences with your entire assigned group after all have practiced

## Slide 25
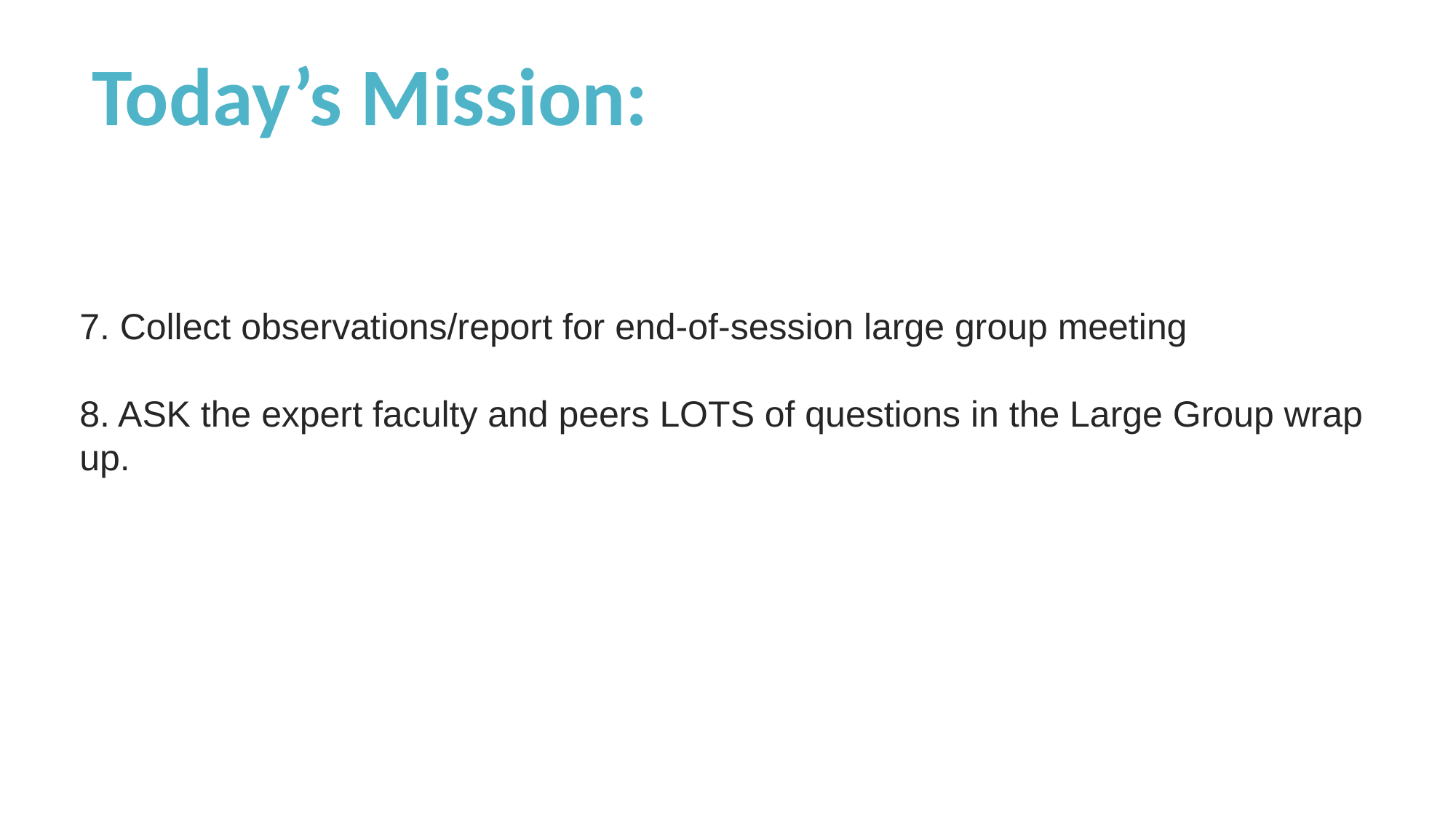

# Today’s Mission:
7. Collect observations/report for end-of-session large group meeting
8. ASK the expert faculty and peers LOTS of questions in the Large Group wrap up.

## Slide 26
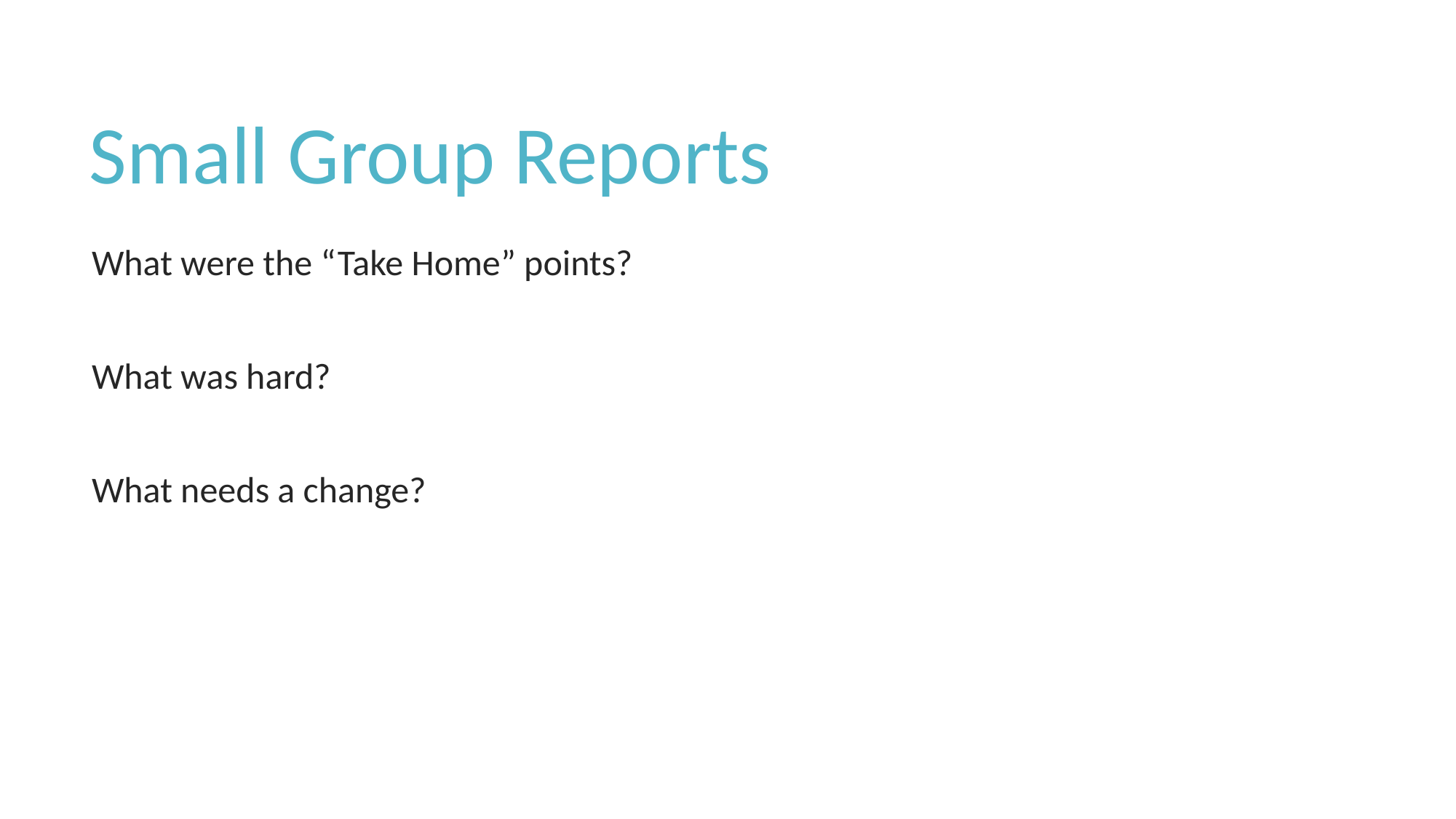

# Small Group Reports
What were the “Take Home” points?
What was hard?
What needs a change?

## Slide 27
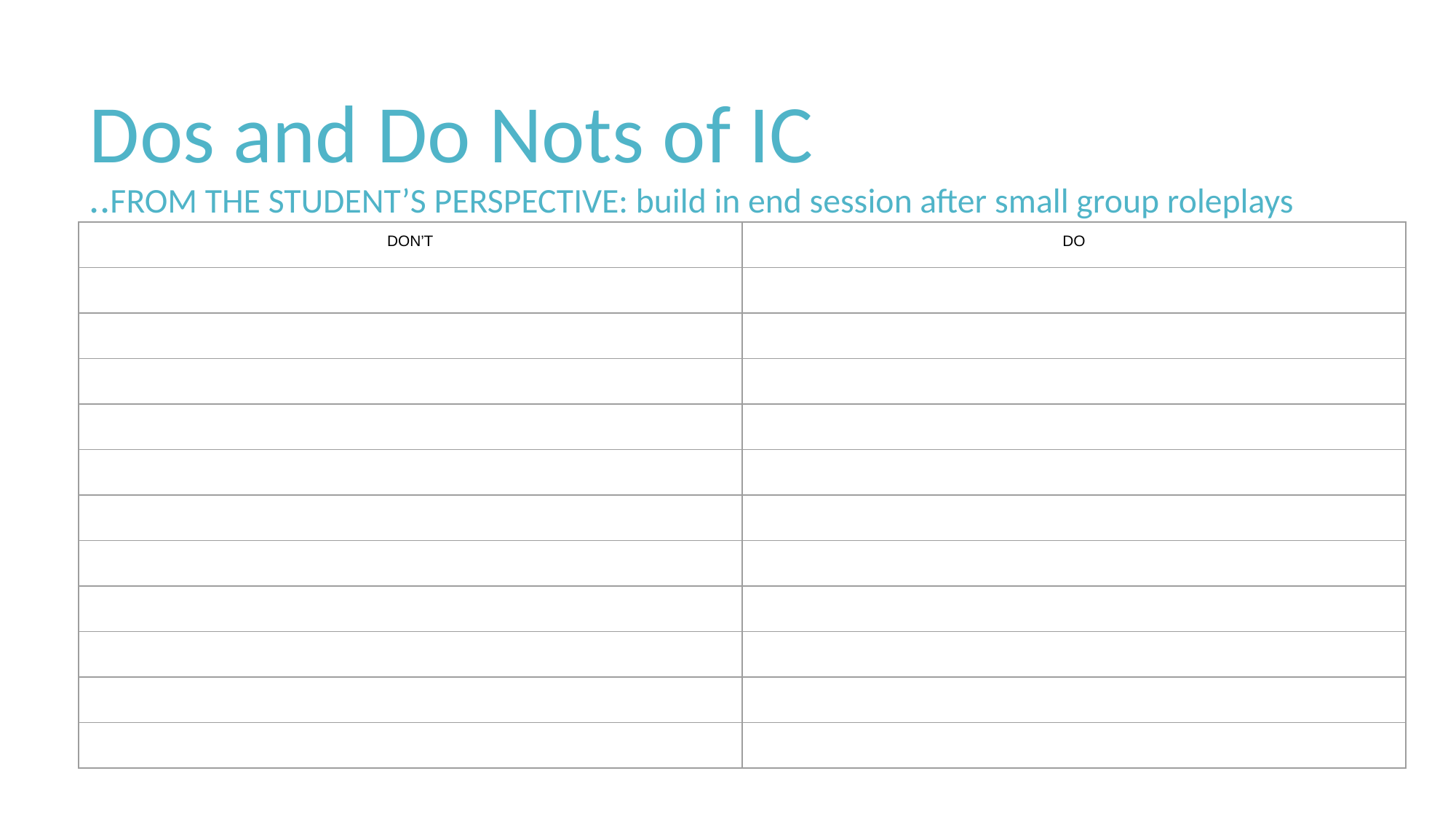

# Dos and Do Nots of IC
..FROM THE STUDENT’S PERSPECTIVE: build in end session after small group roleplays
| DON’T | DO |
| --- | --- |
| | |
| | |
| | |
| | |
| | |
| | |
| | |
| | |
| | |
| | |
| | |

## Slide 28
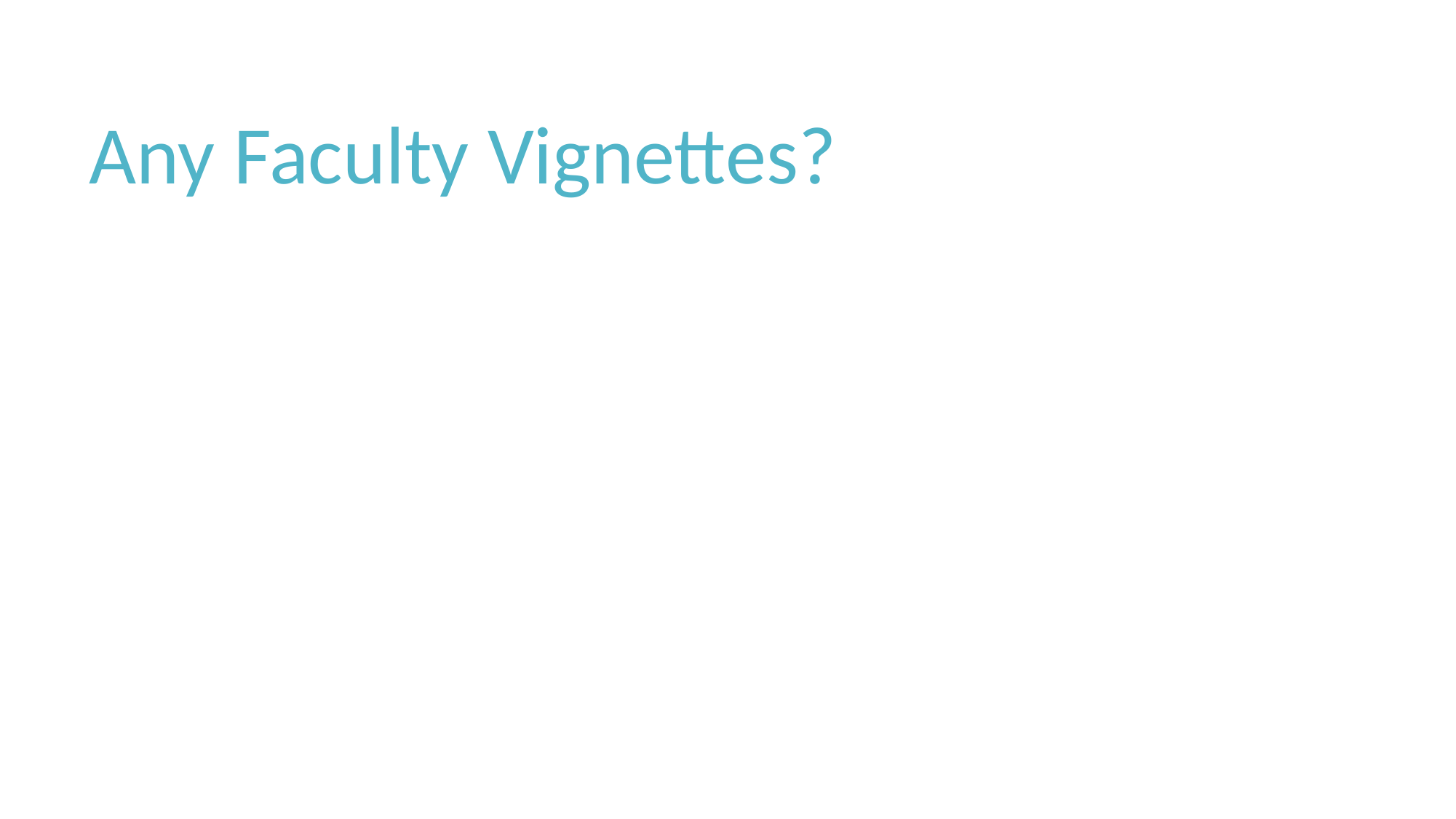

# Any Faculty Vignettes?

## Slide 29
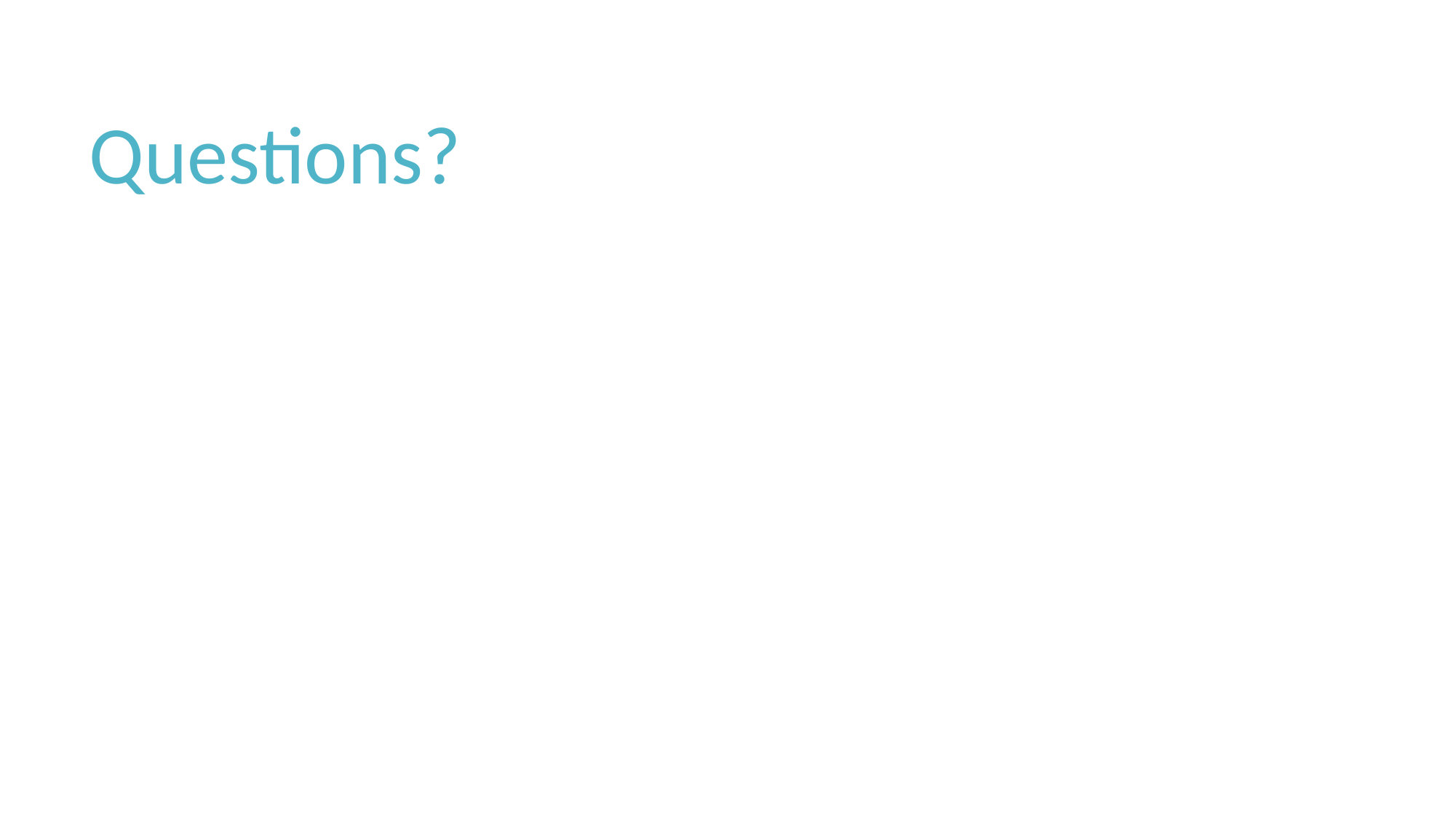

# Questions?
