# Supplementary material for: Obtaining and Documenting Informed Consent: An Advanced UME Cross-Specialty, Role-Playing Skill Builder
Source: MedEdPORTAL. 2026 Mar 3;22:11580. doi: 10.15766/mep_2374-8265.11580 (PMC12956033; doi:10.15766/mep_2374-8265.11580)
Supplement: Supplementary file 1 — Course Syllabus.docxPrereadings.pdfStatPearls Article.pdfADMSEP eModule folderClinical Vignettes.pdfRubric.pdfMARRQD, PARRQD Templates.docxOrientation.pptxObserver-Scribe Template.docxVignette Answers.pdf [file mep_2374-8265.11580-s001.zip › I. Observer-Scribe Template.docx]

OBSERVER-SCRIBE:

Jot observations: “*medical explanation at too high level for pt”, “*

CIRCLE if discussed:

 CROSS OUT if missed:



Students are to be provided with the observer-scribe templates during the small group roleplay sessions (may be provided ahead of the course so that students may print and bring these to the synchronous session).

|  | **Medication (MARRQD)** |
| --- | --- |
| **Procedure** | **Medication/Intervention: [general description appropriate for patients (think 8^th^ grade education)]**  **Indication/Expected Benefits/likelihood of success (Target” sx and intended effect)**  **Contraindications**  **Mech of Action**  **Key Steps: Dosage, Expected Course/Duration** |
| **Alternatives** | **Alternate Treatments**  **Course WITHOUT medication** |
| **Risks** | **Common/expected Side Effects (and work-arounds, like stool softeners, etc)**  **“Major” & “Minor” Side Effects/ Complications**  **Adverse Reactions/Toxicities of accompanying meds** *Include remote but severe possibilities |
| **Return** | **Things for patient to watch for:**  **Things for patient to return for:**  **Standard/expected follow up plan (include WHEN and WHERE to return for Follow Up)** |
| **Document** | **Medication Counseling Note:**  (a short, effective, appropriate EHR/EMR counseling note in this box  include statement that Questions were addressed)  **Sub-group review/edit together** |

OBSERVER-SCRIBE:

Jot observations: “*medical explanation at too high level for pt”, “*

CIRCLE if discussed: CROSS OUT if missed:

|  | **Procedure (PARRQD)** |
| --- | --- |
| **Procedure** | **Procedure/Intervention: [general description appropriate for patients (think 8^th^ grade education)]**  **Indication/Expected Benefits/likelihood of success (Target” sx and intended effect)**  **Contraindications**  **Key steps/Expected course (**Anesthesia, drugs, blood, tubes & lines, recovery, rehab, nursing care, etc) |
| **Alternatives** | **Alternate Treatments**  **Course WITHOUT procedure** |
| **Risks** | **Common/expected Side Effects (and work-arounds, like stool softeners, etc)**  **Side Effects**  **“Major” & “Minor” complications**  **Adverse Reactions/Toxicities of accompanying meds** |
| **Return** | **Things for patient to watch for:**  **Things for patient to return for:**  **Standard/expected follow up plan (include WHEN and WHERE to return for Follow Up)** |
| **Document** | **Surgical/Procedure Preoperative Counseling Note**  **Sub-group review/edit together**  (a short, effective, appropriate EHR/EMR counseling note in this box  include statement that Questions were addressed) |
