# Supplementary material for: Obtaining and Documenting Informed Consent: An Advanced UME Cross-Specialty, Role-Playing Skill Builder
Source: MedEdPORTAL. 2026 Mar 3;22:11580. doi: 10.15766/mep_2374-8265.11580 (PMC12956033; doi:10.15766/mep_2374-8265.11580)
Supplement: Supplementary file 1 — Course Syllabus.docxPrereadings.pdfStatPearls Article.pdfADMSEP eModule folderClinical Vignettes.pdfRubric.pdfMARRQD, PARRQD Templates.docxOrientation.pptxObserver-Scribe Template.docxVignette Answers.pdf [file mep_2374-8265.11580-s001.zip › J. Vignette Answers.pdf]

Students are to be provided with the informed consent template answer keys after the synchronous session.

Medication: ESZOPICLONE

a. Eszopiclone (Lunesta) Med-Psych Liz Greene

Patient is a 52-year-old woman with h/o hypothyroidism, recently diagnosed with obstructive sleep apnea, and placed on CPAP by the sleep medicine clinic. She has an appointment in the Sleep Clinic with a chief complaint of difficulty sleeping, specifically falling asleep and staying asleep with the CPAP on, feeling unrested and irritable in the morning upon awakening. She also has tried following the CBT for insomnia app suggestions, but is unable to work through the wearing of her CPAP. She has no history of depression, anxiety, or substance misuse/abuse. She has no other medical problems, and has medical decision-making capacity. You and your attending have determined that a trial of eszopiclone might be helpful in treating her sleep difficulty and assist in her use of CPAP.

a. Complete your "Informed Consent" study/prep card
b. Be prepared to role-play the role of the intern/physician in obtaining informed consent
c. Write an Informed Consent Note for the electronic medical record (EMR) AFTER the role play (hint: develop an outline for this case for your use)

Medications (MARRQD)	
**Medication: (general description appropriate for patients (think 8th grade education)**<br><br>Eszopiclone is a medication that will help you fall asleep more easily and stay asleep a little longer. It works by helping your brain respond more easily and strongly to your own internal chemicals that calm down your brain cells so you can sleep.<br><br>**Indication/Expected Benefits/likelihood of success**<br>Eszopiclone works well to improve sleep in most people, particularly when taken for short periods of time (a few weeks). It usually will work within 30 minutes.<br><br>**Contraindications**<br>You should not take Eszopiclone if you are taking other medications that make you sleepy, as this combination can reduce your drive to breathe.<br><br>**Mech of Action**<br><br>Eszopiclone works by helping your brain respond more easily and strongly to your own internal chemicals that calm down your brain cells so you can sleep.<br><br>**Key Steps: Dosage, Expected Course/Duration**<br><br>You will take 2mg of eszopiclone at night immediately before bedtime for the next two weeks. It is better to take eszopiclone a few hours after eating. After you take eszopiclone you should go to bed immediately and stay in bed for a full eight hours.<br><br>The goal of treatment is to help give you some time to adjust to your CPAP treatment. After two weeks we will stop eszopiclone and see how you are doing.	**Medication**

Medication: ESZOPICLONE

**Alternate Treatments**<br>Other medications that could be used for sleep include relatives of eszopiclone like zolpidem, and sedating medications in a different family like trazodone.<br><br>The best treatments for trouble sleeping are not medication based; they are based on behavior changes and changes to your thinking. Medication works best if used only for short periods of time.<br><br>**Course WITHOUT treatment**<br>Without treatment it is likely that eventually your difficulty sleeping with your CPAP machine will slowly improve. However, there is a risk that you might give up on using your CPAP machine which would leave your obstructive sleep apnea untreated which is a risk to your health. Additionally during the time you are not sleeping well you are likely to feel sleepy during the day which can place you at higher risk for accidents and injuries, as well as having a negative impact on your mood and your ability to concentrate.	**Alternatives**
**Common/expected Side Effects (and work-arounds, like stool softeners, etc)**<br><br>Eszopiclone causes an unpleasant taste in the mouth for about 1/3 of people. Some people have found that they can reduce the taste by taking eszopiclone with an acidic drink like orange juice but this does not seem to work for everyone.<br><br>Eszopiclone is intended to make you sleepy, but some patients find that it lasts too long into the next day and makes them feel groggy. The best way to manage this is to take eszopiclone and go to bed earlier in the evening and ensure you remain in bed for at least 8 full hours. If eszopiclone results in too much daytime sedation you will need to switch to a different treatment.<br><br>**"Major" and "Minor" Side Effects/complications**<br>Some patients will experience events while taking eszopiclone where they will carry out actions such as eating or driving or talking to others but not remember those actions later. We believe this occurs because eszopiclone is helping to calm the activity in your brain cells, including those that help you form memories. The best way to avoid this side effect is to take eszopiclone immediately before bedtime and remain in bed for a full eight hours until the medication has fully worn off. If this side effect occurs you will need to contact us immediately and we will help you switch to a different treatment.<br><br>Some patients will become tolerant to eszopiclone, which means it will stop working for them after a period of time. If this occurs you will need to contact us and we will help you stop taking eszopiclone and either change to a different medication or consider non-medication strategies to help you sleep.<br><br>Rarely, patients will become dependent on eszopiclone which means they can not sleep without it. Even more rarely patients become addicted to eszopiclone which means they want to use the	**Risks**

medication more often and in higher doses than it was prescribed. If this occurs we would work with you to slowly and safely decrease eszopiclone and find other solutions to help you sleep.<br><br>**Adverse Reactions/Toxicities**<br>Very rarely some patients develop a condition called angioedema, a swelling in the throat, after taking eszopiclone. If this occurs you should seek immediate emergency care by calling 911 and never take eszopiclone again.<br><br>*Include remote but severe possibilities	
**Things for patient to watch for:**<br>Feeling too sleepy in the morning<br>Lost memory events<br>Medication stops working<br>Swelling in the throat (emergency!)<br><br>**Things for patient to return for:**<br>Too sleepy in the morning and taking medication earlier does not help, lost memory events, medication stops working.<br><br>If swelling in the throat seek emergency care immediately by calling 911.<br><br>**Standard/expected follow-up plan:**<br>Return in two weeks to discuss progress and stop medication.	**Return**
**Medication Counseling Note** (a short, effective, appropriate EHR/EMR counseling note in this box)<br><br>I discussed the expected benefits, possible alternatives, and risks of eszopiclone with the patient. Risks discussed included but were not limited to risks of unpleasant taste, oversedation, lost memory events, tolerance, dependence, and angioedema. The patient considered all information and asked appropriate questions which were answered. The patient consented to a trial of eszopiclone for short term use to address insomnia related to initial adjustment to CPAP treatment.	**Document**

** Remember to allow questions from the patient

b. Oral Contraceptives OB-GYN Logan Peterson

Patient is a 32 y/o female who presents to your office desiring contraception. You discuss her reproductive health plans and review her prior contraceptive use. Previously, she used Depo medroxyprogesterone but did not like some of the side effects and she now desires an alternative method. She consistently uses condoms to reduce the risk of sexually transmitted infections. She has no significant medical history and is in good health. Through shared decision-making and after review of her contraceptive options she desires to begin using oral combined hormonal contraceptive pills.

a. Complete your "Informed Consent" study/prep card
b. Be prepared to role-play the role of the intern/physician in obtaining informed consent
c. Write an Informed Consent Note for the electronic medical record (EMR) AFTER the role play (hint: develop an outline for this case for your use)

Medications (MARRQD)	
**Medication: (general description appropriate for patients (think 8th grade education)**<br>Daily pill that can be taken to significantly reduce the risk of pregnancy. There are two types of pills: monophasic and tri-phasic. Monophasic have the same amount of hormone every day during the active pills while tri-phasic have varying amounts of hormone each day to more closely mimic a natural cycle. In actual practice, the tri-phasic pills do not offer any appreciable benefit over monophasic and result in more unscheduled bleeding and may not control heavy menstrual bleeding as well as monophasic. Generally, monophasic are recommended as first line.<br><br>**Indication/Expected Benefits/likelihood of success**<br>Indication: Contraception and/or cycle/bleeding control<br>Benefits: highly reliable contraception and cycle control if taken correctly<br>Efficacy: Perfect-use failure 0.3%; typical-use failure 7% (this is the number we use to counsel patients as people are not perfect and will forget a pill or take incorrectly or not resume active pills when indicated)<br>Decreased risk of development of ovarian and endometrial cancers<br><br>**Contraindications**<br>See CDC MEC Chart: HTN, VTE, h/o stroke, heart disease, current breast cancer, severe liver disease, migraines with aura, Age >35 AND smoker, malabsorptive bariatric surgery to name a few.<br><br>**Mech of Action**<br>The main contraceptive efficacy of COCs is suppression of ovulation by inhibition of gonadotropin-releasing hormone (GnRH) from the hypothalamus, as well as inhibition of both luteinizing hormone (LH) and follicle-stimulating hormone (FSH), and disruption of the mid-cycle LH surge. These effects are mediated by both the progestin and estrogen component of the COC working synergistically. Progestin renders the endometrium less suitable for implantation, thickens cervical mucus, which becomes less permeable to penetration by sperm, and impairs normal tubal motility and peristalsis.	**Medication**

**Key Steps: Dosage, Expected Course/Duration**<br>Can be taken in 2 ways:<br>Cyclic- take all the active pills in a pack and then the placebo pills to have a withdrawal bleed (period).<br>Continuous- take all active pills in the pack and then skip the placebo pills and immediately proceed to next pack of pills to skip withdrawal bleed. Some packs are made specifically for this use and will not contain placebo pills but will instead have 3 months of active pills in a single pack.<br>Duration of use can continue as long as the patient desires and no contraindications to use are developed. May continue use until menopause.	
**Alternate Treatments**<br>Abstinence, natural family planning, condoms, vaginal ring, patch, progesterone injection, implant, IUD, permanent sterilization (tubal ligation or vasectomy), to use nothing<br><br>**Course WITHOUT treatment**<br>~30% chance of pregnancy per cycle with adequate sperm exposure if not using contraception.	**Alternatives**
**Common/expected Side Effects (and work-arounds, like stool softeners, etc)**<br>Nausea, breast tenderness, headaches- generally mild and resolve within 1-2 months of initiation<br>Unscheduled bleeding- generally resolves within 1-2 months of initiation; does not change contraceptive efficacy<br>Amenorrhea- may occur after prolonged cyclic use or with continuous use. Not worrisome and if patients are concerned for pregnancy may take home pregnancy test for reassurance<br><br>**“Major” and “Minor” Side Effects/complications**<br>Libido changes- may have decrease in sex drive related to decrease in free circulating androgens<br>HTN, VTE, MI, stroke, lipid changes<br><br>**Adverse Reactions/Toxicities**<br>Antiepileptic meds- see CDC MEC for specific list. Should generally not use COCs because certain antiseizure medications may reduce the efficacy of the hormonal contraceptive<br>Rifampin, griseofulvin, St. John’s wort- may have reduced contraceptive efficacy<br><br>**Include remote but severe possibilities*	**Risks**
**Things for patient to watch for:**<br>Signs/symptoms of VTE, MI, intolerable side effects, inability to take pill or remember to take pill daily.<br><br>**Things for patient to return for:**<br>Mild side effects not resolving after several cycles on pills, pregnancy or concern for pregnancy, concern for STI.	**Return**

**Standard/expected follow-up plan:** as needed	
**Medication Counseling Note** **(a short, effective, appropriate EHR/EMR counseling note in this box)**<br>The patient was counseled extensively on birth control options including male and female condoms, oral contraceptives including progesterone only and combined OCs, vaginal ring, diaphragm, patch, injectable progestin, subdermal implant, progestin IUDs and copper IUD, as well as male and female sterilization. Discussed how each is used, failure rates, reversibility, side effects. Reviewed that hormonal methods of birth control may have side effects related to them: nausea, HA, irregular menses, and spotting. Also reviewed that they are easily reversible via discontinuation. Discussed that failure rates are best with those forms of contraception that do not require the patient to remember to use, and that the three reversible long term contraceptives (implant and IUDs) have failure rates that are 1/10 or less of oral contraceptives, with an overall failure rate of less than 1% for these long-term contraceptives. Failure rates (approximately 85% with nothing, 20-40% with diaphragm, 10-15% with condoms, 5-10% with OC, 5% with vaginal ring and patch, 1-4% with injectable progestin, less than 1% with implant and IUDs) were discussed. Reviewed that only condoms reduce the risk of STI transmission.<br><br>After discussion of her contraceptive options and review of her medical history the patient elected to proceed with OCP initiation. Instructions for daily use were provided. The risks and benefits were again reviewed and all questions answered.	**Document**

** Remember to allow questions from the patient

c. Alteplase Neuro-ED Maggie Swanberg

A 34 y/o active duty SSG is in the ED after developing left arm and leg weakness while eating breakfast this morning. He woke at 05:30, performed his usual PT and went to the DFAC around 07:45. He developed worsened weakness, and he arrived in the ED around 08:30. His neuro exam was notable for 4/5 strength of the left arm and leg, (+) pronator drift on the left and reflexes were 3 on the left and 2 on the right. Neurology saw the patient at 08:50 and feel this patient is a good thrombolysis candidate. You are asked to obtain informed consent on this patient for the administration of alteplase. He has no significant medical history and is in good health. He is able to engage in shared decision making.

a. Complete your "Informed Consent" study/prep card
b. Be prepared to role-play the role of the intern/physician in obtaining informed consent
c. Write an Informed Consent Note for the electronic medical record (EMR) AFTER the role play (hint: develop an outline for this case for your use)

	**Procedure (PARRQD)**
**Procedure**	**Procedure/Intervention: (general description appropriate for patients (think 8th grade education)**<br>Alteplase is recommended for the swift treatment of blood clots in the heart, brain, and the lungs. Your left arm and leg weakness seems to be caused by a blood clot in your brain, and with this injection, we hope to break up the clot which is causing a "traffic jam" in your blood vessel, to prevent severe damage to your brain and continued difficulty using your left arm and leg. Alteplase needs to be injected within several hours of the problem, and you are in that window.<br>Getting this treatment after a stroke reduces your risk of disability. People who get alteplase to treat their stroke have a better chance of recovering without disability and getting back to the activities they love compared to people who do not receive the treatment. All medicines have some risk. With alteplase, there is a risk of serious bleeding. However, time is important as well. You have few risk factors, and are young, so your risk for a complication from the treatment is much lower than the risk of permanent disability without treatment. We have found the faster alteplase is administered, the greater the chance that patients will have the best possible outcome<br>** see link here for editorial about IC for alteplase:<br>https://www.ahajournals.org/doi/epub/10.1161/STROKEAHA.119.024653<br>and American Academy of Emergency Medicine decision aid, Ischemic Stroke Predictive Risk Score<br>http://www.sorcan.ca/iscore/<br><br>**Indication/Expected Benefits/likelihood of success**<br>Alteplase actually binds to the tough "fibrin" in a blood clot, and converts it into a substance that can be broken down more easily by your body. Patients who receive alteplase have improved function and less residual impairment at the 3 month mark after the treatment compared to patients who did not receive this medication.<br><br>**Contraindications**<br>Have you been taking blood thinners like warfarin/ Coumadin?<br>Have you had any spine or brain surgeries in the last 3 months?<br>Have you had a head injury in the last 3 months?<br>Have you ever been diagnosed with a brain mass/cancer/tumor/lesion?<br>Do you regularly take aspirin or other over-the-counter pain medications like ibuprofen or others? (not an absolute contraindication…requires monitoring<br><br>**Key steps/Expected course**

	medication reduces stroke related disability at 3 months compared to those who did not receive. This is the expected benefit that the patient needs to be aware of, not that their symptoms will immediately be gone.<br><br>We will add the alteplase to sterile water and inject it through a "heplock" or access-point to your vein in your arm. You will need to be monitored for several hours after the infusion to be sure that you have no allergic reactions or unexpected bleeding
**Alternatives**	**Alternate Treatments**<br><br>none<br><br>**Course WITHOUT procedure**<br>Your arm may remain difficult to use, and there may be further weakness as the clot in your brain solidifies and the areas around it are deprived of oxygen and blood for a longer period of time. The risk of permanent disability is higher without treatment than the potential for an adverse reaction with treatment
**Risks**	**Common/expected Side Effects (and work-arounds, like stool softeners, etc)**<br><br>Major risk is Intracerebral hemorrhage which can occur in close to 3-6% of patients who use this medication. Patients are monitored in the ICU with neurosurgery on call for at least the first 24 hours.<br><br>There are usually no side effects other than some tenderness where the IV catheter is placed.<br><br>Be on watch for unusual bruising, pink or brown urine, red or black or tarry stools, coughing up blood, vomiting blood or blood that looks like coffee grounds, headache, or stroke symptoms/new or worsening weakness in your limbs.<br><br>**"Major" & "Minor" complications**<br>see above and below<br><br>**Adverse Reactions/Toxicities of accompanying meds**<br>a reaction that is like an allergic reaction can occur, causing swelling of your lips and tongue. This is more common when a patient is on some blood-pressure medications, which you are not on.<br>Even though the risk is small, this is why we monitor you very closely for the first few hours to 3 days after administration. If you develop lip or tongue swelling, we will immediately treat you with antihistamines and other allergy treatments, to include using a tube to assist you if your tongue is in the way of breathing.<br><br>Although we think you have a blood clot and not a bleeding problem in your brain, rarely, alteplase can cause acute bleeding in the brain. This is most common in the first 3 days after treatment, so we will also monitor you for any adverse reactions to this infusion.<br><br>It is rare, but some patients who receive alteplase may develop a "cholesterol" clot that may then require surgery to remove it, but sometimes, this can happen in a place that causes irreversible consequences such as blocking the blood flow to your kidneys, eyes, fingers, pancreas or your heart.<br>*Include remote but severe possibilities

**Return**	**Things for patient to watch for:**<br><br>See below<br><br>**Things for patient to return for:**<br><br>unusual bruising, pink or brown urine, red or black or tarry stools, coughing up blood, vomiting blood or blood that looks like coffee grounds, headache, or stroke symptoms/new or worsening weakness in your limbs.<br><br>**Standard/expected follow up plan:**<br><br>PCM and neuro f/u inpatient and outpt
**Document**	**Surgical/Procedure Preoperative Counseling Note**<br>(a short, effective, appropriate EHR/EMR counseling note in this box)<br><br>The patient is a 35 yr old male with no previous medical history, or medication/drug use, who presented with left arm and leg weakness consistent with ischemic stroke., was evaluated by neurology, and is determined to be a candidate for thrombolytic therapy. We reviewed the advantages and disadvantages of ateplase/thrombolytic therapy and the alternatives, to include no treatment. We discussed risks, to include but not limited to the treatment having no effect, or resulting in orolingual edema, or unexpected bleeding in major organs.<br>The patient verbalized understanding of the above and wishes to proceed with thrombolytic therapy in order to potentially reduce further morbidity. All questions answered and all concerns addressed.

** Remember to allow questions from the patient

Procedure:_PICC

1. Insertion of a PICC-Line for IV Antibiotics: Peds-ID Marty Ottolini

This patient is a 14-year-old male with a hx of trauma to the left knee during soccer practice. His knee is warm, swollen, and moderately tender with weight bearing on walking. It was drained by orthopedics 2 days ago who thought it was primarily a traumatic hematoma, and the patient had a reduction but not complete relief of symptoms. Though the fluid evaluation looked like blood on gram stain and cell counts, it grew a methicillin-sensitive *Staphylococcus aureus* within 24 hours. Orthopedics does not think it is serious enough for another procedure but on consulting with pediatric infectious diseases agrees to letting them begin IV therapy with a first-generation cephalosporin. The peripherally inserted central catheter (PICC)-line nursing team is consulted and they plan to insert a peripherally inserted catheter for home IV antibiotics for at least the next several days. There is no significant medical history, and he is on no medications. He is alert and participating in care, with both biological parents present who also are engaged appropriately in his care. Consent must be obtained from the parents, but the child should also be engaged in the discussion and "assent" to the procedure.

a. Complete your "Informed Consent" study/prep card

b. Be prepared to role-play the role of the intern/physician in obtaining informed consent (NOTE: parents needed for Role Play…how fun!)

c. Write an Informed Consent Note for the electronic medical record (EMR) AFTER the role play

	Procedure (PARRQD)
Procedure	**Procedure/Intervention: (general description appropriate for patients (think 8th grade education)**<br>You (your son) has a serious staph infection in his left knee. Our best ability to treat it is to hit it hard in the most likely way to get rid of it with high dose intravenous antibiotics right now. We need to be able to give you (your son) antibiotics through an IV, which allows us to deliver a higher dose that gets the right amount of medicine into the knee joint high enough to thoroughly kill the bacteria.<br><br>**Indication/Expected Benefits/likelihood of success**<br>If we can use IV antibiotics for at least several days, we have a better than 90% chance of getting rid of it in one course of therapy, and reducing the risk of damage to your (his) joint.<br><br>**Contraindications**<br>We have a few different antibiotics we can choose. We are going to go over your (his) history and work to choose the safest drug that is most likely to succeed.<br><br>**Key steps/Expected course** *Anesthesia, drugs, blood, tubes & lines, recovery, rehab, nursing care, etc*<br>We use a local anesthetic to numb the skin. The team that puts the IV in is led by a nurse who does this every day and has a great deal of experience. They will set up a clean area to keep all the equipment sterile and this may take several minutes. We usually choose the inside of the elbow of the hand and. Arm you use less often. They will use a short larger needle to get into the vein and then place a very thin IV that will be inserted through that needle, and several inches will remain outside. They will put a clear sterile dressing over it that we hope lasts for the whole time the IV is in. Thy may put additional tape to anchor it, and we will have you wear a loose gauze wrap around all of it to protect it and make it less of a. nuisance for all other times.<br>The IV expert will look at you before they start, and they may ask for help from an ultrasound technician to find the best vein possible. That machine does not hurt at all but helps them find a good vein for the IV.

	After they are done they will likely take an X-ray to make sure it is in a good position.
Alternatives	**Alternate Treatments**<br>We can try high doses of antibiotics by mouth. There is not the same level of guarantee that we can get the highest level of antibiotics in your blood as we want. Also, if we use high doses of oral antibiotics for a long time, stomach aches and diarrhea can result.<br><br>**Course WITHOUT procedure**<br>Worsening infection, to include blood infection, and possible chronic infection in joint and/or permanent damage to joint
Risks	**Common/expected Side Effects (and work-arounds, like stool softeners, etc)**<br>The procedure hurts about as much as a blood test. Also, we have to teach you (your family) how to take good care of the IV so it doesn't fall out, get kinked or broken, clot off, or any of these things that may make it stop working too soon. You cannot be as active in sports/physical activities while we do this, and you cannot put the arm it is in under water. However you can go to school and do nearly anything with others as long as you protect the IV.<br><br>**"Major" & "Minor" complications**<br>Occasionally the IV stops working sooner than we expect and we will see how you are doing to decide if we need a new one or if we can pull it out at that time. Sometimes there is local irritation of the vein, causing it to be red, hard, and tender, what we call phlebitis, and we have to pull it out and either try a different site or go to only oral medicine. This could be from just the plastic irritating the vein or a local infection<br><br>Adverse Reactions/Toxicities of accompanying meds<br><br>As for the IV/PICC, nothing more than above. We will discuss the antibiotic separately<br><br>*Include remote but severe possibilities
Return	**Things for patient to watch for:**<br>When we show you how to give the medicine, you need to call us if you cannot get the medicine to go in. If the area gets red, swollen, is painful, or if you suddenly have a new fever, we will want to see you that day, or have you go to the Emergency Room if it is a weekend. We will give you numbers to be able to call us for advice.<br><br>**Things for patient to return for:**<br>As above

Procedure:_PICC

	**Standard/expected follow up plan:**<br>We will see you once a week and will set up those visits before you leave today. We will set up computer orders so you can go to the outpatient lab for tests which measure how the level of inflammation in your body is responding to treatment. You will go to the lab about an hour before our visits. After at least 2 weeks, we will work with you to decide when it is the right time to remove the IV. We do that in our outpatient clinic – it only takes a few minutes and does not hurt at all.
**Document**	**Surgical/Procedure Preoperative Counseling Note**<br>(a short, effective, appropriate EHR/EMR counselling note in this box)<br>PICC-Insertion:<br><br>3 January 2020, 14:55<br><br>Location – Pediatric Specialty Clinic procedure room<br><br>The parents and child were informed of the preference for prolonged intravenous access for the next 10 to 14 days and the benefits of using the IV route initially. These included guaranteed dosing and delivery of high levels of antibiotics to treat a left knee arthritis due to an MSSA. The risks of local bleeding, phlebitis, clots formation, local and systemic infection and premature PICC failure were all discussed with the parents who agreed to the procedure. The need to use this type of IV access, the discomfort of the procedure and the restrictions of activities during the time of the IV use as well as potential complications were discussed with the patient in the presence of both parents, who appeared to understand these risks well and gave his assent to the procedure.<br><br>**the following is the post-precedure note (not required for 16 Feb):**<br><br>The Basilic Vein was easily identified in the left antecubital fossa and the area was first cleansed with alcohol, infiltrated with 1.0cc of 1% lidocaine, then and cleansed with 3 chlorhexidine swabs ticks and a final alcohol wipe. A sterile field drape was placed and a XX French PICC line (XX Manufacturer) was inserted to the 34 cm mark as suggested by the manufacture based on the patients size and age. This was anchored and covered by a clear op-site, followed by additional taping to avoid movement of the line during future handling. A chest X-ray confirmed placement in the upper right superior vena cava above the right ventricle. Nursing staff then proceeded with instructions for home antibiotic administration and line care and maintenance as per standard protocols.

** Remember to allow questions from the patient

Procedure: Heel Laceration

2. Right Heel Laceration: ED-GMO

Patient is a 27-year-old female who presents to the ED after dropping a glass on the floor and stepping on the shards, resulting in a 4cm laceration on the bottom of her right heel. He has no other medical problems, and has medical decision-making capacity. Probing examination and imaging reveals no residual glass, or neuro/vascular compromise. You and your attending determine that she will require sutures, and you have been tasked with obtaining informed consent for the procedure.

a. Complete your "Informed Consent" study/prep card
b. Be prepared to role-play the role of the intern/physician in obtaining informed consent
c. Write an Informed Consent Note for the electronic medical record (EMR) AFTER the role play (hint: develop an outline for this case for your use)

<table>
<tr><th></th><th>Procedure (PARRQD)</th></tr>
<tr><td>Procedure</td><td>
Procedure/Intervention: (general description appropriate for patients (think 8th grade education)
A laceration, or cut, is an open wound through the skin. These can be due to many different causes and can come in many different forms. The depth, location, and cause of your wound, among multiple other factors (including your preference) effect the treatment choices we made today.

This procedure involves cleaning the cut on your heel and sewing the cut back together with stitches so that it can heal properly. We will give you a tetanus shot if you need one. You will be given medicine to numb the pain. We will remove any pieces of glass or damaged tissue we see in the cut. If anything is still bleeding we will stop the bleeding with stitches or a special cautery device. We will make sure there is no nerve, tendon, or other damage that may require a specialist or additional procedures. Once it is all cleaned up, we will sew the edges of the cut back together with about six to eight stitches. The stitches will be removed in about two weeks if everything heals up well. You may need to have additional procedures to close the wound.

Indication/Expected Benefits/likelihood of success
Laceration are the unavoidable result of injuries, disease, or surgery. It is impossible to totally remove the presence of a scar or laceration, yet plastic surgery in the future may improve the appearance and texture of laceration or scars. There is no guarantee of any outcome. The goal is improvement not perfection. It is important that you have realistic expectations.

The reason we need to clean and sew up your cut is to help it heal properly, remove any glass that may be in there, and try to prevent it from getting infected. We want to make sure there is no nerve, tendon, muscle, or other damage that may require a specialist or additional procedures. We want you to be able to move on your feet without difficulty so you can do all the things you were doing before it got cut.

Contraindications
Patient refusal.

Key steps/Expected course
1) We will numb the area of your wound with a local anesthetic to minimize the pain and make you as comfortable as possible.
2) Once the area is numb, we will carefully wash and clean your cut with a sterile solution and then place a sterile drape over the area.
3) We will clean up the edges of the cut and remove any glass we see.
</td></tr>
</table>

Procedure: Heel Laceration

	4) We will stop any bleeding we see with stitches, cautery, or other means.<br>5) We will make sure there is no nerve, tendon, muscle, or other damage that may require a specialist or additional procedures.<br>6) We will then carefully sew the edges together with stitches. This part will probably take about 15-20 minutes. We will give you more numbing medicine as we go along if you have any pain.<br>7) We will then put on some antibiotic ointment over the stitches.<br>8) We will then dress the wound to protect it while it heals.<br>9) We will then provide you with a set of crutches and teach you how to use them so that you can avoid putting any weight on it until it heals over the next two weeks.
**Alternatives**	**Alternate Treatments**<br>Alternative forms of treatment consist of not treating the skin laceration or scar or wound. This means trying to let the cut heal on its own, keeping the area as clean as possible and watching it carefully for any problems. With a cut on your foot like yours, cleaning and sewing up the wound is the best choice, since avoiding the area and keeping it clean is difficult. But if you do not want to have the wound stitched up, that is your choice.<br><br>**Course WITHOUT procedure**<br>Without cleaning and closing the wound, there is a much higher risk the wound will not heal well. The healing process will take much longer, and it may not completely close at all. You could have long-term problems using your foot without cleaning and closing the cut. Your open cut could also get infected which in some cases could travel deeply into your foot, which could put your leg at risk and indeed be life-threatening. Hospital admission and even emergency surgery would likely be necessary in such a situation.
**Risks**	**Common/expected Side Effects (and work-arounds, like stool softeners, etc)**<br>You may have some bruising and or swelling at the area of the cut. We will give you some pain medications, but there also may be some pain where the stitches were placed. You may get some constipation from the pain medications. We will also give you some stool softeners to help with that. You will have a scar. Your scar may be painful and may even affect the flexibility of your foot. The scar also may not look the way you want it to. You will need to protect the area, change the bandages, and keep weight off of the foot for two weeks if all goes well. We will special instructions and call-back numbers to us for all of this.<br><br>**“Major” & “Minor” complications**<br>There is a risk of bleeding that may require returning to the hospital. There may be an abnormal collection of blood or fluid in the cut that may need drainage or other additional procedures. You may have a reaction to the local anesthetic or the other antibiotics or pain medications we will be giving you for when you go home. You may develop an infection, poor healing, or reopening of the cut(s). An abscess or buildup of pus may form. It may require drainage or additional surgery. An infection into your foot may require more antibiotics, additional procedures, or even surgery. There may be a piece of glass or other debris left in the wound that we are unable to find. There could be numbness, damage to nerves, blood vessels, muscles, tendons, skin, and other structures surrounding the treatment area.<br><br>**Adverse Reactions/Toxicities of accompanying meds**<br>You may have an allergic reaction to the antibiotics. Very rarely there can be an accidental injection of the local anesthetic into a blood vessel. This may cause allergic reaction, seizures, irregular heartbeat, neurologic problems, heart attack, and even death.<br>*Include remote but severe possibilities
**Return**	**Things for patient to watch for:**

Procedure: Heel Laceration

	You may have some swelling, color changes, and bloody crusting on or around the wound for 2 or 3 days. This can be normal, and doesn't mean cut is not healing. At this time, scar tissue will be forming under the surface of the wound and your body will do the rest of the work of healing.<br><br>Take good care of your wound at home to help it heal quickly and reduce your chance of infection. While your wound is healing, avoid any activity that could cause your wound to reopen. Use common sense when it comes to activities. Also, avoid unnecessary, copious bacteria exposure–for example, swimming in an ocean or hot tub, or wearing shoes or gloves over a wound for a long period of time (which promotes bacterial growth).<br><br>You've been prescribed antibiotics, so take them as directed. Do not stop taking them just because you feel better. If you don't finish the course, you can breed resistant infections, which are harder to treat!<br><br>**Things for patient to return for:**<br>Call your doctor now, or seek immediate medical care in an emergency department if:<br>• Your wound is causing new pain, or the pain gets worse. Some pain is normal with a wound, but do not ignore pain that is getting worse instead of better. You could have an infection.<br>• The cut starts to bleed, and blood soaks through the bandage. Oozing small amounts of blood is normal.<br>• The skin near the cut is cold or pale or changes color.<br>• You have tingling, weakness, or numbness near the cut that we did not address during your visit.<br>• You have trouble moving the area near the cut that we did not address during the visit.<br>• You have increased pain, swelling, warmth, or redness around the cut, which could be a sign of infection.<br>• There are red streaks leading from the cut, or there is pus draining from the cut.<br>• You have a fever that you can't explain for another reason (like having a common cold).<br><br>**Standard/expected follow up plan:**<br>Keep the wound clean and dry for the first 12-24 hours. The wound has a bandage applied. You can remove the bandage after 12-24 hours at your convenience. After this, no further dressings are necessary. You can clean the area with a mild soap and water 2 times a day. Don't use hydrogen peroxide, iodine-based solutions, or alcohol, which can slow healing, and will probably be painful!<br><br>You may cover the wound with a thin layer of antibiotic ointment, such as bacitracin or neosporin. We will provide you with some for you to use. Be advised that some people can develop allergic skin rashes to topical antibiotics, especially neomycin (a component of neosporin) so you should avoid this if you have a known sensitivity. You can continue to apply the antibiotic twice daily until the wound scars and dries out, or the stitches are removed.<br><br>Some other important points are: Do not scratch, rub, or pick at the adhesive. Do not put tape directly over the adhesive. You can shower with a skin adhesive in place, but do not soak the area in water. Do not go swimming. Be sure to gently dry the area after it gets wet.<br><br>Your stitches be removed in about two weeks in our outpatient clinic. We will give you a time and place to return.<br><br>Do not bear weight directly on the wound until we see you again in two weeks to remove the stitches. We will provide some crutches and teach you how to use them safely before you leave.<br><br>Being treated in the emergency room is only one step in your treatment. Even if you feel better, you still need to go to all suggested follow-up appointments and take medicines exactly as directed. This will help you recover and help prevent future problems. Follow-up care is a key part of your treatment and safety. Be sure to make and go to all appointments, and call your doctor if things are not going as expected.
**Document**	**Surgical/Procedure Preoperative Counseling Note**

Procedure: Heel Laceration

	(a short, effective, appropriate EHR/EMR counseling note in this box)<br>The procedure, indications, steps, desired outcome, and projected recovery course of suture repair of a foot laceration in the emergency department was discussed with the patient in detail. In that context, I discussed and offered laceration repair and the patient agreed to proceed with the procedure. Specifically, the patient was counseled for risks including but not limited to bleeding, infection—including deep foot space infection; scarring; scar contracture; damage to deeper structures; retained foreign body; wound disruption; chronic pain; allergic or other reactions to local anesthetic or oral medications; delayed healing; additional procedures; or other unsatisfactory result.<br><br>Risks of uncommon major morbidity such as long term functional disability, limb loss, need for major surgery, severe medication reaction, and death were also discussed.<br><br>Alternatives such as letting the wound heal by secondary intention or watchful waiting was presented as an option to laceration repair. The risks of declining the procedure was discussed. Laceration repair was recommended, but I emphasized ultimately it was the patient's choice to make.<br><br>The patient verbalized clear understanding of the risks of the planned suture repair of a foot laceration, articulated a good understanding of the indications and basic steps of the procedure itself, its alternatives, follow-up plan, contingencies important patient compliance requirements, and follow-up guidance.<br><br>The patient asked questions throughout the informed discussion and all of the patient's questions were answered to the patient's satisfaction. The patient seemed satisfied with the care and the plan going forward.<br><br>The patient verbalized clear desire to proceed with the procedure at this time. The separate consent form was properly signed and witnessed by the patient and all relevant parties and placed in the patient's chart.<br><br>[Provider Name/Signed]

** Remember to allow questions from the patient

Procedure: Blood Transfusion

3. Blood Transfusion Anesthesia-Gen Surg - Rob Vietor

A 62-year-old male with significant peripheral arterial disease presents on the morning of a planned, elective right femoral-popliteal bypass. The attending surgeon and anesthesia team agree that the risk for intra-operative blood loss is high and are planning to be prepared to transfuse blood products intraoperatively if necessary. Your attending asks you to send a type and crossmatch, coordinate blood product availability with the blood bank, and obtain informed consent from the patient for a blood transfusion if needed.

a. Complete your "Informed Consent" study/prep card

b. Be prepared to role-play the role of the intern/physician in obtaining informed consent

c. Write an Informed Consent Note for the electronic medical record (EMR) AFTER the role play (hint: develop an outline for this case for your use)

<table>
<tr><th></th><th>Procedure (PARRQD)</th></tr>
<tr><td>Procedure</td><td>Procedure/Intervention: (general description appropriate for patients (think 8th grade education)

During the surgical procedure, if you have a lot of bleeding, we might use a catheter inserted into a vein to give you blood. This blood comes from pre-screened donors and has undergone testing for common diseases and infection. The purpose of this blood is to help deliver oxygen to your body and keep your organs functioning normally.

Indication/Expected Benefits/likelihood of success

If you experience too much blood loss during surgery, we may need to give you more blood to keep your organs healthy. The goal of a blood transfusion is to increase the amount of blood in your body by replacing the blood that you have lost during surgery. The new blood will help deliver oxygen throughout your body and help prevent you from having injuries to your heart, lungs, kidneys or brain. If we determine that a blood transfusion is necessary, we are very confident that we will improve the oxygen delivery to your organs.

Contraindications

Patient refusal

Key steps/Expected course
1) You will have a catheter placed in a vein prior to surgery
2) While you are already under anesthesia, we will connect special tubing to the catheter
3) The special tubing has a filter and a heater to keep the blood safe and warm
4) We will verify that the donated blood type is a match for your blood
5) We connect the donated blood to the other end of the tubing
6) We then drip the blood through the tubing with the filter and the heater
7) We will closely monitor you for complications or adverse reactions
8) Your blood levels will increase and your organs will receive more oxygen</td></tr>
</table>

Procedure: Blood Transfusion

Alternatives	**Alternate Treatments**<br><br>If you refuse blood transfusion we can consider a cell saver which suctions up the blood as you bleed, washes it and then allows us to return it to your body. We can also do autologous transfusion where we take blood from you at the start of surgery, dilute your remaining blood with saline, then give the undiluted blood after you have lost too much of the diluted blood. Both of these techniques are less likely to give us enough blood to replace your losses and have additional complications.<br><br>**Course WITHOUT procedure**<br><br>Without the blood transfusion you will be at higher risk for organ damage such as stroke, heart attack or kidney injury. We do not know the exact amount of blood that a patient can lose and still get enough blood and oxygen to their organs but we monitor things like your heart rate, blood pressure and lab results to determine the best point to give you blood to balance the risk of transfusion with the risk of not performing a transfusion.
Risks	**Common/expected Side Effects (and work-arounds, like stool softeners, etc)**<br><br>Common side effects include: fever, chills and itching. For these we can slow the transfusion or give you anti-itching or anti-fever medication.<br><br>**“Major” & “Minor” complications**<br><br>Major complications include infections from the blood products (HIV, bacteria, viruses, parasites, Hepatitis, West Nile Virus), acute immune hemolytic reaction, heart failure, high potassium, low calcium, bleeding, acute lung injury, stroke, heart attack and/or death.<br><br>**Adverse Reactions/Toxicities of accompanying meds**<br><br>We will not typically give you additional medications with the blood transfusion unless we need to treat one of the above side effects. Any additional medications will target your specific complication.<br><br>*Include remote but severe possibilities
Return	**Things for patient to watch for:**<br><br>Most complications from the blood will occur while you are in the hospital or before you are discharged home. That said, you should watch for yellowing of your skin, fevers, bleeding, dark urine, chest pain and shortness of breath after surgery.<br><br>**Things for patient to return for:**

	Notify your doctor or return to the hospital for yellowing of your skin, fevers, bleeding, chest pain, dark urine and shortness of breath after surgery<br><br>**Standard/expected follow up plan:**<br><br>You will likely have additional labs drawn after surgery to make sure your blood levels are OK and your bleeding is controlled. We will visit you each day after surgery while you are in the hospital and then you can return for your scheduled post-operative appointment with the surgeon.
**Document**	**Surgical/Procedure Preoperative Counseling Note**<br>(a short, effective, appropriate EHR/EMR counseling note in this box)<br><br>Patient is a 62-year-old male with significant peripheral arterial disease presenting on the morning of a planned, elective right femoral-popliteal bypass. Given his medical history and high likelihood of significant blood loss during surgery, it is likely that he will require intraoperative blood transfusion. We reviewed the advantages and disadvantages of blood transfusion and the alternatives. We discussed the conduct of and risks associated with blood transfusion to include fever, chills, itching, infections, acute immune hemolytic reaction, heart failure, electrolyte abnormalities, bleeding and acute lung injury. The patient verbalized understanding of the above and wishes to proceed with blood transfusion if indicated. All questions answered and all concerns addressed.

** Remember to allow questions from the patient

Procedure: subcutaneous mass removal

4. Subcutaneous Mass Removal: Gen Surgery Jared Antevil

A healthy 23-year-old male presents to the General Surgery clinic for removal of a 2cm subcutaneous soft tissue mass on his upper back. His history, physical exam, and radiographic studies are consistent with benign lipoma. He has no significant medical history and is on no medications. He has no history of substance use. Your senior resident plans to excise the mass - with your assistance - in the minor procedure room in the surgery clinic with local anesthetic, and asks you to obtain informed consent for the procedure while the room is being prepped.

a. Complete your "Informed Consent" study/prep card

b. Be prepared to role-play the role of the intern/physician in obtaining informed consent

c. Write an Informed Consent Note for the electronic medical record (EMR) AFTER the role play (hint: develop an outline for this case for your use)

	Procedure (PARRQD)
Procedure	**Procedure/Intervention: (general description appropriate for patients (think 8th grade education)**<br>After giving you a local numbing medication we will make an incision in the skin and tissues of your back overlying the mass. We will then remove the mass, send it for testing to determine its exact cause, and close the incision with dissolvable sutures.<br><br>**Indication/Expected Benefits/likelihood of success**<br>The goals of this procedure are to remove the mass from your back in its entirety and to send it for diagnostic testing (to determine the cause of the mass) – we fully expect that we can achieve these results with a very high likelihood of success.<br><br>**Contraindications**<br>None<br><br>**Key steps/Expected course** *Anesthesia, drugs, blood, tubes & lines, recovery, rehab, nursing care, etc*<br>1) We will clean the area to create a "sterile field" for the surgery<br>2) We will inject a local medication to numb the area over the mass<br>3) We will remove the mass and send it for testing (which will take an estimated 7-10 days for results)<br>4) After irrigating the wound with sterile fluid, we will close the wound with dissolvable sutures and place a dressing
Alternatives	**Alternate Treatments**<br>Instead of removing the mass from your back we could either observe it or attempt a biopsy (sample it with a needle to determine its cause)<br><br>**Course WITHOUT procedure**<br>If we do not remove this mass, it will remain in place, may enlarge, and could lead to later problems. In addition there is a very small risk this mass could be cancerous, which could be life-threatening if not removed.

Procedure: subcutaneous mass removal

Risks	**Common/expected Side Effects (and work-arounds, like stool softeners, etc)**<br><br>Side effects from this procedure include local pain/discomfort/irritation and fluid build-up beneath the incision (seroma)<br><br>**“Major” & “Minor” complications**<br>Possible complications include bleeding, infection, recurrence (return) of a mass after removal, chronic pain from surgery<br><br>**Adverse Reactions/Toxicities of accompanying meds**<br>Not applicable<br><br>*Include remote but severe possibilities
Return	**Things for patient to watch for:**<br>Watch for signs of infection (redness over incision, fevers/chills, drainage from the wound) or bleeding (increasing swelling beneath the incision or ongoing bloody drainage from the incision).<br><br>**Things for patient to return for:**<br>Return for signs of bleeding or infection<br><br>**Standard/expected follow up plan:**<br>You will have an appointment to return in approximately two weeks. At that visit we will examine your wound and review the “pathology” report from the examination of your mass under a microscope.
Document	**Surgical/Procedure Preoperative Counseling Note**<br>(a short, effective, appropriate EHR/EMR counseling note in this box)<br><br>This is a 23 year-old male with a 2cm subcutaneous soft tissue mass on his back, clinically most consistent with a lipoma. We discussed options of removal or observation (with or without biopsy). We reviewed the advantages and disadvantages of both alternatives. We discussed the conduct, recovery and risks associated with removal of a soft tissue mass, to include bleeding, infection, hematoma, seroma, chronic pain, and mass recurrence. The patient verbalized understanding of the above and wishes to proceed with surgical removal. All questions answered and all concerns addressed.

** Remember to allow questions from the patient

Procedure: Vasectomy

5. Vasectomy: FAM MED Anna T. Wiley

Patient is a 48yo M who presents to the family medicine clinic for pre-procedural counseling for a scheduled vasectomy. He has three children with his wife of twenty-two years, ages 17, 13, and 9, and they do not desire additional children. He has no significant past medical history and takes no medications. He had his wisdom teeth removed thirty years ago. He has medical decision-making capacity. You are tasked with obtaining informed consent for the procedure.

a. Complete your "Informed Consent" study/prep card
b. Be prepared to role-play the role of the intern/physician in obtaining informed consent
c. Write an Informed Consent Note for the electronic medical record (EMR) AFTER the role play
(hint: develop an outline for this case for your use)

	Procedure (PARRQD)
**Procedure**	**Procedure/Intervention: (general description appropriate for patients (think 8th grade education)**<br>After shaving and cleaning the scrotum with an antibiotic solution, you will receive a numbing injection in the scrotum. Then, we will make a small incision into the scrotum and remove part of the vas deferens, which interrupts the flow of sperm and causes sterilization.<br><br>**Indication/Expected Benefits/likelihood of success**<br>Vasectomies are more than 99% effective at preventing pregnancy and is a one-time procedure that does not require general anesthesia and has a low risk of complications.<br><br>**Contraindications**<br>There are no absolute contraindications to this procedure, but contraindications to an outpatient vasectomy include history of coagulopathy, previous scrotal surgery, infection at the surgical site, and testicular cancer. Your physician may also decline to perform this procedure if there are concerns for regret (e.g. patient is young, spouse is currently pregnant, etc.).<br><br>**Key steps/Expected course** *Anesthesia, drugs, blood, tubes & lines, recovery, rehab, nursing care, etc.*<br>1. We will shave the scrotum, if necessary, and then clean it with an antibiotic solution.<br>2. We will identify the vas deferens and inject a local numbing agent.<br>3. We will open the skin using a sharp dissector and inject more numbing agent around the vas deferens.<br>4. We will isolate the vas deferens through the incision and remove 1 cm of the vas deferens<br>5. We will cauterize the ends and allow them to retract back into their original position.<br>6. We will repeat the same procedure on the other side.<br>7. We will allow the skin incision to close on its own. There is no need for stitches.<br>8. We will apply vasaline and gauze to the surgical site.

Procedure: Vasectomy

Alternatives	**Alternate Treatments**<br><br>Other methods of contraception can be discussed with you and your partner, including condoms, spermicide, oral contraceptive pills, IUD, depot injections, or tubal ligation. An alternative to the in-office vasectomy would be a vasectomy in the operating room under general anesthesia.<br><br>**Course WITHOUT procedure**<br><br>If you choose not to undergo a vasectomy, you will not be sterilized and your partner may become pregnant if you or your partner do not elect to use another form of contraception.
Risks	**Common/expected Side Effects (and work-arounds, like stool softeners, etc.)**<br>Scrotal pain is very common after this procedure. Keep the scrotum elevated for at least one day after the procedure and apply ice packs frequently. You can return to non-strenuous work 48 hours after the procedure and slowly return to strenuous activity after two weeks. Refrain from sexual intercourse and ejaculation for one week post-procedure, as this can also aggravate scrotal pain. Continue to use the jock strap for one week post-procedure.<br><br>**“Major” & “Minor” complications**<br>Complications are rare, but include infection, the formation of a hematoma (collection of blood), or granuloma (scar tissue). There is a 0.4% risk of failure of a vasectomy and a 1 in 2000 risk of pregnancy despite a successful vasectomy. There is also a risk of remorse or remorse in your partner, as well as the rare risk of anesthetic reaction and permanent pain.<br><br>**Adverse Reactions/Toxicities of accompanying meds**<br>Preservatives found in lidocaine, the local numbing agent, may cause an adverse reaction.<br><br>*Include remote but severe possibilities
Return	**Things for patient to watch for:**<br>Watch for signs of infection: blood or pus draining from the surgical site, fever of >100.4F, worsening pain, swelling, or warmth.<br><br>**Things for patient to return for:**<br>Return if you have signs of an infection, bleeding, or hematoma greater than softball size.<br><br>**Standard/expected follow up plan:**<br>You will return for semen analysis in 12 weeks. Please have at least 20 ejaculations prior to the analysis and abstain from ejaculation for 2-3 days prior to the collection of semen.

Procedure: Vasectomy

Document	**Surgical/Procedure Preoperative Counseling Note**<br>(a short, effective, appropriate EHR/EMR counseling note in this box)<br><br>Patient is a 48yo M desiring sterilization, presenting to clinic with his wife to discuss the option of a vasectomy. This is an elective procedure, and we reviewed risks and benefits of the procedure as well as alternative methods of contraception. We discussed that although a vasectomy can be reversed, it is considered a permanent method of sterilization and should not be considered if the patient may desire future children. Risks include infection, bleeding, granuloma formation, procedure failure, and remorse. We reviewed steps of the procedure with the use of an anatomy model. The patient verbalized their understanding of the procedure and risks involved and wishes to proceed with a vasectomy. All questions and concerns were addressed. Pre-operative counseling was completed and patient was scheduled for an outpatient vasectomy.

** Remember to allow questions from the patient

Procedure: Platelet rich plasma injection

6. Platelet Rich Plasma Injection: FamM

Patient is a 20yo F who presents to the family medicine clinic for a right knee PRP injection. She is a collegiate basketball player with a yearlong history of patellar tendonitis ("jumper's knee") that has been refractory to physical therapy and corticosteroid injections. She has no significant past medical history and takes no medications. She had an appendectomy twelve years ago. She has medical decision-making capacity. You are tasked with obtaining informed consent for the procedure.

a. Complete your "Informed Consent" study/prep card
b. Be prepared to role-play the role of the intern/physician in obtaining informed consent
c. Write an Informed Consent Note for the electronic medical record (EMR) AFTER the role play (hint: develop an outline for this case for your use)

	Procedure (PARRQD)
**Procedure**	**Procedure/Intervention: (general description appropriate for patients (think 8th grade education)**<br>First, we will perform a venipuncture (blood draw) and then spin down your tube of blood via centrifugation to separate your platelets from other blood components and create "platelet-rich plasma". Then, we will inject your own platelet-rich plasma into the affected joint (right knee).<br><br>**Indication/Expected Benefits/likelihood of success**<br>PRP injections are not FDA approved since patient's platelet-rich plasma is not considered a drug. However, PRP injections have been shown to be effective in the treatment of joint, ligament, and tendon injuries, post-operative healing, and osteoarthritis. Limited studies demonstrate a significant reduction in pain and improvement in function after PRP injections in patients with chronic tendon problems.<br><br>**Contraindications**<br>Contraindications to PRP injections include thrombocytopenia (low platelet count) or other platelet dysfunction, blood infection/septicemia, chronic infection (Hep C, HIV/AIDS, etc.), local infection at the site of injection, and cancer with bone or blood/hematologic involvement.<br><br>**Key steps/Expected course** Anesthesia, drugs, blood, tubes & lines, recovery, rehab, nursing care, etc<br>1. We will draw 50mL of blood from an arm vein under sterile technique (show syringe and tube).<br>2. We will use a centrifuge machine to spin down your blood and isolate platelet-rich plasma.<br>3. We will use ultrasound (recording non-invasive sound waves) to visualize the area of injury.<br>4. We will inject numbing medicine/local anesthesia at the site of the intended PRP injection under sterile technique, and then inject your PRP.<br>5. We will apply a dressing to the injection site.
**Alternatives**	**Alternate Treatments**<br>This is an elective procedure. If you elect not to undergo PRP injection, we can consider alternative routes of treatment such as conservative management with pain medications like ibuprofen and others /analgesics and continued physical therapy, knee bracing, dry needling, or orthopedic consultation for consideration of operative management.<br><br>**Course WITHOUT procedure**<br><br>If you choose to not undergo PRP injection, you will continue to experience right knee pain without alternative treatment.

Procedure: Platelet rich plasma injection

**Risks**	**Common/expected Side Effects (and work-arounds, like stool softeners, etc)**<br>Side effects of PRP injection are very rare, the most common being pain at the site of injection.<br><br>**"Major" & "Minor" complications**<br>Complications are extremely rare but may include pain at the site of injection and local infection at the site of injection.<br><br>**Adverse Reactions/Toxicities of accompanying meds**<br>Because we are injecting platelet-rich plasma from your blood, there is no risk of an allergic reaction. Preservatives found in lidocaine, the local numbing agent, may cause an adverse reaction.<br><br>*Include remote but severe possibilities
**Return**	**Things for patient to watch for:**<br>Watch for signs of infection: blood or pus draining from the injection site, fever of >100.4F, worsening pain, swelling, or warmth.<br><br>**Things for patient to return for:**<br>Return if you have signs of an infection or bleeding.<br><br>**Standard/expected follow up plan:**<br>You will return for follow up in 8 weeks to assess your response to the PRP injection.
**Document**	**Surgical/Procedure Preoperative Counseling Note**<br>(a short, effective, appropriate EHR/EMR counseling note in this box)<br><br>Patient is a 20yo F with h/o right patellar tendonitis refractory to physical therapy, analgesia, and corticosteroid injections, who presents for a right knee PRP injection. We reviewed options of PRP injection or another trial of physical therapy, dry needling, or surgical consultation and the advantages and disadvantages of these options. We discussed risks of pain at the injection site and local infection. We reviewed steps of the procedure. The patient verbalized their understanding of the procedure and risks involved and wishes to proceed with right knee PRP injection. All questions and concerns were addressed.

** Remember to allow questions from the patient

7. Lumbar Puncture: Neuro-ED-Med/Psy Jed Mangal

A 27 y/o woman developed the sudden onset of a headache that peaked to a 10/10 intensity over the course of a few seconds while doing cross training today. She developed nausea and vomiting shortly after. Her partner brought her to the ED where she continues to c/o a persistent severe holocranial headache. On exam, she has mild neck stiffness. Her right pupil is 6mm in the light and 6mm in the dark. Her left pupil is 3 mm in the light and 5mm in the dark. You have concern for a SAH and recommend she undergo a lumbar puncture. She has no other significant medical history. She has been engaged in the evaluation of her difficulties and is able to engage in shared decision making.

a. Complete your "Informed Consent" study/prep card
b. Be prepared to role-play the role of the intern/physician in obtaining informed consent
c. Write an Informed Consent Note for the electronic medical record (EMR)
AFTER the role play (hint: develop an outline for this case for your use

	**Procedure (PARRQD)**
**Procedure**	**Procedure/Intervention: (general description appropriate for patients (think 8th grade education)**<br>Our team has reason to believe that you may have a life-threatening bleed in the space around your brain. Although the images of your brain did not show bleeding, we are still concerned for bleeding that might not show up in the pictures due to how long you have had symptoms. Our next best test is a lumbar puncture, which involves inserting a small needle to sample the fluid which also surrounds the brain in order to examine the fluid and measure the amount of blood.<br><br>**Indication/Expected Benefits/likelihood of success**<br>-Diagnostic benefit: meningitis, SAH, MS, malignancies<br>-Measurement of CSF pressure<br>*We fully expect that this procedure can be performed with a high likelihood of success<br><br>**Contraindications**<br>1. Increased intracranial pressure<br>2. Infection at the puncture site<br>3. Coagulopathy<br>4. Anatomical variations requiring fluoroscopy (surgery, fusions, etc)<br><br>**Key steps/Expected course**<br>1. The skin at the base of your spine will be sterilized and draped to prevent infection<br>2. A site at which it is possible to pass a small needle into a safe location around your spine will be identified and marked<br>3. Numbing medicine will be used to reduce any discomfort that the procedure may cause<br>4. A small needle will be inserted into the space around your spine. Fluid pressure will be measured, the color will be noted, and the fluid will be collected to be sent to the lab.<br>5. We will remove the needle and placed a small dressing.<br>6. You asked to be lie flat for a period of time to prevent worsening headache
**Alternatives**	**Alternate Treatments**<br>We can proceed without this study and continue to evaluate the cause of your headache with other imaging that may delay diagnosis.<br>**Course WITHOUT procedure**

	Our team worries that we could be missing a serious bleed that might require surgical intervention. It is possible that delaying diagnosis could lead to worsening symptoms and death.
Risks	**Common/expected Side Effects (and work-arounds, like stool softeners, etc)**<br>It is common to have a headache following the procedure, especially if a large amount of fluid removed. Only the minimum amount of fluid to conduct diagnostic studies will be removed in order to prevent headache. You also will be asked to remain flat for a period of time following the procedure in order to prevent this problem.<br><br>It is possible to develop a small leak of fluid following the procedure however a special needle which spreads the fibers of the sac around your spine will be used instead of a cutting needle. This change helps prevent leaks.<br><br>**“Major” & “Minor” complications**<br>Introducing bacteria into the spinal column is possible and could cause infection. Sterile technique used for the entirety of the procedure to prevent this complication.<br><br>Anytime the needle is inserted, there is a chance of causing some bleeding if blood vessels are damaged. The procedure will be conducted at a safe location as identified by her anatomy and will monitor for bleeding.<br><br>Injury to the spinal cord is possible if the needle is not carefully advanced, but all attempts will be made to insert the spinal needle to the minimum depth in order to collect spinal fluid.<br><br>Herniation of the brain stem is possible and a life-threatening complication of this procedure, however your CT scan did not show evidence of increased pressure inside the skull which lowers the risk of this complication significantly.<br><br>**Adverse Reactions/Toxicities of accompanying meds** *Include remote but severe possibilities<br>N/A
Return	**Things for patient to watch for:**<br>Following the procedure, you will be given results of the study. If bleeding is identified, consultation with neurosurgery or interventional radiology might be sought. If bleeding is not identified or there are other laboratory findings, we will discuss next steps with you.<br><br>Following the procedure, please monitor for increasing headaches or pain. Pay particular attention to any changes in your (or your family member's) wakefulness, thinking, vision, strength, or speech. Please notify member of our team if any of these problems are Identified
Document	**Surgical/Procedure Preoperative Counseling Note**<br>(a short, effective, appropriate EHR/EMR counseling note in this box)<br>Lumbar Puncture Procedure note<br><br>14 FEB 2022, 14:55 Location – Emergency Department<br><br>The patient was informed of the diagnostic benefit of performing a lumbar puncture to rule out a subarachnoid hemorrhage. Risk of the procedure including postprocedure headache, back pain, infection, hemorrhage, and brainstem herniation, and paralysis were described to the patient. After discussion of the risks and benefits of the

	procedure, informed consent was obtained and the patient agreed to undergo the procedure. The patient was alert, engaged in discussion, and demonstrated capacity to engage in shared decision making.<br><br>Post-procedure note:<br><br>Prior to starting the procedure a timeout was conducted during which the procedure, the patient, and the location of the procedure were confirmed. The L3-L4 interspace was identified using anatomic landmarks and marked with a skin marking pen. The procedure site was prepared using sterile technique, using ChloraPrep to prepare the skin, and the local drape was placed. 1% lidocaine inserted with a 22-gauge hypodermic needle was used to anesthetize down to the level of the vertebrae. A spinal needle was inserted into the interspace at an advanced slowly, periodically removing the stylette to visualize CSF. Once CSF was visualized, the needle was not advanced any further. A manometer was attached and CSF pressure was measured at XX centimeters of water. Clear CSF was visualized and collected sequentially in tubes 1 through 4. The stylette was replaced and the spinal needle was removed with minimal blood loss. A local Tegaderm dressing was applied and the site appeared hemostatic.<br><br>The patient was instructed to lie flat for 1-2 hours to prevent post procedure headache.

** Remember to allow questions from the patient
