# Supplementary material for: Obtaining and Documenting Informed Consent: An Advanced UME Cross-Specialty, Role-Playing Skill Builder
Source: MedEdPORTAL. 2026 Mar 3;22:11580. doi: 10.15766/mep_2374-8265.11580 (PMC12956033; doi:10.15766/mep_2374-8265.11580)
Supplement: Supplementary file 1 — Course Syllabus.docxPrereadings.pdfStatPearls Article.pdfADMSEP eModule folderClinical Vignettes.pdfRubric.pdfMARRQD, PARRQD Templates.docxOrientation.pptxObserver-Scribe Template.docxVignette Answers.pdf [file mep_2374-8265.11580-s001.zip › _Educational Summary Report_11580.pdf]

MedEdPORTAL
THE AAMC JOURNAL OF TEACHING AND LEARNING RESOURCES

Original Publication
Open Access

Obtaining and Documenting Informed Consent: An Advanced UME Cross-Specialty, Role-Playing Skill Builder

Radhika Tyagi, MD*, Elizabeth A. Greene, MD, Louis Lachman, MD, Derrick A. Hamaoka, MD, Philip S. Mullenix, MD, JD, Jed Mangal, MD, Margaret M. Swanberg, DO, Emily G. Diana, MD, Kelly L. Cozza, MD

*Corresponding author: radhika.tyagi.mil@health.mil

Abstract

Introduction: Informed consent is a critical skill in medical practice, yet deficiencies in training are common. Despite its recognition as a core Entrustable Professional Activity for medical students, many report inadequate exposure to formal informed consent education. This study evaluates an enhanced, postclerkship course designed to improve students' proficiency in obtaining and documenting informed consent across multiple specialties. **Methods:** The course, delivered to 171 fourth-year medical students in academic year (AY) 2022 and 167 in AY 2023, involved online preparatory work and a 4-hour synchronous session combining lecture, faculty-led role-playing, and peer feedback. Students were tasked with preparing informed consent scenarios involving medications and procedures, then practicing in small groups with faculty guidance. Pre- and postcourse satisfaction surveys and knowledge quizzes were administered. **Results:** The course significantly increased students' reported confidence in obtaining informed consent. In addition, in AY 2023, the course was rated highest among the 5 presented that week when students were asked, "Which skills do you now feel better prepared to perform?" Qualitative feedback highlighted the role-playing sessions as the course's most valuable component. **Discussion:** This course successfully enhanced students' ability and confidence in obtaining informed consent in a low-stakes, supportive environment. The inclusion of multiple specialties and the opportunity to practice documentation addresses gaps identified in prior curricula. Future iterations could optimize session timing and consider offering earlier training, prior to clinical clerkships. This model could also be adapted for longitudinal assessment of informed consent proficiency in medical trainees across undergraduate and graduate medical education.

Keywords
Communication Skills, Provider-Patient Relationship, Quality Improvement/Patient Safety, Case-Based Learning, Clinical/Procedural Skills Training, Simulation

Educational Objectives

By the end of this activity, learners will be able to:

1. Utilize an essential steps rubric for obtaining and documenting informed consent for medications and procedures.
2. Facilitate a shared decision-making discussion about a proposed treatment plan via supervised role-play.
3. Propose evidence-based therapeutic options for assigned vignette patients.
4. Assess and discuss self and peer performance in obtaining and documenting informed consent.

Citation:
Tyagi R, Greene EA, Lachman L, et al. Obtaining and documenting informed consent: an advanced UME cross-specialty, role-playing skill builder. *MedEdPORTAL.* 2026;22:11580.
https://doi.org/10.15766/mep_2374-8265.11580

Introduction

Obtaining and documenting informed consent is an essential, universal component of medical practice; by creating trust and clarifying understanding, obtaining and documenting informed consent enhances use of the medical system and treatment adherence.[1] As such, informed consent has been identified by the AAMC as one of the core Entrustable Professional Activities (EPAs), or a skill that medical students should gain proficiency in prior to starting residency.[2] Despite the designation of informed consent as an EPA, a standardized curriculum for teaching informed consent does not exist. Deficiencies in both the quality and quantity of informed consent education are frequently cited by medical students and residents. One survey of medical students and practitioners found that only

Copyright © 2026 Tyagi et al. This is an open-access publication distributed under the terms of the Creative Commons Attribution license.

60% of respondents reported adequate informed consent training.[3] Another survey of medical students at Stanford University School of Medicine found that 75% of respondents reported receiving no formal informed consent training, and 15% reported never having witnessed an informed consent discussion.[4] The importance of informed consent education is apparent to medical students, who indicate in surveys the need for bolstered informed consent education in the medical school curriculum.[5]

In 2017, Diana and colleagues described a weekly role-playing exercise, Obtaining Informed Consent for Psychotropic Medications, utilized during the 5-week core psychiatry clerkship rotation at Uniformed Services University (USUHS).[6] Routine, anonymous postclerkship student surveys regarding that experience demonstrated overall satisfaction with the course and improved confidence in obtaining informed consent. By academic year (AY) 2019, student feedback indicated a need for a more advanced informed consent curriculum. For AY 2020, in addition to the psychiatry-specific, clerkship-level weekly formative informed consent medication role-play, a new half-day postclerkship course was developed to provide low-stakes training in both obtaining and documenting informed consent. This advanced course includes a review of the Essential Elements of Communication, new rubrics and vignettes for both medications and procedures across several medical-surgical specialties, peer and expert faculty observations, and formative feedback. Course directors created the role-playing Procedure/Medication, Alternatives, Risks, Return Precautions, Question, Document (PARRQD/MARRQD) model to better assist junior trainees in obtaining and documenting informed consent.

The specifics of the curriculum and outcome data on impact and satisfaction are provided below. It is expected that utilizing an enhanced and advanced course on obtaining and documenting informed consent after the completion of core clerkships will provide an effective opportunity to hone an essential clinical skill in a simulated environment prior to beginning advanced clinical rotations. Such a course also offers institutions the opportunity to document and meet GME expectations for this core EPA skill for PGY-1 students. While literature can be found on the effectiveness of informed consent courses delivered to medical professionals in various modalities, the curriculum outlined here is, to our knowledge, the first of its kind to offer broad training in both obtaining and documenting informed consent spanning multiple medical specialties, with an intended audience of medical students.

Methods

We designed the Obtaining and Documenting Informed Consent course as a role-play experience for students in the immediate postclerkship period as part of the USUHS Bench to Bedside and Beyond (B3) 6-week postclerkship curriculum aimed at reinforcing and advancing clinical competencies. We developed this informed consent course following Kern's Six-Step Model of Curriculum Development, beginning with problem identification and needs assessment.[7] We selected independent preparatory work (prework) followed by small-group role-play as the educational strategy, placing the emphasis on low-stakes skill development during instructional time. In AY 2022, the 2 course directors (Kelly L. Cozza and Philip S. Mullinex) developed new role-play scenarios and rubrics for this course, including 5 medication scenarios and 5 procedure scenarios. Upon completion of the 2022 course, we utilized informal student and faculty feedback to develop a second iteration with the inclusion of more procedure cases. Faculty developed more procedural vignettes, for a total of 3 medications and 7 procedures in AY 2023. The USUHS Human Research Protections Program determined that the development of this curriculum and analysis of the results of pre- and postcourse surveys is considered research not involving human subjects and therefore did not require IRB approval.

In February 2022, we administered the first iteration of this new half-day course in a synchronous, yet virtual format to a class of 171 students. We delivered the 2023 iteration of the course synchronously and in-person to 167 postclerkship students, with updated instructions suited to an in-person environment. Our course directors recruited 9–12 physician faculty members spanning multiple specialties to facilitate small-group role-play. We provided students with a course syllabus, which included the educational objectives, required prework, and a breakdown of the schedule for the half-day course (Appendix A). The prework provided to students included the Essential Elements of Communication (Appendix B), an online, freely accessible article about informed consent (Appendix C), and an online, freely accessible and interactive informed consent e-module (Appendix D).[8,9] Additionally, we tasked students with completing an informed consent template using the PARRQD/MARRQD model for 1 medication or procedure vignette assigned prior to the course; clinical vignettes (Appendix D), a rubric (Appendix F), and templates (Appendix G) were provided.

For both new iterations, students completed the same anonymous, formative 2-question knowledge quiz immediately before and after the half-day course (results reported in Table 2).

Copyright © 2026 Tyagi et al. This is an open-access publication distributed under the terms of the Creative Commons Attribution license.

A 30-minute overview on the processes of obtaining and documenting informed consent and an orientation to the exercise by the course directors (Appendix H) was followed by assignment of students to small groups (9–12 students per group), each with a faculty preceptor. Individual groups rotated through the roles of medical provider, patient, and observer/scribe while they practiced the informed consent scenarios (Appendix E, Appendix I). At the end of the 2.5-hour group sessions, group participants reflected on the exercise with their preceptor and developed a list of practical information gained (clinical pearls) about obtaining and documenting informed consent. The course concluded with all students and faculty reconvening as a large group to discuss feedback, share pearls, and ask questions for 30–45 minutes.

Faculty-developed answer keys for the informed consent PARRQD/MARRQD cases were provided following the conclusion of the course (Appendix J). The in-person AY 2023 iteration small-group sessions allowed faculty to observe groups of 3 students simultaneously, requiring considerably less time than the virtual AY 2022 iteration. For AY 2022–2023, the USUHS School of Medicine pre– and post–6-week course satisfaction surveys on preparedness and confidence asked students a question about their sense of preparedness/confidence in obtaining informed consent (Table 1), and in AY 2023, they also asked students to indicate which skills (from among a list of applicable skills) overall they felt better prepared to perform each week before and after participating in the 6-week course (Figure 1 and Figure 2).

Results

During the 6-week postclerkship course, students were required to complete pre- and postcourse feedback surveys about the quality and utility of that week's courses. In AY 2022, 171 students participated in the informed consent course. On both the pre- and postcourse surveys, the response rate was 95.9% ($n = 164$). In AY 2023, 167 students participated in the informed consent course. The response rate was 100% ($n = 167$) for the precourse survey and 90.4% ($n = 151$) for the postcourse survey. Overall, the survey responses indicated that the informed consent course was successful in increasing medical students' confidence in obtaining informed consent, with a 35% overall increase in students expressing that they felt prepared to obtain informed consent after completing the course (Table 1). Prior to taking the informed consent course, 38 (23%) of 167 students answered that, of all the skills being taught that week, obtaining informed consent was the skill they felt most prepared to perform (Figure 1), which increased to 134 (89%) of 151 respondents postcourse (Figure 2).

In addition, students completed a precourse/postcourse 2-question knowledge quiz about the process and content of obtaining and documenting informed consent. Quiz results demonstrated a small but significant difference between students' precourse and postcourse knowledge scores for both years combined (Table 2).

Written feedback was elicited from all students on the greatest strengths of the sessions they attended that week, and notably, 15 of the 17 comments specifically highlighted the informed consent course. These comments highlighted role-playing as a strength of the informed consent course. Of the 150 students who offered written feedback, only 10 indicated they had received adequate practice obtaining and documenting informed consent on clinical rotations.

Overall, student comments highlighted the utility of the informed consent course, although a few students noted they had been able to practice informed consent during clinical rotations.

Sample feedback responses are as follows:

- "I enjoyed the role playing of the consent small groups as well as the service-specific medical readiness session.

Table 1. Pre- and Postcourse Student Survey Confidence Results on Obtaining Informed Consent

	Response[a]			
Academic Year	Yes *n* (%)	Sort of *n* (%)	No *n* (%)	*P* vs Precourse
2022				
Precourse ($N = 164$)	78 (48)	73 (45)	13 (8)	
Postcourse ($N = 164$)	144 (88)	18 (11)	2 (1)	<.001
2023				
Precourse ($N = 167$)	91 (54)	66 (40)	10 (6)	
Postcourse ($N = 151$)	126 (83)	16 (11)	9 (6)	<.001
Total				
Precourse ($N = 331$)	169 (51)	139 (42)	23 (7)	
Postcourse ($N = 315$)	270 (86)	34 (11)	11 (3)	<.001

[a]Students selected options of *Yes*, *Sort of*, or *No* regarding sense of confidence/preparedness in obtaining informed consent.

Copyright © 2026 Tyagi et al. This is an open-access publication distributed under the terms of the Creative Commons Attribution license.

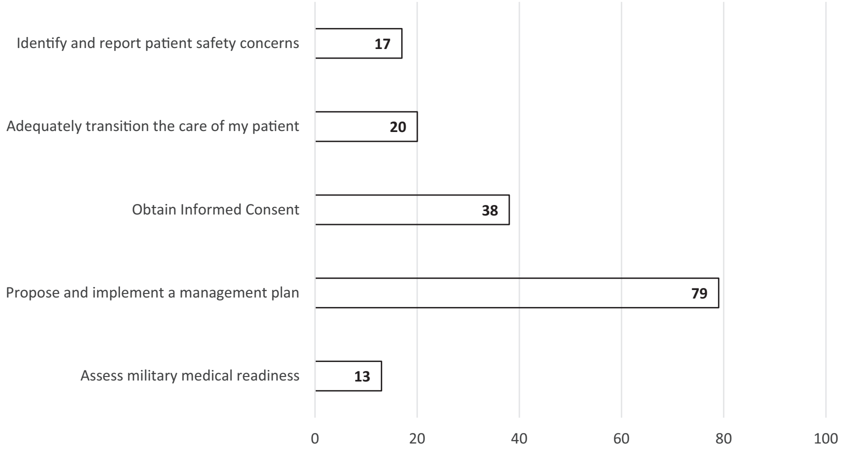

Figure 1. Academic year 2023 survey responses before students participated in the 6-week postclerkship course (N = 167). Values in bars are the number of students indicating preparedness for each skill in response to the question, "Which of these skills do you feel most prepared to perform (check all that apply)?"

Well balanced between active small groups, lectures, and panels."

- "Gave time to practice concepts such as informed consent and transitions of care that I saw during clerkship but hadn't gotten the opportunity to do so yet."
- "[S]mall group exercises for obtaining consent were valuable."
- "I believe the [informed consent] session would be far more beneficial at the start of clerkships—I did this almost daily on my surgery clerkships and therefore it did not alter my confidence level doing this session."
- "I was expected to perform IC on real patients and learned more from having to do it off the cuff than through a prepared 3-hour small group."

Discussion

This half-day course provides postclerkship medical students the opportunity to practice an essential skill in a low-stakes environment and receive feedback from both peers and experienced faculty. While its predecessor course was limited to obtaining informed consent solely for medications, without any content related to documentation, this new educational tool includes vignettes spanning multiple medical specialties, medications, procedures, and documentation. At our institution, many students do not have the opportunity to practice documenting the process of obtaining informed consent during medical school, and we suspect this may be a common phenomenon elsewhere. In a didactic session delivered to academic surgeons, one of the most common suggestions for improving the course was offering more pointers on informed consent documentation, highlighting the importance of developing this skill early in one's medical education.[10] Furthermore, this single educational session is adaptable to both virtual and in-person environments and can be easily tailored to longitudinal medical school and residency curricula across specialties.

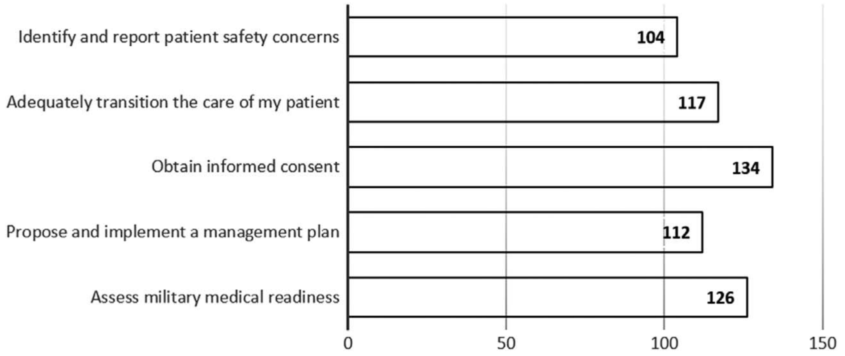

Figure 2. Academic year 2023 survey responses after students participated in the 6-week postclerkship course (N = 151). Values in bars are the number of students indicating preparedness for each skill in response to the question, "Which of these skills do you now feel better prepared to perform (check all that apply)?"

Copyright © 2026 Tyagi et al. This is an open-access publication distributed under the terms of the Creative Commons Attribution license.

Table 2. Student Pre- and Postcourse Knowledge Quiz Results on Process and Content of Obtaining and Documenting Informed Consent

	Precourse		Postcourse	
**Academic Year**	**Correct *n* (%)**	**Incorrect *n* (%)**	**Correct *n* (%)**	**Incorrect *n* (%)**
2022[a] ($N_{pre/post}$ = 164)	267 (81)	61 (19)	290 (88)	38 (12)
2023[b] (N_{pre} = 167; N_{post} = 151)	290 (87)	44 (13)	267 (88)	35 (12)
Total[c]	557 (84)	105 (16)	557 (88)	73 (12)

[a]Calculated from a total of 328 responses (2 questions per learner).
[b]Calculated from a total of 334 responses on precourse survey and 302 responses on postcourse survey (2 questions per learner).
[c]Calculated from a total of 667 responses on precourse survey and 630 responses on postcourse survey (2 questions per learner).

It was expected that the inclusion of role-play sessions observed by peers and faculty who provided direct feedback would bolster both the ability and confidence of medical students in obtaining informed consent, as informed consent role-play exercises have proven successful in the USUHS psychotropic informed consent course as well as in other medical programs when compared with purely didactic courses.[6,11,12] The results of the pre- and postcourse confidence questionnaires and open comments support this hypothesis, with demonstratable improvement in students' comfort in feeling prepared to obtain informed consent from a patient as well as knowledge of the key components of informed consent.

Limitations of this course include the feasibility of implementation and sample size for an entire medical school class. This iteration is designed for 17–20 faculty members to simultaneously facilitate a 4.5-hour course, a faculty burden that may be a potential barrier to implementation at other training sites. One way to overcome this barrier may be to utilize senior residents and fellows of multiple specialties to facilitate small groups. In any format, significant faculty development is required to ensure standardized content delivery.

Future directions include developing procedure and medication vignettes that span a greater breadth of medical specialties, and that are inclusive of a diverse patient population. Additionally, during the in-person iteration of the 2023 course, small group sessions required considerably less time than was allotted for the informed consent role-play, most likely since faculty could "walk-around" the room and observe small groups (comprising 3 students per group) simultaneously. Future administrations of the course will need to carefully optimize session timing in response to the environment (virtual or in-person) and class/faculty size. Additionally, several students opined that an informed consent course would have been of greater benefit prior to the core clerkship year, as it would have afforded them the opportunity to practice obtaining and documenting informed consent with peers before being expected to do so with a patient, although the authors suspect that preclerkship students may not have had enough clinical exposure to fully complete the prework. Perhaps developing an introductory informed consent course prior to the beginning of core clinical clerkships could be considered or implemented. This skill-building exercise may also be adapted to provide longitudinal assessment of students or residents over time to track proficiency in obtaining and documenting informed consent, an essential AAMC EPA skill.

Appendices

A. Course Syllabus.docx
B. Prereadings.pdf
C. StatPearls Article.pdf
D. ADMSEP eModule folder
E. Clinical Vignettes.pdf
F. Rubric.pdf
G. MARRQD, PARRQD Templates.docx
H. Orientation.pptx
I. Observer-Scribe Template.docx
J. Vignette Answers.pdf

All appendices are peer reviewed as integral parts of the Original Publication.

Radhika Tyagi, MD: Resident, Department of Psychiatry, Walter Reed National Military Medical Center; ORCID: https://orcid.org/0009-0005-2744-0537

Elizabeth A. Greene, MD: Assistant Professor, Department of Psychiatry and Family Medicine, Uniformed Services University of the Health Sciences

Louis Lachman, MD: Assistant Professor, Department of Psychiatry and Family Medicine, Uniformed Services University of the Health Sciences

Derrick A. Hamaoka, MD: Professor, Department of Psychiatry, Uniformed Services University of the Health Sciences

Copyright © 2026 Tyagi et al. This is an open-access publication distributed under the terms of the Creative Commons Attribution license.

Philip S. Mullenix, MD, JD: Chief of Surgery and Thoracic Surgeon, Chinle Service Unit/Navajo Nation

Jed Mangal, MD: Assistant Professor, Department of Psychiatry, Emory University School of Medicine

Margaret M. Swanberg, DO: Clinical Associate Professor, Department of Neurology, University of Tennessee College of Medicine

Emily G. Diana, MD: Resident, Department of Psychiatry, Walter Reed National Military Medical Center

Kelly L. Cozza, MD: Professor of Psychiatry and Family Medicine, Uniformed Services University of the Health Sciences

Disclosures
None to report.

Funding/Support
None to report.

Ethical Approval
The Uniformed Services University Human Research Protections Program reviewed this project.

Disclaimer
The opinions and assertions expressed herein are those of the author(s) and do not reflect the official policy or position of the Uniformed Services University of the Health Sciences or the Department of War.

References

1. Roache R. Why is informed consent important? *J Med Ethics*. 2014;40(7):435-436. https://doi.org/10.1136/medethics-2014-102264
2. Obeso V, Biehler JL, Jokela JA, Terhune K. *Core Entrustable Professional Activities for Entering Residency—EPA 11 Toolkit: Obtain Informed Consent for Tests and/or Procedures*. Obeso V, Brown D, Phillipi C, eds. Association of American Medical Colleges; 2017. https://www.aamc.org/what-we-do/mission-areas/medical-education/cbme/core-epas/publications
3. Vossoughi SR, Macauley R, Sazama K, Fung MK. Attitudes, practices, and training on informed consent for transfusions and procedures: a survey of medical students and physicians. *Am J Clin Pathol*. 2015;144(2):315-321. https://doi.org/10.1309/AJCPP85EXSGZORYZ
4. Anderson TN, Aalami LR, Lee EW, et al. Perception and confidence of medical students in informed consent: a core EPA. *Surgery*. 2020;167(4):712-716. https://doi.org/10.1016/j.surg.2019.11.012
5. AlMahmoud T, Hashim MJ, Almahmoud R, Branicki F, Elzubeir M. Informed consent learning: needs and preferences in medical clerkship environments. *PLoS One*. 2018;13(10):e0202466. https://doi.org/10.1371/journal.pone.0202466
6. Diana E, Hamaoka D, Goldenberg M, Cozza KL. Psychotropic medication informed consent: a cross-specialty role-playing skill builder. *MedEdPORTAL*. 2021;17:11152. https://doi.org/10.15766/mep_2374-8265.11152
7. Thomas PA, Kern DE, Hughes MT, Chen BY. *Curriculum Development for Medical Education: A Six-Step Approach.* Johns Hopkins University Press; 2015.
8. Shah P, Thornton I, Kopitnik NL, Hipskind JE. Informed consent. In: StatPearls [Internet]. StatPearls Publishing; November 24, 2024.
9. Hamaoka DA, Meyer EG, Diana EG, Teisberg M, Cozza KL. Informed consent: self-learning module. Association of Directors of Medical Student Education in Psychiatry. Accessed December 11, 2025. https://www.admsep.org/csi-emodules.php?c=informedconsent&v=y
10. Raper SE, Joseph J. Informed consent for academic surgeons: a curriculum-based update. *MedEdPORTAL*. 2020;16:10985. https://doi.org/10.15766/mep_2374-8265.10985
11. Goldman MP, Rudd AV, Baum SC, et al. Formative assessments promote procedural learning and engagement for senior pediatric residents on rotation in the pediatric emergency department. *MedEdPORTAL*. 2022;18:11265. https://doi.org/10.15766/mep_2374-8265.11265
12. deSante-Bertkau J, Herbst LA. Ethics of pediatric and young adult medical decision-making: case-based discussions exploring consent, capacity, and surrogate decision-making. *MedEdPORTAL*. 2021;17:11094. https://doi.org/10.15766/mep_2374-8265.11094

Received: June 27, 2025
Accepted: January 7, 2026
Published: March 3, 2026

Copyright © 2026 Tyagi et al. This is an open-access publication distributed under the terms of the Creative Commons Attribution license.
